# Design, Synthesis, and Evaluation of Novel Cephalotaxine-Cinnamic Acid Derivatives Against Hepatocellular Carcinoma

**DOI:** 10.3390/molecules31193478

**Published:** 2026-09-29

**Authors:** Wenxing Li, Yiming Qian, Hao Wang, Yang Hui, Haofu Dai, Wenhao Chen

**Affiliations:** 1 Key Laboratory of Tropical Medicinal Resource Chemistry of Ministry of Education, College of Chemistry and Chemical Engineering, Hainan Normal University, Haikou 571158, China; 202311070300001@hainnu.edu.cn (W.L.); 202212070300016@hainnu.edu.cn (Y.Q.); 2 Key Laboratory of Tropical Medicinal Plant Chemistry of Hainan Province, College of Chemistry and Chemical Engineering, Hainan Normal University, Haikou 571158, China; 3 Key Laboratory of Research and Development of Natural Product from Li Folk Medicine of Hainan Province, Institute of Tropical Bioscience and Biotechnology, Chinese Academy of Tropical Agricultural Sciences, Haikou 572829, China; wanghao@itbb.org.cn

**Keywords:** *Cephalotaxus hainanensis* Li, Cephalotaxine, liver cancer, PI3K/AKT pathway

## Abstract

Cephalotaxine (**1**) is a parent compound within the cephalotaxine alkaloids class mainly derived from Cephalotaxus plants. In this study, by coupling **1** with cinnamic acid, 17 novel cephalotaxine-cinnamic acid derivatives (**S1**–**S17**) were designed and synthesized to enhance their anti-tumor efficacy. Among them, **S10** showed the strongest cytotoxic activity against Huh7 cells, with the IC_50_ values of 9.3 and 5.9 μM after 24 h and 48 h, respectively, which were 21 and 32 times more potent than those of **1**. In addition, compared with those against AML-12 cells, the proliferation inhibition rates of **S10** against Huh7 cells were 5.0-fold lower, indicating a certain therapeutic window. Network pharmacology screening results indicate that **S10** may exert regulatory effects closely related to cell migration and apoptosis through pathways such as PI3K/AKT. Molecular docking showed that **S10** could effectively bind to the key targets PI3K and AKT. And the Western blot experiment results confirm that **S10** could downregulate the expression of p-PI3K and p-AKT at the protein level while keeping total PI3K and AKT unchanged. Notably, **S10** may regulate liver cancer cells by inhibiting the PI3K/AKT signaling pathway. Mechanistically, **S10** induces S cycle arrest, reduces mitochondrial membrane potential, induces apoptosis, and inhibits migration and invasion. Overall, we reported **S10** as a promising lead compound for the treatment of liver cancer.

## 1. Introduction

Liver cancer is the sixth most common cancer and the third leading cause of cancer-related deaths worldwide [1]. Hepatocellular carcinoma (HCC), the most common subtype of liver cancer, has a high incidence rate and tends to metastasize and recur. Combined with poor treatment outcomes, these factors collectively contribute significantly to the current situation [2,3]. Currently, multiple drugs (such as sorafenib and regorafenib) can improve overall survival rates in patients with hepatocellular carcinoma. However, these drugs face challenges including short duration of efficacy and the development of drug resistance. Therefore, there is an urgent need to develop novel drugs that combine safety, high efficacy, low toxicity, and the ability to reverse drug resistance [3].

The PI3K/AKT pathway is critical for the sustained activation of AKT, which in turn regulates cell cycle progression, apoptosis, metastasis, and angiogenesis. It frequently leads to abnormal signaling and diseases associated with uncontrolled proliferation [4]—a hallmark of numerous human cancers such as breast cancer [5], melanoma [6], lung cancer [7], colorectal cancer [8], prostate cancer [9], liver cancer [10], and gastric cancer [11]. Consequently, PI3K/AKT represents a highly promising therapeutic target in cancer treatment. The PI3K/AKT signaling pathway plays a pivotal role in HCC progression. Its activation enhances the malignant behavior of HCC cells through multiple mechanisms, including promoting glycolysis, inhibiting apoptosis, regulating autophagy, inducing angiogenesis, EMT, and MMP expression. It is also closely associated with drug resistance and radiation resistance in HCC. Multiple natural compounds and small-molecule inhibitors effectively suppress HCC cell proliferation and metastasis by inhibiting the PI3K/AKT signaling pathway, while inducing apoptosis and autophagy to reverse drug resistance [3,12,13,14,15].

Cephalotaxus (Sieb. et Zucc.) belongs to the gymnosperm family Cephalotaxaceae, comprising evergreen trees or shrubs. This family contains only one genus with nine species, seven of which occur in China with three varieties [16,17]. Cephalotaxine alkaloids are a class of natural products derived from Cephalotaxus plants, serving as the primary active constituents within this genus [18]. Notably, their ester alkaloids exhibit significant activities, including antitumor [19], antiviral [20], antibacterial [21], anti-inflammatory [22], antioxidant [23], herbicidal [21], and insecticidal [21] properties. For instance, Harringtonine (HT) and Homoharringtonine (HHT) have been extensively used in China since the 1970 s for treating various types of leukemia [24,25,26,27]. HHT was also approved by the FDA in 2012 for the treatment of chronic myeloid leukemia [28].

*Cephalotaxus hainanensis* Li, commonly known as the Hainan yew, is a species belonging to the genus Cephalotaxus. Also referred to as the red-shell pine or thin-leaf yew, it is endemic to Hainan Island and represents the southernmost distribution of any Cephalotaxaceae species [29]. Due to its potent anti-cancer properties, Hainan yew is also known as the “miracle anti-cancer tree”, serving as the primary raw material for producing cephalotaxine alkaloids used in anti-cancer drugs [30].

Cinnamic acid (CA) is a natural organic acid isolated from cinnamon bark or benzoin [31]. Research has revealed that CA possesses multiple physiological activities, including antiproliferative, anti-inflammatory, antioxidant, antitumor, and anti-aging effects [32,33,34,35]. Precisely because CA possesses multiple advantages such as easy accessibility, high biological activity, and low cytotoxicity, introducing CA into natural products can enhance biological activity while reducing toxic side effects [36,37,38]. Consequently, it has garnered significant attention from medicinal chemists in recent years and is widely employed to modify and enhance the biological activity of promising natural compounds.

**Scheme 1:**
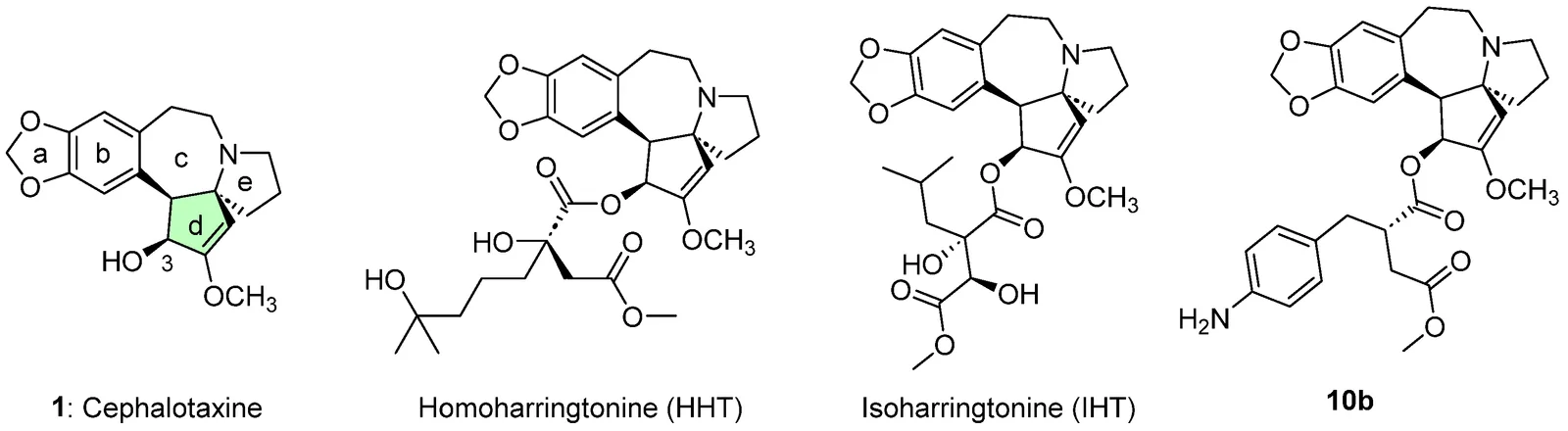
Cephalotaxine and its ester derivatives.

Cephalotaxine is a parent compound as the cephalotaxine alkaloids found in Cephalotaxaceae plants. However, most studies indicate that its anti-tumor activity is substantially lower than that of the ester derivatives of cephalotaxine. Therefore, structural modification of cephalotaxine not only enhances the utilization rate of Cephalotaxaceae plants but also further advances the development of cephalotaxine-based pharmaceuticals. In terms of structural modification, modifications to cephalotaxine primarily occur on its D-ring, more specifically at the C-3 hydroxyl group. Through esterification or acylation reactions, other functional groups are incorporated onto the cephalotaxine backbone to synthesize ester derivatives of cephalotaxine. For example, cephalotaxine derivative **10b** (Scheme 1), obtained by hydroxylating C-3, has shown excellent activity against K562 leukemia cells with an IC_50_ of 121 nM [39]. Research on cephalotaxine ester derivatives has largely centered on leukemia, whereas investigations into their effects on liver cancer remain comparatively limited.

**Scheme 2:**
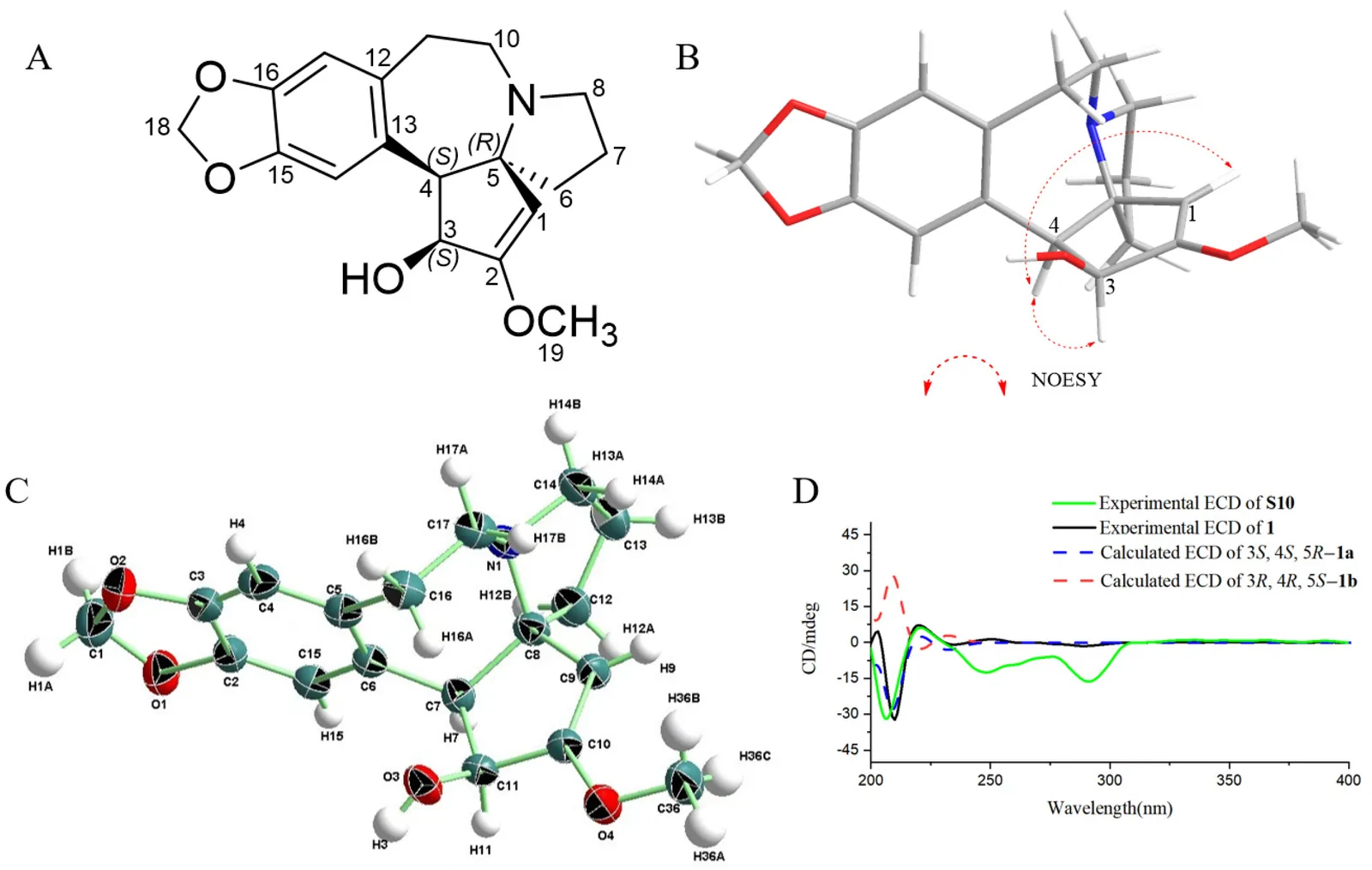
(**A**) Structure of **1**. (**B**) Key NOESY correlations for **1**. (**C**) ORTEP drawing of **1**. (**D**) ECD calculation results.

**Scheme 3:**
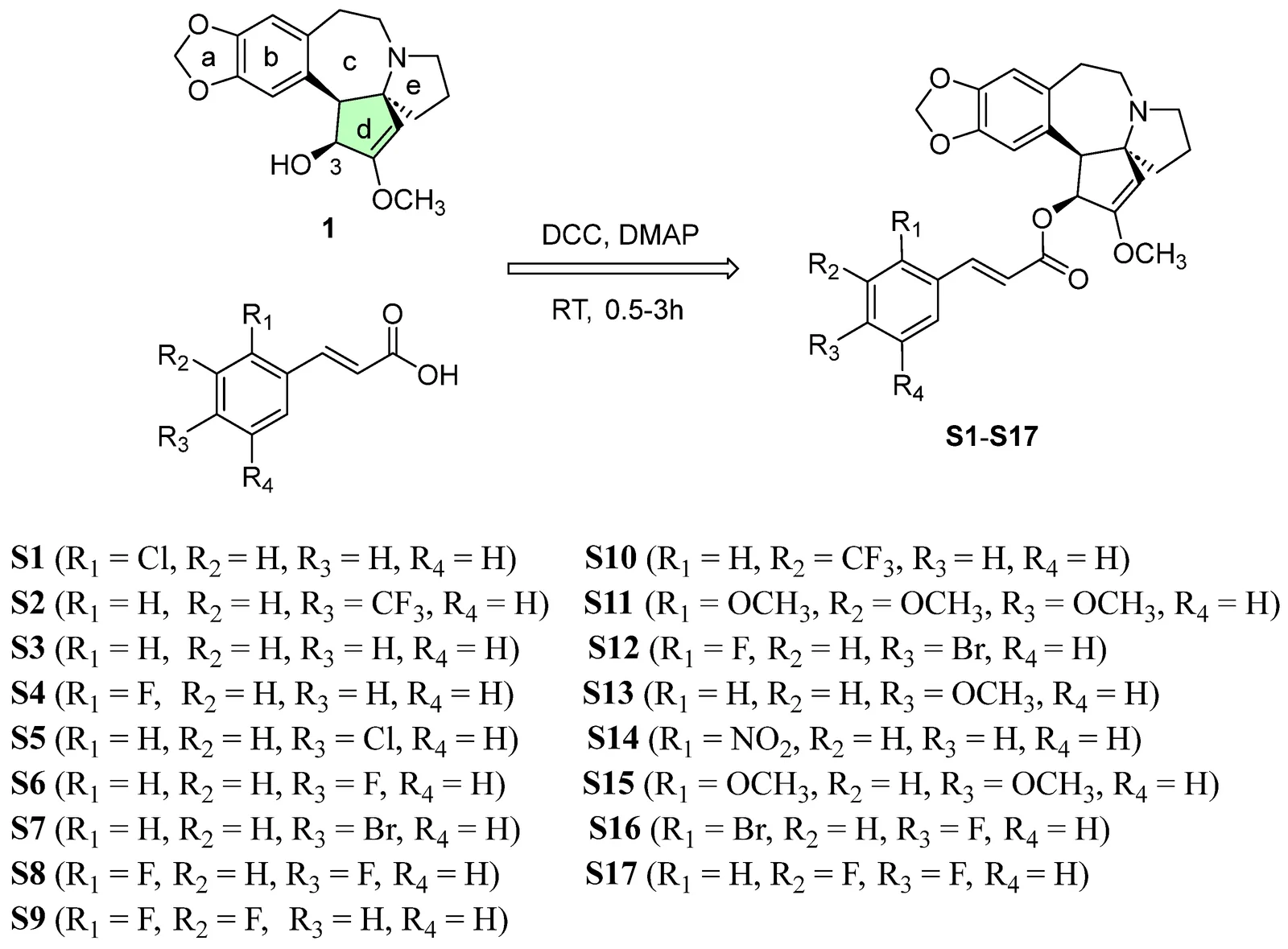
Synthesis approach of derivatives **S1**–**S17**.

Accordingly, after determining the absolute configuration of cephalotaxine **1** (Scheme 2), it was used as the parent compound, and cinnamic acid derivatives were introduced at the C-3 hydroxyl group of the D-ring via esterification, resulting in the design and synthesis of 17 cephalotaxine-cinnamic acid hybrids (**S1**–**S17**) (Scheme 3). Preliminary in vitro antitumor activity testing was conducted on all compounds.

## 2. Results and Discussion

### 2.1. Chemistry

Cephalotaxine (**1**) was a high-yield compound isolated from *Cephalotaxus hainanensis* Li. First, the planar structure of **1** was deduced from its ^1^H- and ^13^C-NMR spectrum (Figures S3 and S4). Subsequently, a NOESY spectrum was employed to ascertain its relative configuration. The NOESY correlations observed between H_1_-H_4_ and H_3_-H_4_ in the NMR spectrum of **1** initially led us to infer that H_1_, H_3_, and H_4_ lie in the same plane (Scheme 2B, Figure S5). Then, single-crystal X-ray crystallography was further confirmed its relative configuration with the Flack parameter of 0.1 (10). Then, ECD calculations were carried out for both possible enantiomers, and in combination with the CD spectroscopy results, the absolute configuration of **1** was ultimately assigned as 3*S*,4*S*,5*R*.

As outlined in Scheme 3, the target ester compounds were synthesized by introducing cinnamic acid derivatives onto the C-3 hydroxyl group of the cephalotaxine (**1**) through esterification reactions. Purify the crude product using preparative chromatography on a plate or column to yield the cephalotaxine-cinnamic acid hybrids (**S1**–**S17**). The target compounds were confirmed by ^1^H and ^13^C NMR, and HRESIMS after chromatographic separation and purification, the purity of them detected by HPLC were >95% purity.

### 2.2. Evaluation of Cytotoxic Activity

Compounds (**S1**–**S17**) were initially screened for cytotoxic activity against three human cancer cell lines: Huh7 (liver), HepG2 (liver), and MDA-MB-231 (breast). All cephalotaxine-cinnamic acid hybrids exhibited varying degrees of enhanced activity, but these activities were lower than those of the positive control drug, adriamycin (Table 1). Furthermore, as shown in **S1** and **S5**, cinnamic acid derivatives substituted with a chlorine atom at the para position exhibit higher reactivity than their ortho-substituted counterparts. For polyhalogenated derivatives, as shown in **S8** and **S16**, replacing the ortho-fluorine atom with a bromine atom increases their reactivity. Among trifluoromethyl substituents, the meta-position confers higher potency than the para-position; notably, the 3-(trifluoromethyl)cinnamic acid hybrid **S10** demonstrates inhibitory activity against Huh7 cells, with IC_50_ values of 9.3 μM and 5.9 μM at 24 h and 48 h, respectively; its potency was 21 and 32 times that of the parent molecule cephalotaxine. Compared with those against AML-12 cells (Table 2), the proliferation inhibition rates of **S10** against Huh7 cells were 5.0-fold lower, indicating certain therapeutic window. This prompted further pharmacological studies to validate its bioactive properties.

### 2.3. Network Pharmacological Antitumor Mechanism of Compound ***S10***

**Figure 1:**
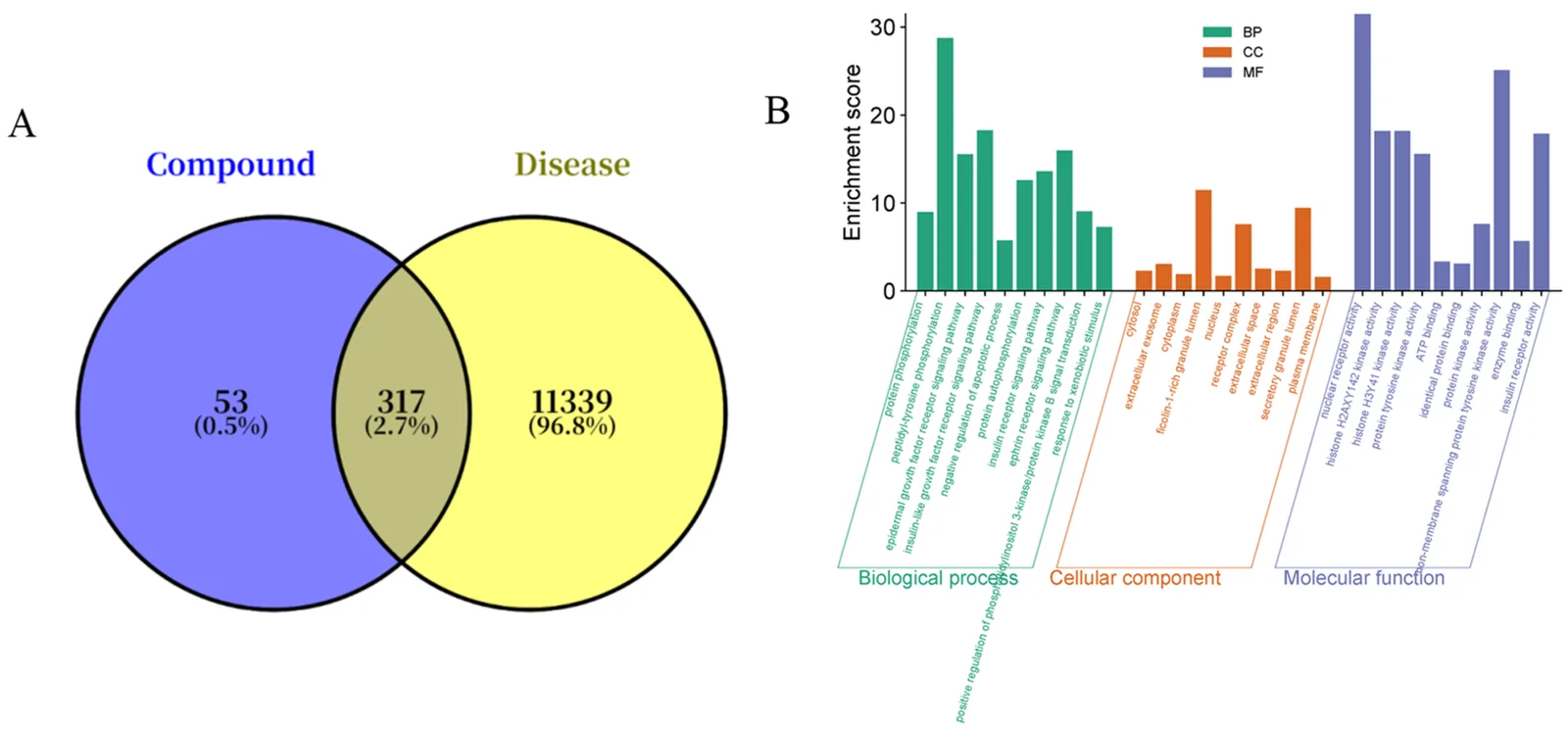
Network pharmacology analysis of **S10** in HCC. (**A**) Venn diagram: drug targets for the treatment of HCC. (**B**) GO enrichment analysis.

A total of 11,574 and 192 targets associated with hepatocellular carcinoma were retrieved from the GeneCards and OMIM databases, respectively. After duplicate analysis, 11,715 unique disease targets were ultimately obtained. Following screening, SwissTargetPrediction and PharmMapper analyses identified 106 and 298 potential therapeutic targets for compound **S10**, respectively, yielding 370 targets after deduplication. Subsequent Venn diagram analysis cross-mapped drug targets with disease targets, revealing 317 key overlapping genes (Figure 1A). The PPI network (Figure S1) comprised 145 nodes and 2703 interaction edges.

Biological process analysis in GO enrichment analysis indicates that this gene primarily participates in the regulation of protein phosphorylation, apoptosis, PI3K/AKT signaling, and other processes. Subcellular localization analysis revealed the target’s distribution in the cytoplasm, nucleus, and receptor complexes. Molecular function analysis showed significant enrichment in nuclear receptor activity and kinase activity, including non-transmembrane protein tyrosine kinase activity and insulin receptor activity (Figure 1B). KEGG pathway enrichment analysis indicates that **S10**’s anti-HCC effects are primarily mediated through core carcinogenic pathways, particularly the PI3K/AKT signaling axis and EGFR tyrosine kinase inhibitor resistance mechanisms (Figure S2). These findings suggest that the therapeutic mechanism of **S10** in HCC may primarily involve regulating cell migration dynamics and apoptosis control.

### 2.4. Molecular Docking Analysis of Compound ***S10***

**Figure 2:**
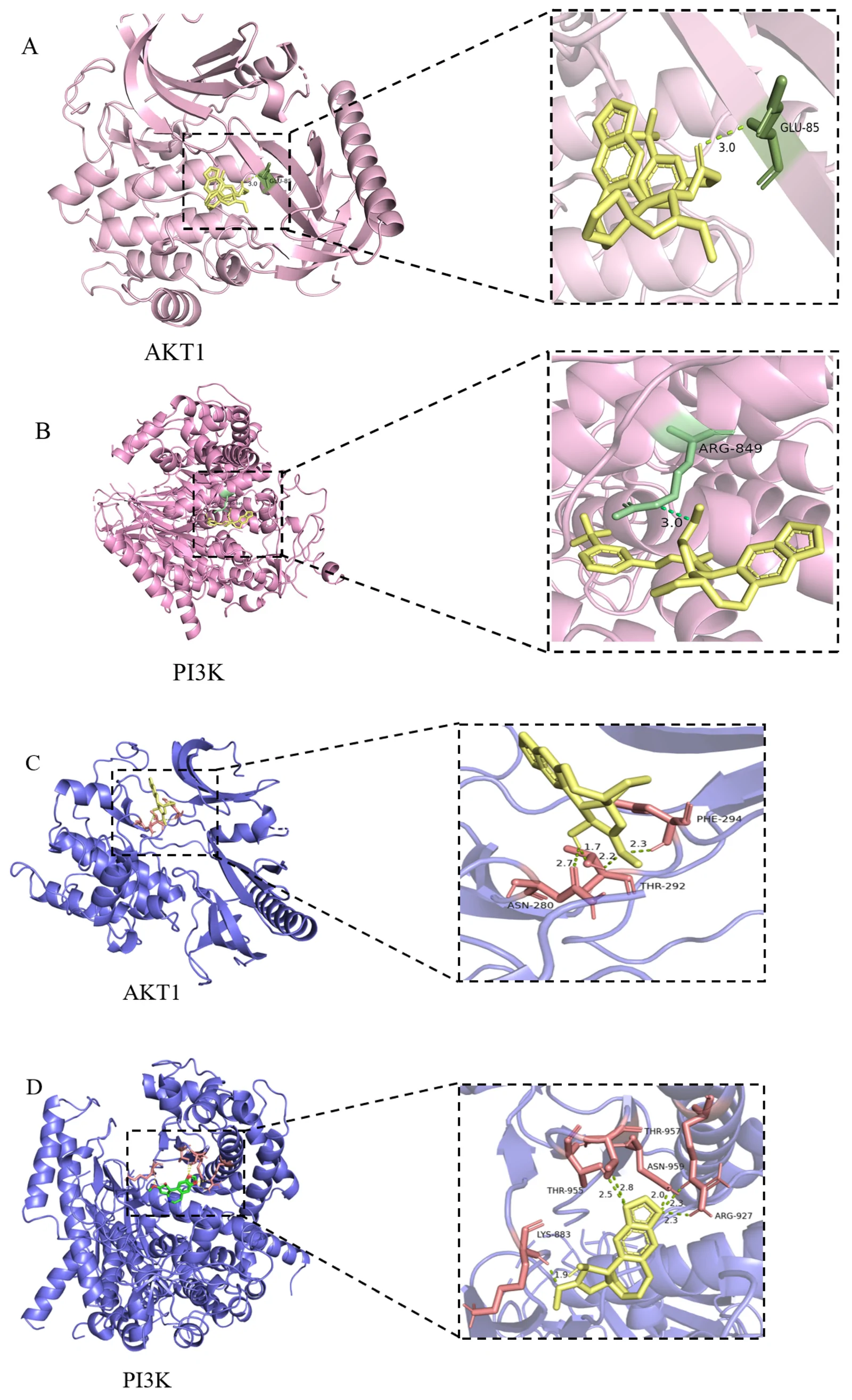
Molecular modeling of **S10** and **1**. (**A**) The results of **S10** docked to AKT1. (**B**) The results of **S10** docked to PI3K. (**C**) The results of **1** docked to AKT1. (**D**) The results of **1** docked to PI3K.

To identify key therapeutic targets for compound **S10** in hepatocellular carcinoma, molecular docking simulations were performed to assess its interactions with key cancer gene regulators. As shown in Figure 2A, the oxygen atom on the carbonyl group of **S10** forms a hydrogen bond with the AKT1 protein at the GLU-85 site, with a docking energy of 9.2 kcal/mol. Additionally, the oxygen atom on the methoxy group of **S10** forms a hydrogen bond with the ARG-849 amino acid of the PI3K protein (Figure 2B), with a docking energy of 8.7 kcal/mol. At the same time, molecular docking results indicate that the hydrogen atom on the hydroxyl group of parent compound **1** forms hydrogen bonds with the ASN-280 and THR-292 amino acids in the AKT1 protein (Figure 2C), while the oxygen atom on the methoxy group forms a hydrogen bond with the PHE-292 amino acid, with a binding energy of 6.3 kcal/mol. In addition, **1** forms hydrogen bonds with the amino acids ASN-959, THR-957, THR-955, LYS-883, and ARG-927 in the PI3K protein, with a binding energy of 6.24 kcal/mol (Figure 2D). The results above indicate that **S10** exhibits strong binding affinity for key regulatory targets, outperforming the parent molecule.

### 2.5. Wound Healing and Transwell Assay

**Figure 3:**
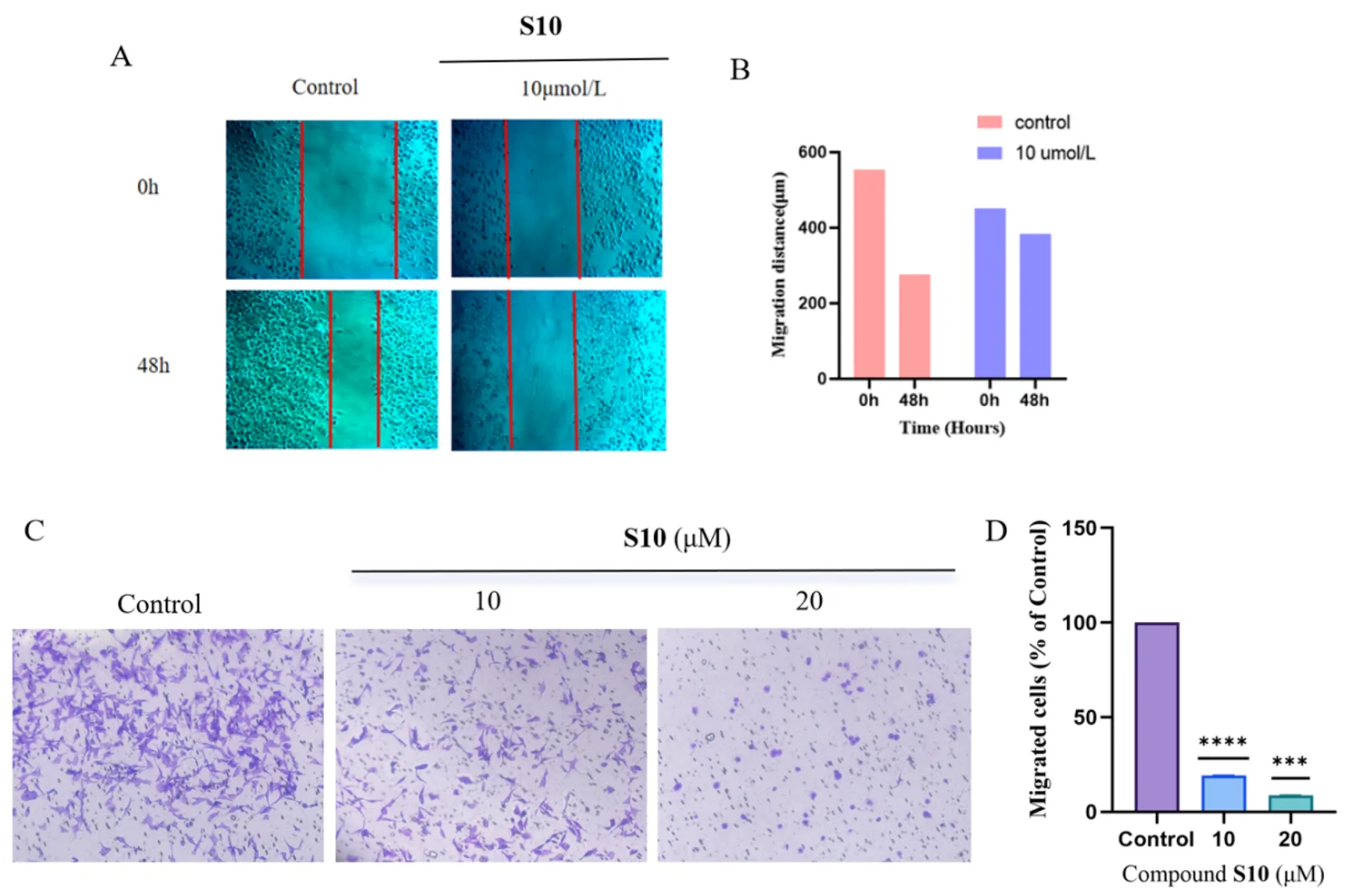
Effect of **S10** on the migration ability of Huh7 cells. (**A**) The migration of Huh7 cells was evaluated using cell scratch assays after incubating the samples for 48 h. (**C**) The invasion of Huh7 cells treated with the samples was assessed using the Transwell assay. (**B**,**D**) Migration measured using Graphpad prism 9. *** *p* < 0.001, **** *p* < 0.0001.

Wound healing and Transwell assays provide a straightforward method for assessing cell migration and motility. As shown in Figure 3A, after 48 h of treatment with compound **S10**, the extent of wound healing in the drug-treated group was significantly reduced compared with the blank control group. In addition, the number of cells migrating through the Transwell chamber was markedly decreased (Figure 3C), indicating that **S10** inhibits tumor cell migration.

### 2.6. Antiproliferative Activity of Compound ***S10***

**Figure 4:**
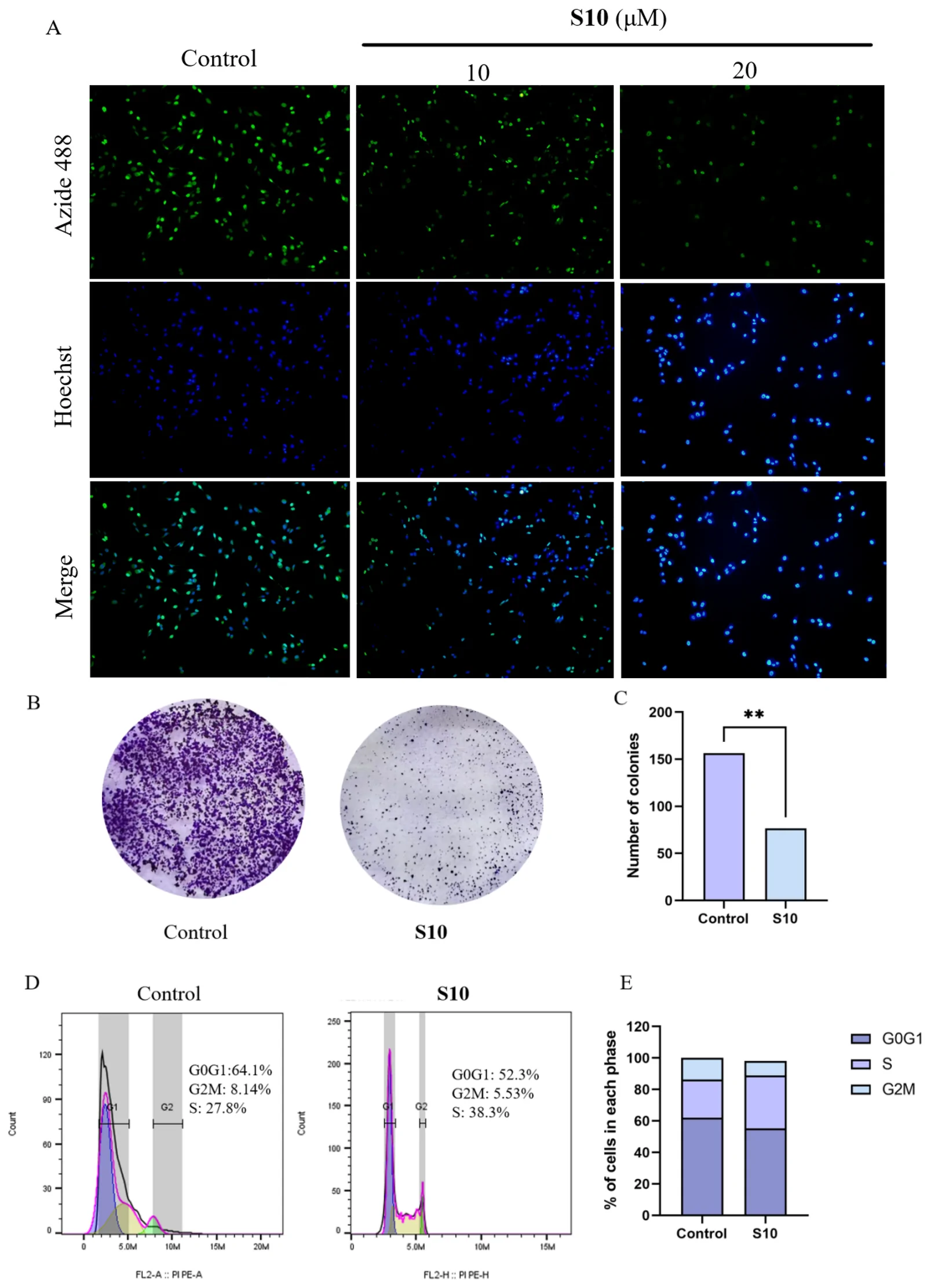
Compound **S10** inhibits Huh7 cells proliferation and induces cell S phase arrest. (**A**) EdU detected the effect of **S10** proliferation of Huh7 cells. (**B**) Representative manifestation of **S10** on the colony formation of Huh7 cells. (**C**) The quantitative analysis of a colony formation for Huh7 cells. (**D**) Huh7 cells were treated with **S10** for 48 h and analyzed by flow cytometry. (**E**) Histograms displayed the percentage of cell cycle distribution. Data were expressed as mean ± SD (*n* = 3); ** *p* < 0.01, compared with the control group.

Cell proliferation is essential for maintaining physiological tissue homeostasis, but its pathological dysregulation has become a key therapeutic target in oncology. To systematically evaluate the antiproliferative effect of **S10**, through EdU treatment analysis, we found that **S10** affected DNA synthesis in Huh7 cells (Figure 4A). Subsequently, we conducted standardised colony formation assays in Huh7 cells. Treatment with drug **S10** demonstrated an inhibitory effect on colony formation (Figure 4B,C). Meanwhile, flow cytometry analysis revealed that treatment with compound **S10** could arrest the Huh7 cell cycle at the S phase (Figure 4D,E), and the proportion increased from 27.8% to 38.3%. The number of cells in the G0/G1 phase and G2/M phase decreased in a dose-dependent manner accordingly. These findings confirm that **S10** can effectively inhibit the proliferative ability of HCC, highlighting its therapeutic significance in controlling tumor growth.

### 2.7. Induced Huh7 Cell Apoptosis by Compound ***S10***

**Figure 5:**
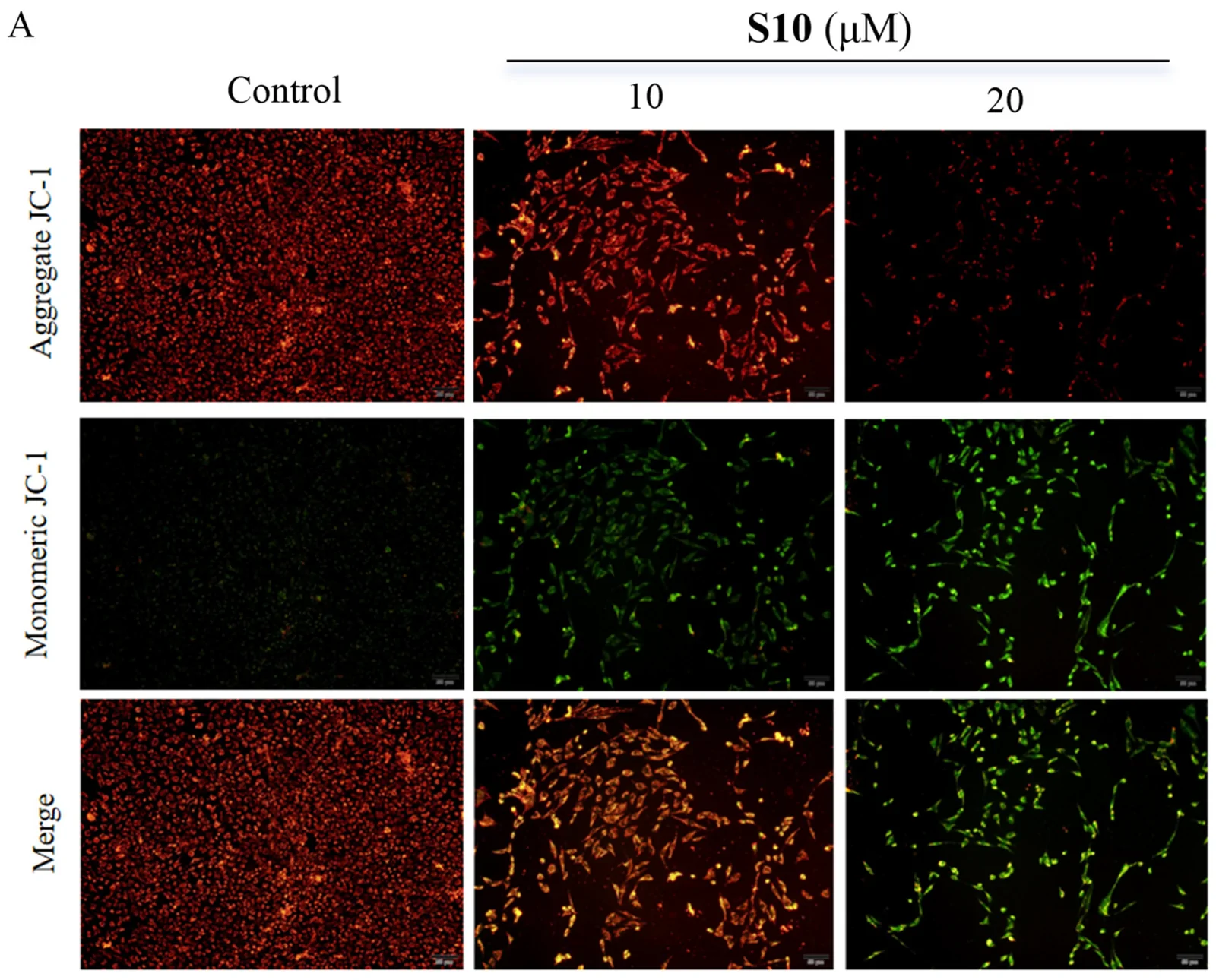

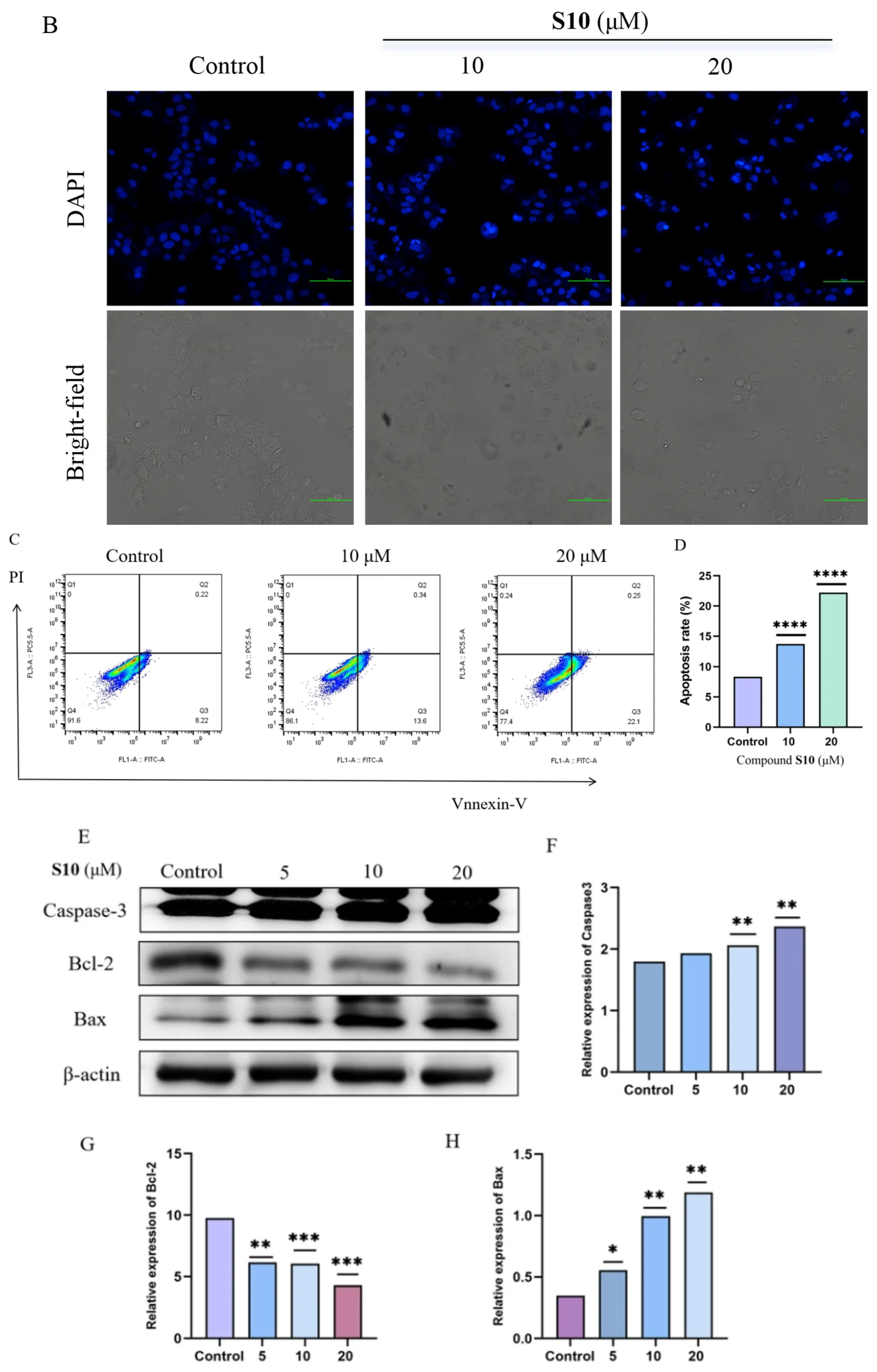
Compound **S10** induced Huh7 cells apoptosis. (**A**) **S10** reduces mitochondrial membrane potential of Huh7 cells. (**B**) DAPI staining showed that **S10** could induce nuclear shrinkage in Huh7 cells. (**C**,**D**) Cell apoptosis of control and administered groups was assayed by flow cytometry. (**E**–**H**) Western blot analyzed the levels of the apoptosis-related proteins Bcl-2, Bax, Caspase-3 in Huh7 cells after incubated respectively with different concentrations of **S10** for 48 h. Data were expressed as mean ± SD (*n* = 3); * *p* < 0.05, ** *p* < 0.01, *** *p* < 0.001, **** *p* < 0.0001, compared with the control group.

Apoptosis is a form of programmed cell death mechanism crucial for development and tissue homeostasis, and it represents a strategic therapeutic approach in oncology. Mitochondrial membrane potential decrease is an early event of apoptosis and can be detected by the shift in JC-1 fluorescence from red aggregates to green monomers. Huh7 cells treated with **S10** for 48 h were examined, and the results showed that the green fluorescence intensity increased in a concentration-dependent manner (Figure 5A), confirming the dose-dependent induction effect of **S10** on early apoptotic events. These pieces of evidence prove that the compound can initiate a mitochondria-dependent apoptotic pathway in Huh7 cells.

DAPI stain is a cell dye that causes cell nuclei to emit blue fluorescence. In this experiment, the nuclei of normal cells exhibit blue fluorescence upon interaction with DAPI stain, while the cytoplasm remains non-fluorescent. When cells undergo apoptosis, the nuclei appear intensely stained or fragmented with dense staining. As shown in Figure 5B and Figure S10 exhibits a significant inhibitory effect on the hepatocellular carcinoma cell line Huh7, inducing cell rupture and apoptosis.

To quantitatively assess **S10**-induced apoptosis, we employed Annexin V-FITC/PI double staining for flow cytometric analysis. Dose–response analysis revealed apoptosis induction in Huh7 cells after 48 h exposure to **S10**, with apoptosis rates increasing from 8.44% (0 μM) to 13.94% (10.0 μM) and 22.35% (20.0 μM) (Figure 5C,D). Consistent with these phenotypic observations, Western blot analysis revealed that **S10** downregulated the expression of the apoptosis-related protein Bcl-2 in a dose-dependent manner, while simultaneously upregulating the expression of Bax and Caspase-3 (Figure 5E–H). These results collectively indicate that **S10** induces apoptosis in Huh7 cells.

### 2.8. Compound ***S10*** Inhibited PI3K/AKT Pathway Activation in Huh7 Cells

**Figure 6:**
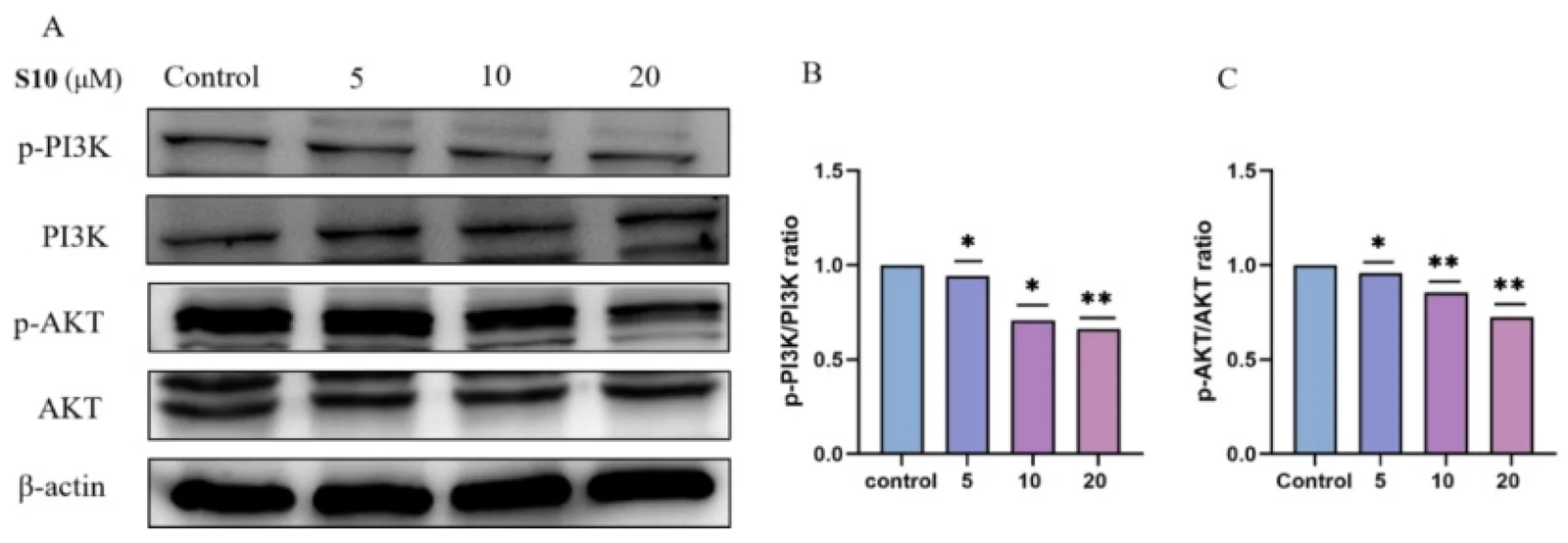
(**A**) Compound **S10** may inhibits the expression of PI3K/AKT pathway. (**B**,**C**) The protein expression levels of p-PI3K, PI3K, p-AKT and AKT were quantitatively analyzed by Image J. Data were expressed as mean ± SD (*n* = 3); * *p* < 0.05, ** *p* < 0.01, compared with the control group.

The regulation of the PI3K/AKT signaling pathway by compound **S10** in Huh7 cells was investigated via Western blot. As shown in Figure 6A–C, treatment with **S10** downregulates the expression of p-PI3K and p-AKT at the protein level while maintaining the overall levels of PI3K and AKT in Huh7 cells unchanged. This indicates that **S10** exerts its regulatory effect by inhibiting the activation of the PI3K/AKT signaling pathway.

### 2.9. ADME Predictions for ***S1***–***S17***

The pharmacological effects of a compound, together with its low toxicological impact, are not by themselves sufficient to classify it as a potential drug candidate. During drug development, the pharmacokinetic properties of drug candidates should be evaluated as early as possible [40]. The ADME profile of the molecule was assessed to determine its physicochemical properties, including water solubility, hydrophobicity, the ability to cross the blood–brain barrier, and its behavior in the gastrointestinal environment before excretion from the body. In this study, we used the SwissADME online tool (http://www.swissadme.ch/, accessed on 11 May 2026) to predict the drug-like properties of compounds **S1**–**S17** (Table 3). ADME prediction revealed that all compounds exhibit high gastrointestinal absorption rates. Differences in HBA, rotatable bonds, and LogP are primarily attributed to variations in substituents. In **S11** and **S15**, CA is substituted with multiple methoxy groups, resulting in higher Csp^3^ values and lower lipophilicity, whereas in **S14**, CA is substituted with a nitro group, yielding the highest tPSA value and the lowest LogP value in this series. In addition, ADME prediction results indicate that **S10** largely complies with Lipinski’s five rules. The Fraction Csp3 value of 0.39 suggests that it possesses good drug-like properties, while the TPSA value of 57.23 Å^2^, HBD value of 0, HBA value of 9, Log *p* value of 4.64, and Rotor value of 6 indicate good oral bioavailability. Furthermore, the prediction results suggest that **S10** is well absorbed in the gastrointestinal tract and exhibits excellent drug-like characteristics.

## 3. Experiment

### 3.1. General Experimental Procedures

1D and 2D NMR spectra were recorded on a JNM-ECZ600R-600 MHz NMR spectrometer (JEOL, LTD, JP). HR-ESIMS spectrometer were measured on ESI-QTOF mass spectrometer (Billerica, MA, USA). The melting point was determined using an SGW X-4 microscopic melting point analyzer (YDWG, Shanghai, China); the results were not corrected. Semi-Preparative HPLC was performed on a Labsolution LC-20AT (SGLC, Shanghai, China) series with a DAD detector using an Agilent Eclipse XDBC18 column (9.4 × 250 mm, 5 μm) (Agilent Corporation, Santa Clara, CA, USA). Thin-layer chromatography (TLC) was performed using pre-coated silica gel plates (Shanghai Haohong Bio-Pharmaceutical Technology Co., Ltd. (Shanghai, China); G60, F254). Silica gel (Qingdao Haiyang Chemical Group Co., Ltd., Qingdao, China; 300–400 mesh) and octadecylsilylated silica gel (YMC; 12 nm–50 μm) were used for column chromatography (CC). All solvents were sourced from Xilong Chemical Reagent Factory (Guangzhou, China). The parent compound cephalotaxine was provided by researcher Prof. Haofu Dai.

### 3.2. Spectral Data for Compound ***1***

Cephalotaxine (**1**): white crystals; m.p. 134.8–135.6 °C; ^1^H NMR (400 MHz, CDCl_3_) *δ*: 6.67 (1H, s, H-17), 6.64 (1H, s, H-14), 5.90 (2H, s, H-18), 4.92 (1H, s, H-1), 4.75 (1H, dd, *J* = 3.2, 9.6 Hz, H-3), 3.73 (3H, s, H-19), 3.67 (1H, d, *J* = 9.6 Hz, H-4), 3.35 (1H, m, H-11a), 3.07 (1H, m, H-8a), 2.91 (1H, m, H-10a), 2.61 (1H, m, H-8b), 2.56 (1H, m, H-10b), 2.35 (1H, m, H-11b), 2.01 (1H, m, H-6a), 1.87 (1H, m, H-6b), 1.74 (2H, m, H-7). ^13^C NMR (100 MHz, CDCl_3_) *δ*: 160.6 (C-2), 147.0 (C-15), 146.2 (C-16), 134.4 (C-13), 128.1 (C-12), 112.7 (C-14), 110.4 (C-17), 101.0 (C-18), 97.8 (C-1), 73.4 (C-3), 70.7 (C-5), 58.2 (C-19), 57.3 (C-4), 54.0 (C-8), 48.7 (C-10), 43.7 (C-6), 31.8 (C-11), 20.4 (C-7). The crystal structure of **1** was determined by crystallization in a mixture of petroleum ether and EtOAc. Crystal data for **1** (CCDC: 2582785): C_18_H_21_NO_4_, M = 315.14, *a* = 19.537 (6) Å, *b* = 8.1357 (15) Å, *c* = 22.113 (7) Å, α = 90°, β = 113.77 (4) °, γ = 90°,*V* = 3216.64 (183) Å^3^, *T* = 293 (2) K, space group *P*1, *Z* = 4, *μ* (Mo Kα) = 0.71073 mm^−1^, 7103 reflections measured, 41,832 independent reflections (R_int_ = 0.0501). The final *R*_1_ values were 0.0767 (*I* > 2*σ* (*I*)). The final w*R* (*F*^2^) values were 0.1967 (*I* > 2*σ* (*I*)). The final *R*_1_ values were 0.1392 (all data). The final w*R* (*F*^2^) values were 0.2641 (all data). The goodness of fit on *F*^2^ was 1.054. Flack parameter = 0.1 (10).

### 3.3. General Procedure for Synthesis of Cephalotaxine-Cinnamic Acid Derivatives

The specific steps are as follows: In a 25 mL round-bottom flask, sequentially add cephalotaxine (**1**) (1.0 equiv, 0.2 mmol), DMAP (1.5 equiv, 0.3 mmol), DCC (2 equiv 0.4 mmol), and the corresponding cinnamic acid derivative (2 equiv 0.4 mmol), followed by 4–5 mL of dry CH_2_Cl_2_. React at room temperature for 0.5–3 h, monitoring the reaction progress using thin-layer chromatography. After the reaction is complete, transfer the reaction mixture to a 250 mL round-bottom flask and quench the reaction with 50 mL of petroleum ether. Concentrate on the solution under reduced pressure to obtain the crude product. The crude product was purified by thin-layer chromatography (TLC) using a mobile phase of petroleum ether/EtOAc (3:1, *v*/*v*) containing triethylamine, yielding the cephalotaxine-cinnamic acid adducts **S1**–**S17**.

2-Methoxy-1,5,6,8,9,14b-hexahydro-4*H*-[1,3]dioxolo[4′,5′:4,5]benzo[1,2-*d*]cyclopenta[*b*]pyrrolo[1,2-*a*]azepin-1-yl (*E*)-3-(2-chlorophenyl) acrylate (**S1**). Brown oil, yield: 76%, ^1^H NMR (600 MHz, CDCl_3_) *δ*: 7.65 (1H, d, *J* = 16.0 Hz), 7.43 (1H, dd, *J* = 7.8, 1.8 Hz), 7.36 (1H, dd, *J* = 8.0, 1.4 Hz), 7.26 (1H, m), 7.21 (1H, m), 6.60 (1H, s), 6.56 (1H, s), 6.03 (1H, d, *J* = 16.0 Hz), 5.99 (1H, d, *J* = 9.5 Hz), 5.81 (1H, d, *J* = 1.6 Hz), 5.78 (1H, d, *J* = 1.6 Hz), 5.10 (1H, s), 3.83 (1H, d, *J* = 9.5 Hz), 3.74 (3H, s), 3.38 (1H, m), 3.10 (1H, m), 3.03 (1H, m), 2.73 (m, 1H), 2.67 (1H, m), 2.48 (1H, dd, *J* = 14.6, 6.9 Hz), 2.09 (1H, m), 1.98 (1H, m), 1.80 (2H, m). ^13^C NMR (150 MHz, CDCl_3_) *δ*: 165.3, 158.5, 146.9, 145.9, 140.1, 134.8, 133.0, 132.7, 131.1, 130.0, 127.8, 127.6, 127.1, 120.1, 113.3, 109.9, 100.9, 99.8, 74.6, 71.7, 57.6, 56.2, 53.7, 48.3, 42.9, 31.1, 20.3. HR-ESI-MS *m*/*z* calcd. for C_27_H_27_ClNO_5_ [M + H]^+^ 480.1572, found: 480.1570.

2-Methoxy-1,5,6,8,9,14b-hexahydro-4*H*-[1,3]dioxolo[4′,5′:4,5]benzo[1,2-*d*]cyclopenta[*b*]pyrrolo[1,2-*a*]azepin-1-yl (*E*)-3-(trifluoromethyl) phenyl) acrylate (**S2**). Brown oil, yield: 59%, ^1^H NMR (600 MHz, CDCl_3_) *δ*: 7.58 (2H, d, *J* = 8.1 Hz), 7.45 (2H, d, *J* = 8.1 Hz), 7.24 (1H, d, *J* = 16.0 Hz), 6.60 (1H, s), 6.57 (1H, s), 6.11 (1H, d, *J* = 16.0 Hz), 5.97 (1H, d, *J* = 9.5 Hz), 5.80 (1H, d, *J* = 1.5 Hz), 5.75 (1H, d, *J* = 1.5 Hz), 5.10 (1H, s), 3.84 (1H, d, *J* = 9.5 Hz), 3.74 (3H, s), 3.33 (1H, m), 3.09 (1H, m), 2.97 (1H, m), 2.66 (1H, m), 2.61 (1H, m), 2.42 (1H, dd, *J* = 14.4, 7.0 Hz), 2.06 (1H, m), 1.94 (1H, m), 1.77 (2H, m). ^13^C NMR (150 MHz, CDCl_3_) *δ*: 165.4, 157.7, 146.7, 145.8, 142.6, 137.9, 133.5, 131.8,131.6, 128.2, 128.1, 125.9 (q, *J* = 31.2 Hz), 125.8, 124.8, 120.2, 113.3, 109.6, 100.8, 100.4, 74.8, 70.9, 57.5, 56.6, 53.9, 48.6, 43.3, 31.6, 20.4. HR-ESI-MS *m*/*z* calcd. for C_28_H_27_F_3_NO_5_ [M + H]^+^ 514.1836, found: 514.1832.

2-Methoxy-1,5,6,8,9,14b-hexahydro-4*H*-[1,3]dioxolo[4′,5′:4,5]benzo[1,2-*d*]cyclopenta[*b*]pyrrolo[1,2-*a*]azepin-1-yl cinnamate (**S3**). Brown oil, yield: 72%, ^1^H NMR (600 MHz, CDCl_3_) *δ*: 7.37 (2H, m), 7.33 (2H, d, *J* = 2.3 Hz), 7.32 (1H, d, *J* = 1.6 Hz), 7.26 (1H, d, *J* = 16.0 Hz), 6.60 (1H, s), 6.57 (1H, s), 6.05 (1H, d, *J* = 16.0 Hz), 5.98 (1H, d, *J* = 9.6 Hz), 5.80 (1H, d, *J* = 1.6 Hz), 5.75 (1H, d, *J* = 1.6 Hz), 5.08 (1H, s), 3.83 (1H, d, *J* = 9.6 Hz, 1H), 3.74 (3H, s), 3.36 (1H, m), 3.10 (1H, m), 2.99 (1H, m), 2.68 (1H, m), 2.62 (1H, m), 2.43 (1H, dd, *J* = 14.4, 7.0 Hz), 2.07 (1H, m), 1.94 (1H, m), 1.78 (2H, m). ^13^C NMR (150 MHz, CDCl_3_) *δ*: 165.9, 158.1, 146.7, 145.8, 144.5, 134.5, 133.4, 130.2, 128.9 (2 × C), 128.2, 128.1 (2 × C), 117.6, 113.3, 109.7, 100.8, 100.0, 74.5, 71.1, 57.5, 56.5, 53.8, 48.5, 43.2, 31.4, 20.3. HR-ESI-MS *m*/*z* calcd. for C_27_H_28_NO_5_ [M + H]^+^ 446.1962, found: 446.1960.

2-Methoxy-1,5,6,8,9,14b-hexahydro-4*H*-[1,3]dioxolo[4′,5′:4,5]benzo[1,2-*d*]cyclopenta[*b*]pyrrolo[1,2-*a*]azepin-1-yl (*E*)-3-(2-fluorophenyl) acrylate (**S4**). Brown oil, yield: 56%, ^1^H NMR (600 MHz, CDCl_3_) *δ*: 7.41 (1H, d, *J* = 16.2 Hz), 7.37 (1H, s), 7.31 (1H, m), 7.10 (1H, dd, *J* = 7.6, 1.1 Hz), 7.04 (1H, dd, *J* = 10.1, 7.7 Hz), 6.61 (1H, s), 6.56 (1H, s), 6.13 (1H, d, *J* = 16.2 Hz), 5.98 (1H, d, *J* = 9.5 Hz), 5.82 (1H, d, *J* = 1.6 Hz), 5.78 (1H, d, *J* = 1.6 Hz), 5.12 (1H, s), 3.83 (1H, d, *J* = 9.5 Hz), 3.76 (3H, s), 3.41 (1H, m), 3.14 (1H, m), 3.10 (1H, m), 2.91 (1H, brs), 2.83 (1H, brs), 2.51 (1H, dd, *J* = 14.9, 6.7 Hz, 1H), 2.12 (2H,m), 1.86 (2H, m). ^13^C NMR (150 MHz, CDCl_3_) *δ*: 165.4, 162.1, 160.4, 159.8, 147.3, 146.3, 137.2, 131.9, 131.8, 128.7, 127.0, 124.5, 122.3, 119.6, 116.3, 113.3, 109.9, 101.0, 98.9, 74.3, 71.0, 57.8, 55.7, 53.3, 47.7, 42.7, 30.3. HR-ESI-MS *m*/*z* calcd. for C_27_H_27_FNO_5_ [M + H]^+^ 464.1868, found: 464.1869.

2-Methoxy-1,5,6,8,9,14b-hexahydro-4*H*-[1,3]dioxolo[4′,5′:4,5]benzo[1,2-*d*]cyclopenta[*b*]pyrrolo[1,2-*a*]azepin-1-yl (*E*)-3-(4-chlorophenyl) acrylate (**S5**). Brown oil, yield: 85%, ^1^H NMR (600 MHz, CDCl_3_) *δ*: 7.26 (4H, s), 7.16 (1H, d, *J* = 16.0 Hz), 6.57 (1H, s, 6.53 (1H, s), 5.98 (1H, d, *J* = 16.0 Hz), 5.94 (1H, d, *J* = 9.5 Hz), 5.77 (1H, d, *J* = 1.6 Hz), 5.72 (1H, d, *J* = 1.6 Hz), 5.06 (1H, s), 3.80 (1H, d, *J* = 9.5 Hz), 3.70 (3H, s), 3.30 (1H, m), 3.06 (1H, m), 2.95 (1H, m, 2.64 (1H, m), 2.59 (1H, m), 2.39 (1H, dd, *J* = 14.4, 7.0 Hz), 2.05 (1H, m Hz), 1.92 (1H, m), 1.74 (2H, m). ^13^C NMR (150 MHz, CDCl_3_) *δ*: 165.6, 158.0, 146.7, 145.8, 143.1, 136.1, 133.3, 132.9, 129.2 (2 × C), 129.1 (2 × C), 128.2, 118.1, 113.3, 109.6, 100.8, 100.1, 74.6, 71.1, 57.5, 56.5, 53.8, 48.5, 43.2, 31.4, 20.3. HR-ESI-MS *m*/*z* calcd. for C_27_H_27_ClNO_5_ [M + H]^+^ 480.1572, found: 480.1574.

2-Methoxy-1,5,6,8,9,14b-hexahydro-4*H*-[1,3]dioxolo[4′,5′:4,5]benzo[1,2-*d*]cyclopenta[*b*]pyrrolo[1,2-*a*]azepin-1-yl (*E*)-3-(4-fluorophenyl) acrylate (**S6**). Brown oil, yield: 54%, ^1^H NMR (600 MHz, CDCl_3_) *δ*: 7.34 (2H, dd, *J* = 8.6, 5.4 Hz), 7.20 (1H, d, *J* = 16.0 Hz), 7.01 (1H, t, *J* = 8.6 Hz), 6.59 (1H, s), 6.56 (1H, s), 5.97 (1H, d, *J* = 9.6 Hz), 5.96 (1H, d, *J* = 16.0 Hz), 5.79 (1H, d, *J* = 1.6 H), 5.74 (1H, d, *J* = 1.6 Hz), 5.08 (1H, s), 3.83 (1H, d, *J* = 9.6 Hz), 3.73 (1H, s), 3.33 (1H, m), 2.97 (1H, m), 2.64 (1H, m), 2.60 (1H, m), 2.41 (1H, dd, *J* = 14.4, 7.0 Hz), 2.06 (1H, m), 1.92 (1H, m), 1.76 (1H, m). ^13^C NMR (150 MHz, CDCl_3_) *δ*: 165.8, 164.7, 163.0, 157.9, 146.6, 145.7, 143.2, 133.5, 129.9 (2 × C), 128.3, 117.4, 116.1, 115.9, 113.3, 109.6, 100.8, 100.2, 74.6, 70.8, 57.5, 56.6, 53.9, 48.6, 43.3, 31.6, 20.4. HR-ESI-MS *m*/*z* calcd. for C_27_H_27_FNO_5_ [M + H]^+^ 464.1868, found: 464.1869.

2-Methoxy-1,5,6,8,9,14b-hexahydro-4*H*-[1,3]dioxolo[4′,5′:4,5]benzo[1,2-*d*]cyclopenta[*b*]pyrrolo[1,2-*a*]azepin-1-yl (*E*)-3-(4-bromophenyl) acrylate (**S7**). Brown oil, yield: 62%, ^1^H NMR (600 MHz, CDCl_3_) *δ*: 7.44 (2H, d, *J* = 8.4 Hz), 7.21 (2H, d, *J* = 8.7 Hz), 7.18 (1H, s), 6.61 (1H, s), 6.55 (1H, s), 6.02 (1H, d, *J* = 15.9 Hz), 5.95 (1H, d, *J* = 9.2 Hz), 5.81 (1H, d, *J* = 1.4 Hz), 5.76 (1H, d, *J* = 1.4 Hz), 5.12 (1H, s), 3.83 (1H, d, *J* = 9.2 Hz), 3.75 (3H, s), 3.38 (1H, m), 3.10 (2H, m), 2.86 (2H, m), 2.48 (1H, dd, *J* = 14.7, 6.6 Hz), 2.13 (2H, m), 1.85 (2H, m). ^13^C NMR (150 MHz, CDCl_3_) *δ*: 165.4, 159.8, 147.3, 146.3, 143.5, 133.2, 132.1 (2 × C), 132.0, 129.4 (2 × C), 127.0, 124.6, 117.8, 113.3, 109.8, 101.0, 98.9, 74.3, 71.0, 57.8, 55.6, 53.3, 47.6, 42.0, 30.4, 20.1. HR-ESI-MS *m*/*z* calcd. for C_27_H_27_BrNO_5_ [M + H]^+^ 524.1067, found: 524.1071.

2-Methoxy-1,5,6,8,9,14b-hexahydro-4*H*-[1,3]dioxolo[4′,5′:4,5]benzo[1,2-*d*]cyclopenta[*b*]pyrrolo[1,2-*a*]azepin-1-yl (*E*)-3(2,5-difluorophenyl) acrylate (**S8**). Brown oil, yield: 59%, ^1^H NMR (600 MHz, CDCl_3_) *δ*:7.31 (1H, d, *J* = 16.2 Hz), 7.05 (1H, ddd, *J* = 8.3, 5.3, 2.7 Hz), 7.00 (2H, ddd, *J* = 9.2, 4.6, 2.7 Hz), 6.59 (1H, s), 6.56 (1H, s), 6.09 (1H, d, *J* = 16.2 Hz), 5.97 (1H, d, *J* = 9.5 Hz), 5.81 (1H, d, *J* = 1.6 Hz), 5.78 (1H, d, *J* = 1.6 Hz), 5.09 (1H, s), 3.82 (1H, d, *J* = 9.5 Hz), 3.73 (3H, s), 3.34 (1H, m), 3.11 (1H, m), 2.99 (1H, m), 2.69 (1H, m), 2.64 (1H, m), 2.44 (1H, dd, *J* = 14.5, 7.0 Hz), 2.08 (1H, m), 1.96 (1H, m), 1.78 (2H, m). ^13^C NMR (150 MHz, CDCl_3_) *δ*: 165.2, 158.0, 157.9, 156.4, 146.8, 145.8, 135.6, 133.2, 127.9, 123.7, 121.0, 118.2, 117.4, 114.4, 113.3, 109.8, 100.9, 100.1, 74.8, 71.3, 57.6, 56.4, 53.8, 48.5, 43.1, 31.3, 20.3. HR-ESI-MS *m*/*z* calcd. for C_27_H_26_F_2_NO_5_ [M + H]^+^ 482.1774, found: 482.1775.

2-Methoxy-1,5,6,8,9,14b-hexahydro-4*H*-[1,3]dioxolo[4′,5′:4,5]benzo[1,2-*d*]cyclopenta[b]pyrrolo[1,2-*a*]azepin-1-yl (*E*)-3-(2,4-difluorophenyl) acrylate (**S9**). Brown oil, yield: 54%, ^1^H NMR (600 MHz, CDCl_3_) *δ*: 7.36 (1H, dt, *J* = 8.5, 6.3 Hz), 7.31 (1H, d, *J* = 16.2 Hz), 6.85 (1H, dt, *J* = 8.7, 2.9 Hz), 6.80 (1H, ddd, *J* = 11.0, 8.7, 2.5 Hz), 6.59 (1H, s), 6.56 (1H, s), 6.06 (1H, d, *J* = 16.2 Hz), 5.97 (1H, d, *J* = 9.5 Hz), 5.80 (1H, d, *J* = 1.6 Hz), 5.76 (1H, d, *J* = 1.6 Hz), 5.08 (1H, s), 3.82 (1H, d, *J* = 9.5 Hz), 3.73 (3H, s), 3.34 (1H, ddd, *J* = 14.3, 12.2, 7.8 H), 3.10 (1H, m), 2.97 (1H, m), 2.65 (1H, m), 2.60 (1H, m), 2.42 (1H, dd, *J* = 14.3, 7.2 Hz), 2.06 (1H, m), 1.92 (1H, m), 1.77 (2H, m). ^13^C NMR (150 MHz, CDCl_3_) *δ*: 165.6, 163.1, 160.6, 157.8, 146.7, 145.7, 135.8, 133.5, 129.8, 128.2, 119.6, 119.0, 113.3, 112.1, 109.7, 104.6, 100.8, 100.3, 74.7, 70.9, 57.5, 56.5, 53.9, 48.6, 43.3, 31.5, 20.3. HR-ESI-MS *m*/*z* calcd. for C_27_H_26_F_2_NO_5_ [M + H]^+^ 482.1774, found: 482.1774.

Cephalotaxine (63.0 mg, 0.2 mmol), 3-(trifluoromethyl)cinnamic acid (86.5 mg, 0.4 mmol), DCC (82.5 mg, 0.4 mmol), and DMAP (36.7 mg, 0.3 mmol) were dissolved in anhydrous CH_2_Cl_2_ (4 mL), and the mixture was stirred at room temperature for 2 h. After TLC verification that the reaction had gone to completion, the crude product was purified by column chromatography (CC) using petroleum ether/EtOAc (3:1) as the eluent, affording 42.8 mg of **S10**.

2-Methoxy-1,5,6,8,9,14b-hexahydro-4*H*-[1,3]dioxolo[4′,5′:4,5]benzo[1,2-*d*]cyclopenta[*b*]pyrrolo[1,2-*a*]azepin-1-yl(*E*)-3-(trifluoromethyl) phenyl) acrylate (**S10**). Brown oil, yield: 68%, ^1^H NMR (600 MHz, CDCl_3_) *δ*: 7.58 (1H, s), 7.56 (1H, d, *J* = 7.8 Hz), 7.52 (1H, d, *J* = 7.8 Hz), 7.44 (1H, t, *J* = 7.8 Hz), 7.23 (1H, d, *J* = 16.0 Hz), 6.59 (1H, s), 6.56 (1H, s), 6.09 (1H, d, *J* = 16.0 Hz), 5.96 (1H, d, *J* = 9.5 Hz), 5.78 (1H, d, *J* = 1.4 Hz), 5.74 (1H, d, *J* = 1.4 Hz), 5.09 (1H, s), 3.83 (1H, d, *J* = 9.5 Hz), 3.72 (3H, s), 3.33 (1H, m), 3.08 (1H, m), 2.96 (1H, td, *J* = 11.6, 7.0 Hz), 2.66 (1H, m), 2.61 (1H, m), 2.42 (1H, dd, *J* = 14.4, 7.0 Hz), 2.06 (1H, m), 1.93 (1H, m), 1.76 (2H, m). ^13^C NMR (150 MHz, CDCl_3_) *δ*: 165.3, 157.8, 146.7, 145.7, 142.6, 135.2, 133.4, 131.2, 130.9, 129.4, 128.2, 126.5 (q, *J* = 3.0 Hz), 124.5 (q, *J* = 3.0 Hz), 122.9, 119.5, 113.2, 109.6, 100.8, 100.3, 74.8, 71.0, 57.4, 56.1, 53.9, 48.5, 43.2, 31.5, 20.3. HR-ESI-MS *m*/*z* calcd. for C_28_H_27_F_3_NO_5_ [M + H] ^+^ 514.1836, found: 514.1834. CD spectroscopic analysis of **S10** revealed a negative cotton effect at λ_max_ 207 (∆ε − 9.61) nm and a positive cotton effect at λ_max_ 221(∆ε + 1.79) nm, consistent with those observed for the parent compound **1**. Consequently, **S10** is inferred to possess the same absolute configuration as **1**.

2-Methoxy-1,5,6,8,9,14b-hexahydro-4*H*-[1,3]dioxolo[4′,5′:4,5]benzo[1,2-*d*]cyclopenta[*b*]pyrrolo[1,2-*a*]azepin-1-yl(*E*)-3-(2,3,4-trimethoxyphenyl) acrylate (**S11**). Brown oil, yield: 51%, ^1^H NMR (600 MHz, CDCl_3_) *δ*: 7.48 (1H, d, *J* = 16.1 Hz), 7.08 (1H, d, *J* = 8.8 Hz), 6.62 (1H, d, *J* = 8.8 Hz), 6.60 (1H, s), 6.54 (1H, s), 6.01 (1H, d, *J* = 16.1 Hz), 5.97 (1H, d, *J* = 9.2 Hz), 5.80 (1H, d, *J* = 2.4), 5.76 (1H, d, *J* = 2.4), 5.09 (1H, s), 3.85 (3H, s), 3.83 (6H, m), 3.82 (1H, d, *J* = 9.2) 3.74 (3H, s), 3.41 (1H, m), 3.09 (1H, m), 3.05 (1H, m), 2.82 (1H, m), 2.75 (1H, m), 2.47 (1H, m), 2.09 (1H, m), 2.02 (1H, m), 1.81 (2H, m). ^13^C NMR (150 MHz, CDCl_3_) *δ*: 166.2, 159.4, 155.5, 153.2, 147.0, 146.0, 142.3, 139.6, 132.5, 127.4, 123.2, 121.4, 116.2, 113.3, 109.8, 107.6, 101.3, 99.1, 74.1, 72.5, 61.4, 61.0, 57.7, 56.1, 56.0, 53.4, 47.9, 42.4, 30.7, 20.2. HR-ESI-MS *m*/*z* calcd. for C_30_H_34_NO_8_ [M + H]^+^ 536.2279, found: 536.2277.

2-Methoxy-1,5,6,8,9,14b-hexahydro-4*H*-[1,3]dioxolo[4′,5′:4,5]benzo[1,2-*d*]cyclopenta[*b*]pyrrolo[1,2-*a*]azepin-1-yl(*E*)-3-(4-bromo-2-fluorophenyl) acrylate (**S12**). Brown oil, yield: 72%, ^1^H NMR (600 MHz, CDCl_3_) *δ*: 7.28 (1H, d, *J* = 16.1 Hz), 7.24 (2H, m), 7.22 (1H, m), 6.58 (1H, s), 6.55 (1H, s), 6.11 (1H, d, *J* = 16.1 Hz), 5.97 (1H, d, *J* = 9.5 Hz), 5.80 (1H, d, *J* = 1.6 Hz), 5.76 (1H, d, *J* = 1.6 Hz), 5.08 (1H, s), 3.82 (1H, d, *J* = 9.5 Hz), 3.72 (3H, s), 3.33 (1H, ddd, *J* = 14.3, 12.2, 7.8 Hz), 3.09 (1H, td, *J* = 9.0, 4.7 Hz), 2.95 (1H, m), 2.63 (1H, m), 2.59 (1H, m), 2.42 (1H, dd, *J* = 14.3, 7.0 Hz), 2.06 (1H, m), 1.92 (1H, m), 1.76 (2H, m). ^13^C NMR (150 MHz, CDCl_3_) *δ*: 165.4, 161.6, 157.7, 146.7, 145.7, 135.6, 133.6, 129.6, 128.2, 127.9, 124.4, 121.7, 120.6, 120.0, 113.2, 109.7, 100.8, 100.4, 74.8, 70.8, 57.5, 56.6, 53.9, 48.6, 43.4, 31.5, 20.3. HR-ESI-MS *m*/*z* calcd. for C_27_H_26_BrFNO_5_ [M + H]^+^ 542.0973, found: 542.0971.

2-Methoxy-1,5,6,8,9,14b-hexahydro-4*H*-[1,3]dioxolo[4′,5′:4,5]benzo[1,2-*d*]cyclopenta[*b*]pyrrolo[1,2-*a*]azepin-1-yl (*E*)-3-(4-methoxyphenyl) acrylate (**S13**). Brown oil, yield: 67%, ^1^H NMR (600 MHz, CDCl_3_) *δ*: 7.31 (2H, d, *J* = 8.8 Hz), 7.23 (1H, d, *J* = 16.0 Hz), 6.83 (2H, d, *J* = 8.8 Hz), 6.61 (1H, s), 6.56 (1H, s), 5.96 (1H, d, *J* = 9.4 Hz), 5.91 (1H, d, *J* = 16.0 Hz), 5.82 (1H, d, *J* = 1.6 Hz), 5.76 (1H, d, *J* = 1.6 Hz), 5.11 (1H, s), 3.81 (1H, d, *J* = 9.4 Hz), 3.79 (3H, s), 3.75 (3H, s), 3.40 (1H, m), 3.12 (1H, m), 3.06 (1H, m), 2.91 (1H, brs), 2.81 (1H, m), 2.49 (1H, dd, *J* = 14.8, 6.8 Hz), 2.11 (2H, m), 1.85 (2H, m). ^13^C NMR (150 MHz, CDCl_3_) *δ*: 166.0, 161.4, 159.8, 147.2, 146.2, 144.6, 132.0, 129.7 (2 × C), 127.1, 127.0, 114.6, 114.3, 113.3, 109.8, 101.0, 98.8, 74.0, 73.2, 69.2, 57.8, 55.8, 55.4, 53.2, 47.6, 42.1, 30.5, 20.2. HR-ESI-MS *m*/*z* calcd. for C_28_H_30_NO_6_ [M + H]^+^ 476.2066, found: 476.2066.

2-Methoxy-1,5,6,8,9,14b-hexahydro-4*H*-[1,3]dioxolo[4′,5′:4,5]benzo[1,2-*d*]cyclopenta[*b*]pyrrolo[1,2-*a*]azepin-1-yl (*E*)-3-(4-nitrophenyl) acrylate (**S14**). Brown oil, yield: 55%, ^1^H NMR (600 MHz, CDCl_3_) *δ*: 8.18 (2H, d, *J* = 8.7 Hz), 7.50 (2H, d, *J* = 8.7 Hz), 7.24 (1H, d, *J* = 16.0 Hz), 6.59 (1H, s), 6.57 (1H, s), 6.15 (1H, d, *J* = 16.0 Hz), 5.97 (1H, d, *J* = 9.5 Hz), 5.79 (1H, d, *J* = 1.5 Hz), 5.75 (1H, d, *J* = 1.5 Hz), 5.10 (1H, s), 3.84 (1H, d, *J* = 9.5 Hz), 3.73 (3H, s), 3.31 (1H, ddd, *J* = 14.2, 12.2, 7.8 Hz), 3.09 (1H, m), 2.95 (1H, m), 2.63 (1H, m), 2.63 (1H, m), 2.42 (1H, dd, *J* = 14.3, 7.0 Hz), 2.05 (1H, m), 1.92 (1H, m), 1.76 (2H, m). ^13^C NMR (150 MHz, CDCl_3_) *δ*: 165.0, 157.4, 148.5, 146.7, 145.7, 141.4, 140.6, 133.6, 128.6, 128.4, 124.2, 121.9, 113.3, 109.6, 100.8, 100.7, 75.1, 70.7, 57.5, 56.7, 54.0, 48.7, 43.5, 31.7, 20.4. HR-ESI-MS *m*/*z* calcd. for C_27_H_27_N_2_O_7_ [M + H]^+^ 491.1813, found: 491.1812.

2-Methoxy-1,5,6,8,9,14b-hexahydro-4*H*-[1,3]dioxolo[4′,5′:4,5]benzo[1,2-*d*]cyclopenta[*b*]pyrrolo [1,2-*a*]azepin-1-yl (*E*)-3-(2,4-dimethoxyphenyl) acrylate (**S15**). Brown oil, yield: 51%, ^1^H NMR (600 MHz, CDCl_3_) *δ*: 7.51 (1H, d, *J* = 16.0 Hz), 7.27 (1H, d, *J* = 8.6 Hz), 6.59 (1H, s), 6.55 (1H, s), 6.43 (1H, dd, *J* = 8.5, 2.3 Hz), 6.39 (1H, d, *J* = 2.3 Hz), 6.01 (1H, d, *J* = 16.0 Hz), 5.98 (1H, d, *J* = 9.6 Hz), 5.80 (1H, d, *J* = 1.5 Hz), 5.74 (1H, d, *J* = 1.5 Hz), 5.06 (1H, s), 3.81 (6H, m), 3.80 (1H, d, *J* = 9.6 Hz), 3.72 (3H, s), 3.39 (1H, m), 3.08 (1H, m), 2.96 (1H, m), 2.66 (1H, m), 2.60 (1H, m), 2.42 (1H, dd, *J* = 14.5, 7.0 Hz), 2.07 (1H, m), 1.91 (1H, m), 1.76 (2H, m).^13^C NMR (150 MHz, CDCl_3_) *δ*: 166.7, 162.7, 159.7, 158.3, 146.6, 145.6, 139.6, 133.5, 130.0, 128.3, 116.6, 115.3, 113.1, 109.7, 105.2, 100.7, 99.8, 98.4, 74.0, 70.9, 57.4, 56.5, 55.5 (2 × C), 53.8, 48.5, 43.2, 31.5, 20.3. HR-ESI-MS *m*/*z* calcd. for C_29_H_32_NO_5_ [M + H]^+^ 506.2173, found: 506.2171.

2-Methoxy-1,5,6,8,9,14b-hexahydro-4*H*-[1,3]dioxolo[4′,5′:4,5]benzo[1,2-*d*]cyclopenta[*b*]pyrrolo[1,2-*a*]azepin-1-yl(*E*)-3-(2-bromo-4-fluorophenyl) acrylate (**S16**). Brown oil, yield: 63%, ^1^H NMR (600 MHz, CDCl_3_) *δ*: 7.54 (1H, d, *J* = 15.9 Hz), 7.40 (1H, dd, *J* = 8.8, 5.9 Hz), 7.30 (1H, dd, *J* = 8.1, 2.6 Hz), 6.99 (1H, m), 6.59 (1H, s), 6.56 (1H, s), 5.98 (1H, d, *J* = 9.5 Hz), 5.93 (1H, d, *J* = 15.9 Hz), 5.80 (1H, d, *J* = 1.6 Hz), 5.78 (1H, d, *J* = 1.6 Hz), 5.08 (1H, s), 3.83 (1H, d, *J* = 9.5 Hz), 3.73 (3H, s), 3.34 (1H, ddd, *J* = 14.4, 12.3, 7.8 Hz), 3.08 (1H, m), 2.94 (1H, m), 2.62 (1H, dd, *J* = 9.9, 7.0 Hz), 2.58 (1H, m), 2.45 (1H, dd, *J* = 14.4, 7.0 Hz), 2.06 (1H, m), 1.92 (1H, m), 1.76 (2H, m). ^13^C NMR (150 MHz, CDCl_3_) *δ*: 165.2, 163.1, 157.6, 146.6, 145.6, 141.3, 133.6, 130.9, 128.9, 128.3, 125.4, 120.6, 120.3, 115.3, 113.3, 109.8, 100.8, 100.4, 74.8, 70.7, 57.4, 56.6, 54.0, 48.7, 43.4, 31.6, 20.3. HR-ESI-MS *m*/*z* calcd. for C_27_H_26_BrFNO_5_ [M + H]^+^ 542.0973, found: 542.0972.

2-Methoxy-1,5,6,8,9,14b-hexahydro-4*H*-[1,3]dioxolo[4′,5′:4,5]benzo[1,2-*d*]cyclopenta[*b*]pyrrolo[1,2-*a*]azepin-1-yl(*E*)-3-(3,4-difluorophenyl) acrylate (**S17**). Brown oil, yield: 84%, ^1^H NMR (400 MHz, CDCl_3_). *δ* 7.22 (1H, d, *J* = 15.6 Hz), 7.15 (2H, m), 7.13 (1H, m), 6.68 (1H, s), 6.60 (1H, s), 5.99 (1H, d, *J* = 9.2 Hz), 5.98 (1H, d, *J* = 15.6 Hz), 5.89 (1H, d, *J* = 1.5 Hz), 5.85 (1H, d, *J* = 1.5 Hz), 5.22 (1H, s), 3.87 (1H, d, *J* = 9.2 Hz), 3.83 (3H, s), 3.48 (1H, m), 3.36 (2H, m), 3.17 (2H, m), 2.62 (1H, m), 2.31 (2H, m), 2.02 (2H, m). ^13^C NMR (100 MHz, CDCl_3_) *δ* 164.9, 162.3, 151.9, 150.2, 148.2, 147.1, 143.2, 131.3, 129.9, 125.3, 125.0, 118.0, 117.7, 116.5, 113.6, 110.2, 101.4, 97.1, 74.0, 71.0, 58.4, 54.4, 52.7, 46.6, 40.3, 28.9, 18.9. HR-ESI-MS *m*/*z* calcd. for C_27_H_26_F_2_NO_5_ [M + H]^+^ 482.1770, found: 482.1774.

### 3.4. Cell Culture and Cell Viability Assay

Human breast cancer MDA-MB-231 and alpha mouse liver (AML-12) cells were purchased from Precell (catalog numbers CL-0150B and CL0601, respectively), whereas human hepatocellular carcinoma Huh7 and Hep G2 cells were sourced from BDBIO (catalog numbers C5176 and C5175, respectively). MDA-MB-231, AML-12, and Huh7 cells were maintained in DMEM complete medium (containing 10% fetal bovine serum and 1% penicillin–streptomycin), while Hep G2 cells were maintained in MEM complete medium at 37 °C in a 5% CO_2_ incubator. Cell viability was evaluated with the CCK-8 assay [41]. Logarithmic-phase cells were seeded into 96-well plates and treated with the test compounds for 24 and 48 h. After incubation, 10 μL of CCK-8 reagent was added per well, and the cells were cultured for an additional 1–4 h. Absorbance was read at 450 nm on a microplate reader. The half-maximal inhibitory concentration (IC_50_) values were determined by nonlinear regression using GraphPad Prism 9 software.

### 3.5. Wound-Healing Migration Assay

Logarithmic-phase cells were seeded into a 6-well plate at 3 × 10^6^ cells/well and cultured for 24 h. Once the cell monolayer reached confluence, mechanical wounds were created with a sterile 10 μL pipette tip [42]. Following PBS washing, the cells were exposed to compound **S10** at different concentrations and incubated for 48 h. Images were captured under a microscope at 0 and 48 h, respectively. ImageJ software was then used for image processing and data analysis.

### 3.6. Transwell Migration Assay

The Transwell chambers were transferred into a sterile 24-well plate. Huh7 cells that had been serum-starved for 5 h were resuspended in serum-free medium. Various concentrations of **S10** were then added to this serum-free cell suspension. Each upper chamber received 200 μL of the cell suspension, corresponding to approximately 40,000 cells per well. The lower chamber was filled with 600 μL of DMEM supplemented with 20% fetal bovine serum. The plate was incubated at 37 °C in a 5% CO_2_ incubator for 48 h [43]. After incubation, the medium was removed from both chambers. The cells were fixed with 4% paraformaldehyde for 20–30 min and stained with 0.1% crystal violet for 30 min. Cells remaining in the upper chamber were wiped away, and after air-drying, images were captured under a microscope and analyzed using ImageJ software.

### 3.7. Colony Formation Assay

For colony formation, Huh7 cells were plated in 6-well plates at 1000 cells per well and cultured continuously in the presence of **S10** for 14 days. The medium was then discarded, and the cells were rinsed with PBS, fixed in 10% formaldehyde for 20 min, and stained with 0.1% crystal violet for 15 min. Colony images were captured and analyzed with ImageJ using standardized threshold settings [44].

### 3.8. Observation of Morphological Changes in Huh7 Cells by DAPI Staining

After treating Huh7 cells with **S10** for 24 h, add 4% paraformaldehyde and fix at room temperature for 20 min. Aspirate the paraformaldehyde, wash cells 2–3 times with PBS, add DAPI staining solution, and incubate in the dark for 10 min. Capture images using a confocal microscope or under blue light in a fluorescence microscope [45].

### 3.9. EdU Staining

To evaluate cell proliferation, Huh7 cells were incubated with 20 μM EdU for 2 h. Subsequently, the cells were fixed with 4% paraformaldehyde and permeabilized using 0.3% Triton X-100. EdU incorporation was detected with the Click™ EdU-488 Cell Proliferation Detection Kit (Beyotime, Shanghai, China) following the manufacturer’s instructions. Nuclei were counterstained with Hoechst 33342. Fluorescence images were acquired using an inverted fluorescence microscope.

### 3.10. Cell Apoptosis Analysis

Cells were seeded at a density of 2 × 10^5^ cells per well in a six-well plate and cultured for 24 h. Subsequently, cells were treated with **S10** for 24 h. After treatment, cells were harvested and washed twice with PBS. Cells were then resuspended in 1× binding buffer and stained with Annexin V-FITC and propidium iodide (PI) for 15 min at room temperature in the dark. The stained cell samples were diluted with 400 μL of 1× binding buffer and analyzed using a flow cytometer. The collected data was processed and analyzed using FlowJo V10 software [44].

### 3.11. Cell Cycle Arrest Analysis

Log-phase Huh7 cells were plated into a 6-well plate at 2.0 × 10^5^ cells per well. After overnight culture in a thermostatic incubator, the cells were exposed to different concentrations of **S10** for 24 h. They were then collected, resuspended in PBS, and fixed with 1 mL of 80% ethanol at 4 °C for at least 2 h, followed by two washes with PBS. Cell cycle distribution was assessed by PI staining using the Beyotime C1052 kit (Beyotime, Shanghai, China) according to the provided protocol, and fluorescence was acquired by flow cytometry. FlowJo V10 software was used for data analysis.

### 3.12. Mitochondrial Membrane Assay

To evaluate mitochondrial membrane potential, Huh7 cells were plated at 1 × 10^4^ cells per well in 6-well plates and incubated until they adhered. Following 48 h of exposure to **S10** at various concentrations, JC-1 staining was carried out according to the established procedure. Briefly, the medium was replaced with JC-1 working solution, and the cells were incubated at 37 °C for 20 min. The supernatant was then removed, and the cells were rinsed twice with JC-1 staining buffer [44]. Finally, fluorescence was observed under a fluorescence microscope, and images were captured for documentation.

### 3.13. Network Pharmacology and Bioinformatics Analysis

Compound **S10** was submitted to the Swiss Target Prediction Database (http://www.swisstargetprediction.ch/, accessed on 13 April 2026) for target prediction. Concurrently, hepatocellular carcinoma-related targets were retrieved from public databases such as GeneCards and OMIM. Following target integration and deduplication, a compound-target interaction network was constructed using Cytoscape 3.7.2. The curated target set underwent Gene Ontology (GO) biological process annotation and Kyoto Encyclopedia of Genes and Genomes (KEGG) pathway enrichment analysis via the Metascape platform to elucidate the potential therapeutic mechanisms of **S10** in intervening hepatocellular carcinoma.

### 3.14. Molecular Docking

The high-resolution crystal structures of two key therapeutic targets, AKT1 (PDB ID: 9C1W) and PI3K (PDB ID: 3DPD), were obtained from the Protein Data Bank (https://www.rcsb.org/, accessed on 18 April 2026). Molecular docking was carried out following previously established protocols. Receptor preparation began with structural refinement in PyMOL 2.3.4, after which hydrogen atoms were added and charges were neutralized using AutoDock Vina 1.1.2. Tools. The protein and ligand were then converted into pdbqt files. Receptor–ligand docking was performed with AutoDock Vina 1.1.2. The docking outcomes were evaluated using PLIP and visualized in PyMOL.

### 3.15. Western Blot Analysis

For Western blot analysis, Huh7 cells were plated in 6-well plates at 4 × 10^5^ cells/mL and exposed to **S10**. Following lysis with RIPA buffer, the lysate was centrifuged at 12,000× *g* for 20 min at 4 °C to collect the supernatant. Protein concentration was measured using a BCA protein assay kit (Beyotime, Shanghai, China). Proteins were resolved by SDS-PAGE and then transferred onto PVDF membranes. Immunoblotting was carried out with specific antibodies against Bcl-2, Bax, Caspase-3, p-PI3K, PI3K, p-AKT, and AKT, and protein bands were visualized with an ECL detection kit (Beyotime, Shanghai, China). β-actin served as the internal control.

### 3.16. ADME Prediction

ADME profiling of the compounds were conducted using SwissADME (http://www.swissadme.ch/, accessed on 11 May 2026), enabling assessment of its physicochemical parameters, lipophilicity, aqueous solubility, pharmacokinetic behavior, drug-likeness, and medicinal chemistry attributes [40].

### 3.17. Statistical Analysis

Statistical evaluation was conducted using GraphPad Prism 9.0 software (GraphPad Software, Inc., San Diego, USA), and each analysis was performed at least three times. Values are presented as the mean ± standard deviation. The threshold for statistical significance was defined as *p* < 0.05. Significance levels are denoted as follows: * *p* < 0.05, ** *p* < 0.01, *** *p* < 0.001, **** *p* < 0.0001, and ns for not significant.

## 4. Conclusions

In this study, 17 new cephalotaxine-cinnamic acid derivatives were synthesized to enhance their anti-tumor activity. Among them, compound **S10** showed the highest inhibitory activity against Huh7. **S10** exhibited IC_50_ values against Huh7 cells of 9.3 μM and 5.9 μM at 24 h and 48 h, respectively; its potency was 21 and 32 times that of the parent molecule cephalotaxine. Compared to AML-12 cells, the proliferation inhibition rates of **S10** against Huh7 cells were 5.0-fold lower, indicating certain therapeutic window. However, as this magnitude of increase was not deemed sufficiently significant, we omitted further activity testing in normal cells. Mechanistically, **S10** regulates the proliferation, migration, and invasion of Huh7 cells through the PI3K/AKT signaling pathway, induces mitochondrial dysfunction, and leads to apoptosis. In conclusion, modifying cephalotaxine-cinnamic acid derivatives yielded the antitumor derivative **S10**, which exhibits enhanced potency compared to its parent compound and shows promise for further research on hepatocellular carcinoma.

## Figures and Tables

**Table 1:** Cytotoxic effects of compounds **S1**–**S17**, with IC_50_ values (μM).

IC_50_ (μM)			
Compounds	Huh7	Hep G2	MDA-MB-231
**S1**	40.1 ± 0.60	95.7 ± 1.77	85.8 ± 1.06
**S2**	23.7 ± 0.49	60.0 ± 0.60	34.0 ± 0.28
**S3**	91.5 ± 1.40	109.5 ± 1.10	70.6 ± 0.22
**S4**	118.0 ± 1.24	127.2 ± 2.48	123.0 ± 3.10
**S5**	22.9 ± 0.40	42.9 ± 1.25	18.2 ± 0.27
**S6**	92.5 ± 2.43	126.0 ± 1.42	121.3 ± 2.36
**S7**	35.6 ± 0.11	83.7 ± 0.50	84.1 ± 2.00
**S8**	136.2 ± 2.94	158.5 ± 1.00	160.8 ± 4.05
**S9**	98.9 ± 1.49	129.0 ± 4.67	171.6 ± 1.89
**S10**	9.3 ± 0.28	36.3 ± 0.14	34.5 ± 0.50
**S11**	112.3 ± 1.03	122.9 ± 2.46	86.0 ± 0.46
**S12**	19.9 ± 0.12	43.7 ± 0.29	55.6 ± 0.45
**S13**	72.6 ± 0.71	119.9 ± 2.03	47.4 ± 0.14
**S14**	71.1 ± 1.66	72.9 ± 1.99	57.3 ± 0.98
**S15**	42.2 ± 1.42	97.2 ± 1.07	75.8 ± 0.40
**S16**	16.2 ± 0.16	31.6 ± 0.32	36.8 ± 0.33
**S17**	77.7 ± 1.13	122.8 ± 1.01	106.4 ± 3.11
**1**	196.0 ± 0.91	125.3 ± 2.80	112.5 ± 2.20
Adriamycin	1.24 ± 0.32	1.73 ± 0.11	1.52 ± 0.24

**Table 2:** Antiproliferative activity and selectivity indices of **S10** and **1** against cancer cells and AML-12.

Cell Lines		IC_50_ Values (μM)		SI ^a^	
		S10	1	S10	1
Huh7	24 h	9.3 ± 0.28	196.0 ± 0.91	3.3	1.0
	48 h	5.9 ± 0.31	187.1 ± 1.01	5.0	1.0
Hep G2	24 h	36.3 ± 0.14	125.3 ± 2.80	0.8	1.6
	48 h	32.1 ± 1.32	121.8 ± 0.77	0.9	1.5
MDA-MB-231	24 h	34.5 ± 0.50	112.5 ± 2.20	0.9	1.8
	48 h	29.6 ± 1.43	105.1 ± 0.56	1.0	1.8
AML-12	24 h	30.3 ± 1.11	197.1 ± 0.79	-	-
	48 h	29.5 ± 1.04	186.5 ± 0.67	-	-

^a^ SI: selectivity index = IC_50_ (AML-12)/IC_50_ (cancer cells).

**Table 3:** ADME properties of **S1**–**S17**.

Compd	MW ^a^	Fraction Csp^3^	tPSA ^a^ (Å^2^)	HBD ^a^	HBA ^a^	Rotor ^a^	Log *p*	GI Absorption	CYP3A4 Inhibitor
**S1**	479.95	0.37	57.23	0	6	5	4.21	High	Yes
**S2**	513.50	0.39	57.23	0	9	6	4.67	High	Yes
**S3**	445.51	0.37	57.23	0	6	5	3.64	High	Yes
**S4**	463.50	0.37	57.23	0	7	5	4.02	High	Yes
**S5**	479.95	0.37	57.23	0	6	5	4.21	High	Yes
**S6**	463.50	0.37	57.23	0	7	5	3.87	High	Yes
**S7**	524.40	0.37	57.23	0	6	5	4.33	High	Yes
**S8**	481.49	0.37	57.23	0	8	5	4.10	High	Yes
**S9**	481.49	0.37	57.23	0	8	5	4.29	High	Yes
**S10**	513.17	0.39	57.23	0	9	6	4.64	High	Yes
**S11**	535.58	0.43	84.92	0	9	8	3.60	High	Yes
**S12**	542.39	0.37	57.23	0	7	5	4.51	High	Yes
**S13**	475.53	0.39	66.46	0	7	6	3.63	High	Yes
**S14**	490.50	0.37	103.05	0	8	6	2.88	High	Yes
**S15**	505.56	0.41	75.69	0	8	7	3.47	High	Yes
**S16**	542.39	0.37	57.23	0	7	5	4.56	High	Yes
**S17**	481.49	0.37	57.23	0	8	5	4.35	High	Yes

^a^ MW (molecular weight), tPSA (topological polar surface area), HBD (number of H-bond donors), HBA (number of H-bond acceptors), Rotor (number of rotatable bonds).

## Data Availability

No new data were created or analyzed in this study. Data sharing is not applicable to this article.

## Supplementary Materials

The following supporting information can be downloaded at: https://www.mdpi.com/article/10.3390/molecules31193478/s1, Figures S1–S56: Network pharmacology analysis of **S10** in HCC; ^1^H, ^13^C NMR spectra and HR-ESI-MS of compounds **S1**–**S17**.
