# Supplementary material for: Design, Synthesis, and Evaluation of Novel Cephalotaxine-Cinnamic Acid Derivatives Against Hepatocellular Carcinoma
Source: Molecules. 2026 Sep 29;31(19):3478. doi: 10.3390/molecules31193478 (PMC13666737; doi:10.3390/molecules31193478)
Supplement: Supplementary file 1 [file molecules-31-03478-s001.zip › molecules-4540906-supplementary.pdf]

SUPPLEMENTARY MATERIALS

Design, Synthesis, and Evaluation of Novel Cephalotaxine–Cinnamic Acid Derivatives Against Hepatocellular Carcinoma

Wenxing Li[1,2,†], Yiming Qian[1,2,†], Hao Wang[3], Yang Hui[1,2,*], Haofu Dai[3,*], Wenhao Chen[1,2,*]

1 Key Laboratory of Tropical Medicinal Resource Chemistry of Ministry of Education, College of Chemistry and Chemical Engineering, Hainan Normal University, Haikou, 571158, China; 202311070300001@hainnu.edu.cn (W.L.); 202212070300016@hainnu.edu.cn (Y.Q.)

2 Key Laboratory of Tropical Medicinal Plant Chemistry of Hainan Province, College of Chemistry and Chemical Engineering, Hainan Normal University, Haikou, 571158, China;

3 Key Laboratory of Research and Development of Natural Product from Li Folk Medicine of Hainan Province, Institute of Tropical Bioscience and Biotechnology, Chinese Academy of Tropical Agricultural Sciences, Haikou 572829, China; wanghao@itbb.org.cn (H.W.);

* Correspondence: 070103@hainnu.edu.cn (W.C.); daihaofu@itbb.org.cn (H.D.); 070066@hainnu.edu.cn (Y.H.)

† These authors contributed equally to this work.

Abstract: Cephalotaxine (**1**) is a parent compound within the cephalotaxine alkaloids class mainly derived from Cephalotaxus plants. In this study, by coupling **1** with cinnamic acid, 17 novel cephalotaxine-cinnamic acid derivatives (**S1**-**S17**) were designed and synthesized to enhance their anti-tumor efficacy. Among them, **S10** shown the strongest cytotoxic activity against Huh7 cells, with the IC_{50} values of 9.3 and 5.9 μM after 24 h and 48 h, respectively, which were 21 and 32 times more potent than those of **1**. In addition, compared with those against AML-12 cells, the proliferation inhibition rates of **S10** against Huh7 cells were 5.0-fold lower, indicating certain therapeutic window. Network pharmacology screening results indicate that **S10** may exert regulatory effects closely related to cell migration and apoptosis through pathways such as PI3K/AKT. Molecular docking showed that **S10** could effectively bind to the key targets PI3K and AKT. And the western blotting experiment results confirm that **S10** could downregulate the expression of p-PI3K and p-AKT at the protein level while keeping total PI3K and AKT unchanged. Notably, **S10** may regulate liver cancer cells by inhibiting the PI3K/AKT signaling pathway. Mechanistically, **S10** induces S cycle arrest, reduces mitochondrial membrane potential, induces apoptosis, and inhibits migration and invasion. Overall, we reported **S10** as a promising lead compound for the treatment of liver cancer.

Keywords: Cephalotaxus hainanensis Li; Cephalotaxine; Liver cancer; PI3K/AKT pathway

Contents

Network pharmacology analysis of **S10** in HCC................................3
^{1}H- /^{13}C-NMR, HR-ESI-MS spectrum of compound **1**..................................4
^{1}H- /^{13}C-NMR, HR-ESI-MS spectrum of compound **S1**..................................5
^{1}H- /^{13}C-NMR, HR-ESI-MS spectrum of compound **S2**..................................7
^{1}H- /^{13}C-NMR, HR-ESI-MS spectrum of compound **S3**..................................8
^{1}H- /^{13}C-NMR, HR-ESI-MS spectrum of compound **S4**................................10
^{1}H- /^{13}C-NMR, HR-ESI-MS spectrum of compound **S5**................................11
^{1}H- /^{13}C-NMR, HR-ESI-MS spectrum of compound **S6**................................13
^{1}H- /^{13}C-NMR, HR-ESI-MS spectrum of compound **S7**................................14
^{1}H- /^{13}C-NMR, HR-ESI-MS spectrum of compound **S8**................................16
^{1}H- /^{13}C-NMR, HR-ESI-MS spectrum of compound **S9**................................17
^{1}H- /^{13}C-NMR, HR-ESI-MS spectrum of compound **S10** 19
^{1}H- /^{13}C-NMR, HR-ESI-MS spectrum of compound **S11**................................20
^{1}H- /^{13}C-NMR, HR-ESI-MS spectrum of compound **S12**................................22
^{1}H- /^{13}C-NMR, HR-ESI-MS spectrum of compound **S13**................................23
^{1}H- /^{13}C-NMR, HR-ESI-MS spectrum of compound **S14**................................25
^{1}H- /^{13}C-NMR, HR-ESI-MS spectrum of compound **S15**................................26
^{1}H- /^{13}C-NMR, HR-ESI-MS spectrum of compound **S16**................................28
^{1}H- /^{13}C-NMR, HR-ESI-MS spectrum of compound **S17**................................29

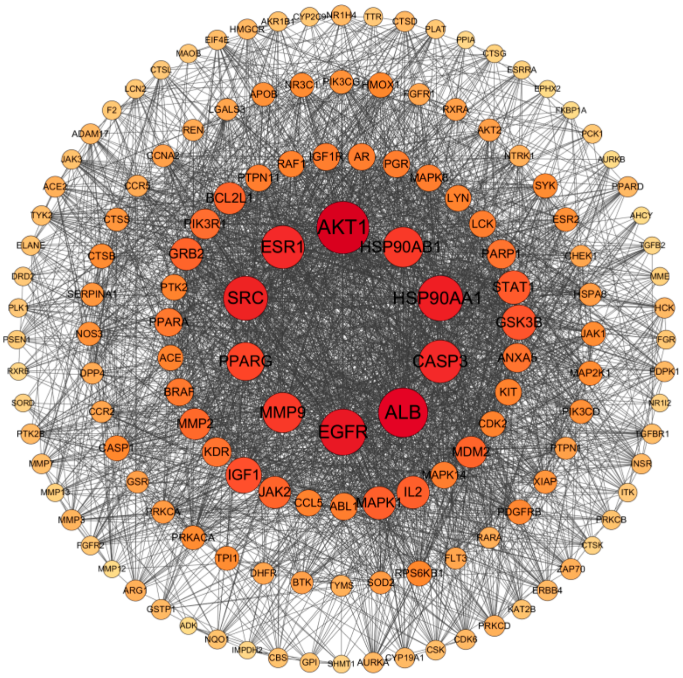

Figure S1. Network pharmacology analysis of **S10** in HCC: PPI network from the STRING database to Cytoscape 3.9.2.

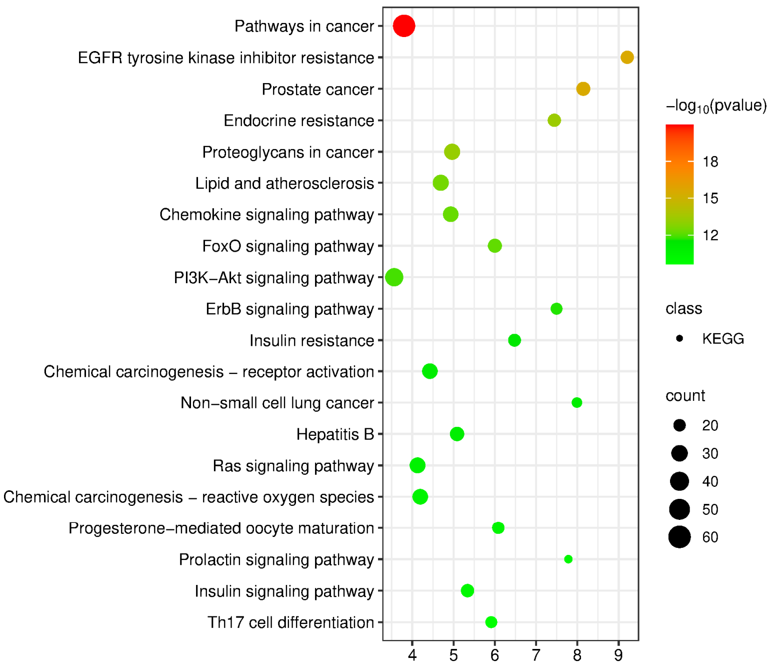

Figure S2. Network pharmacology analysis of **S10** in HCC: Bubble plot KEGG analysis.

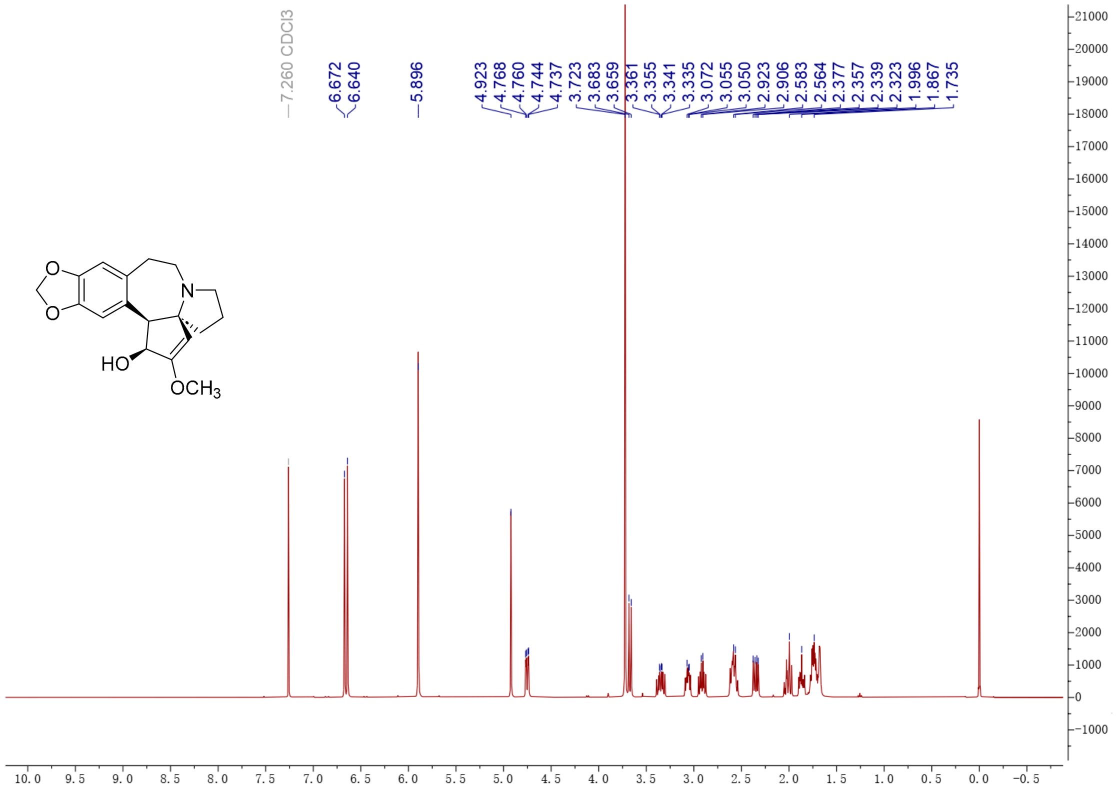

Figure S3. ^{1}H-NMR spectrum of **1**.

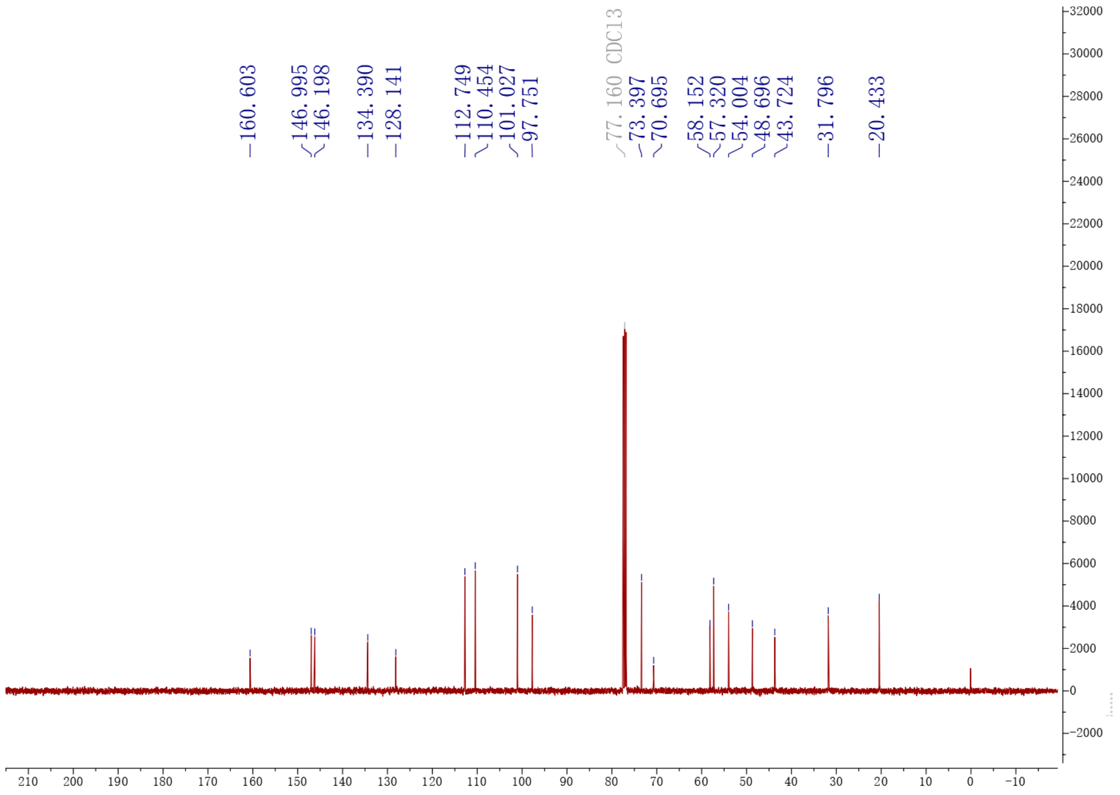

Figure S4. ^{13}C-NMR spectrum of **1**.

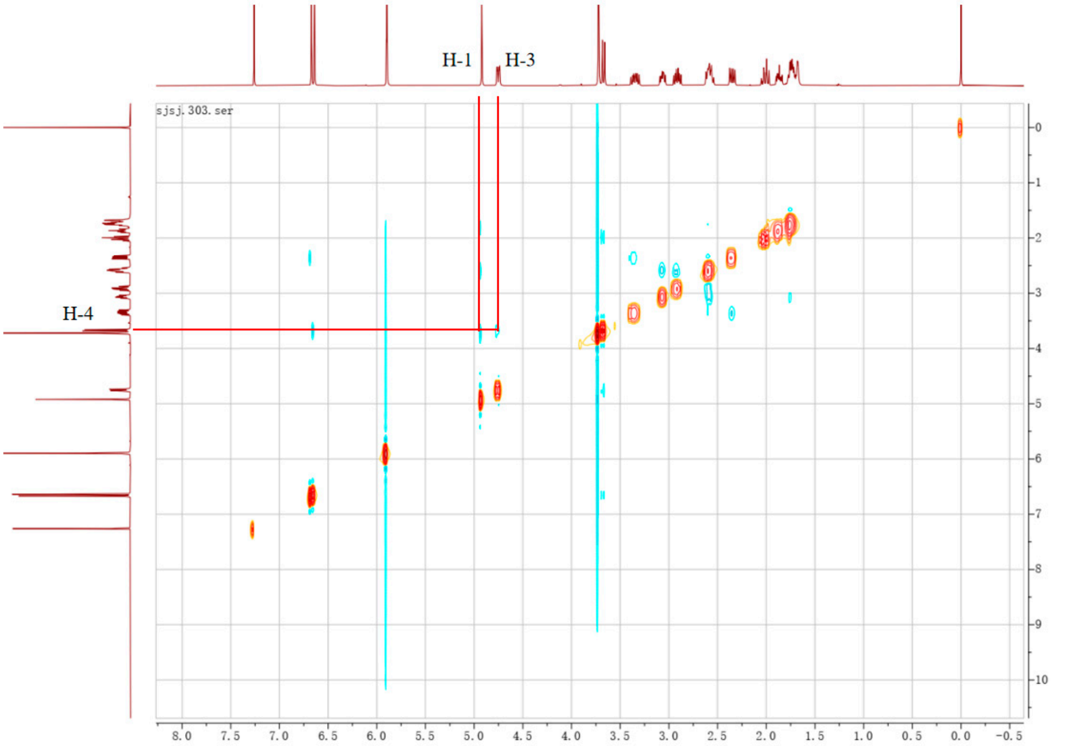

Figure S5. NOESY spectrum of **1**.

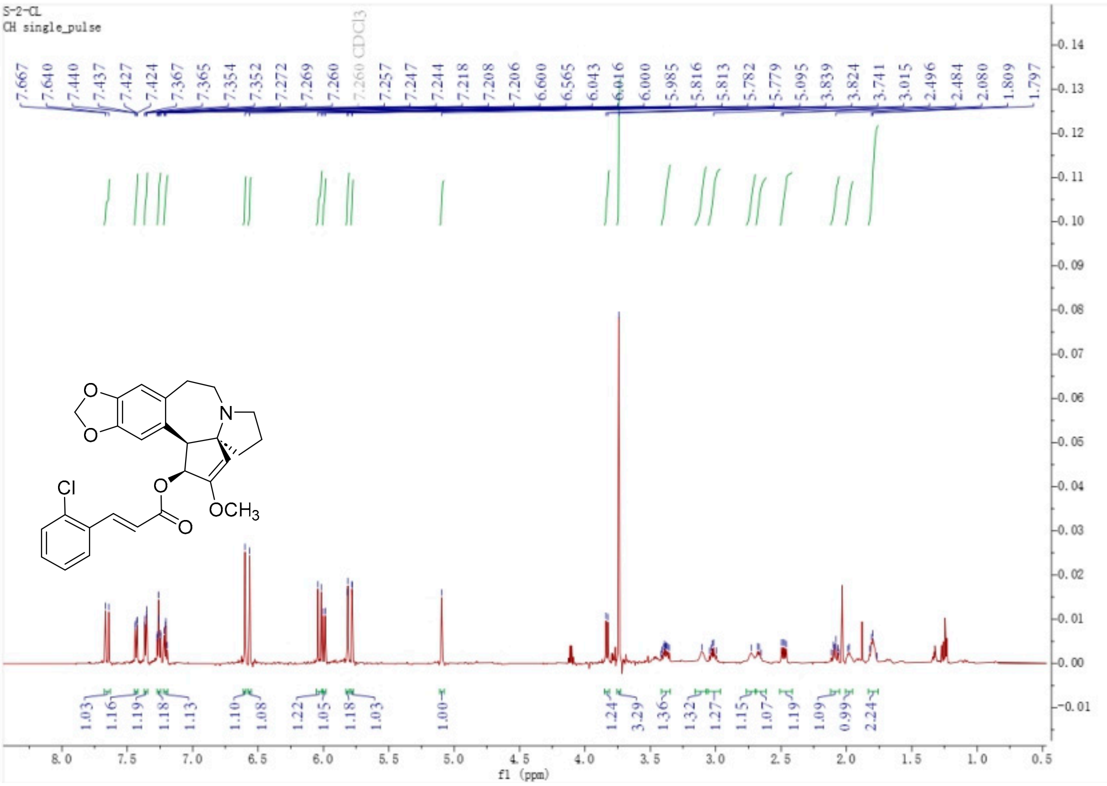

Figure S6. ^{1}H-NMR spectrum of **S1**.

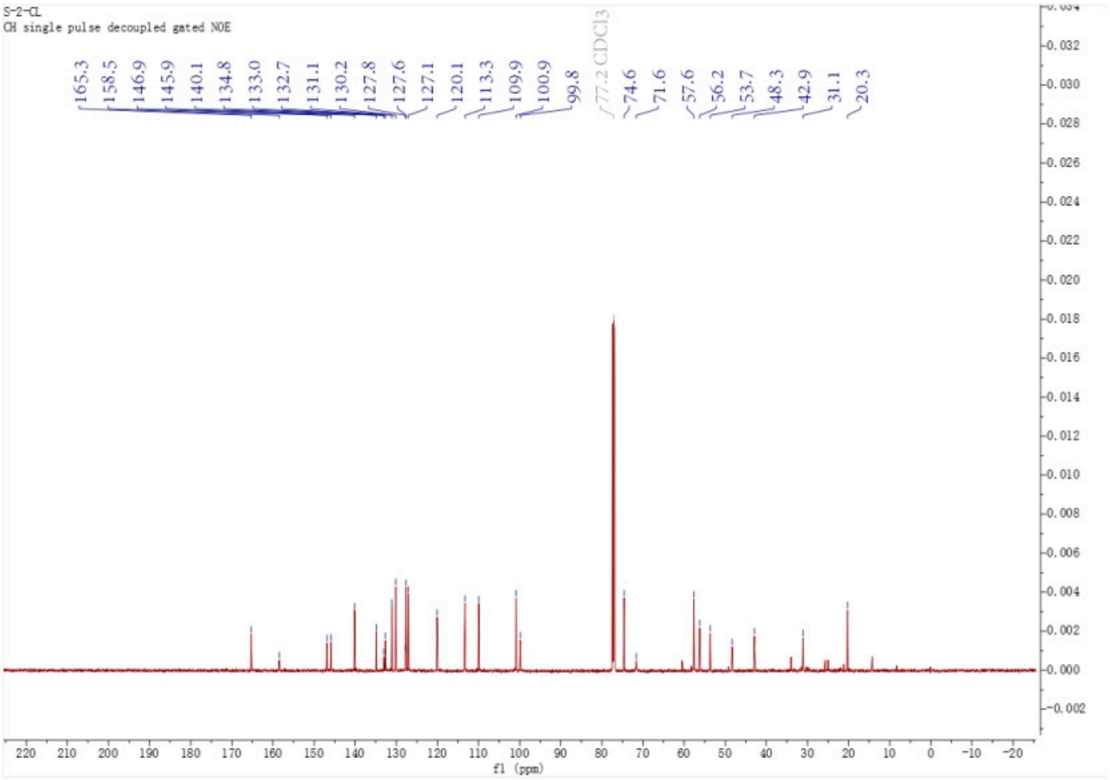

Figure S7. ^{13}C-NMR spectrum of **S1**.

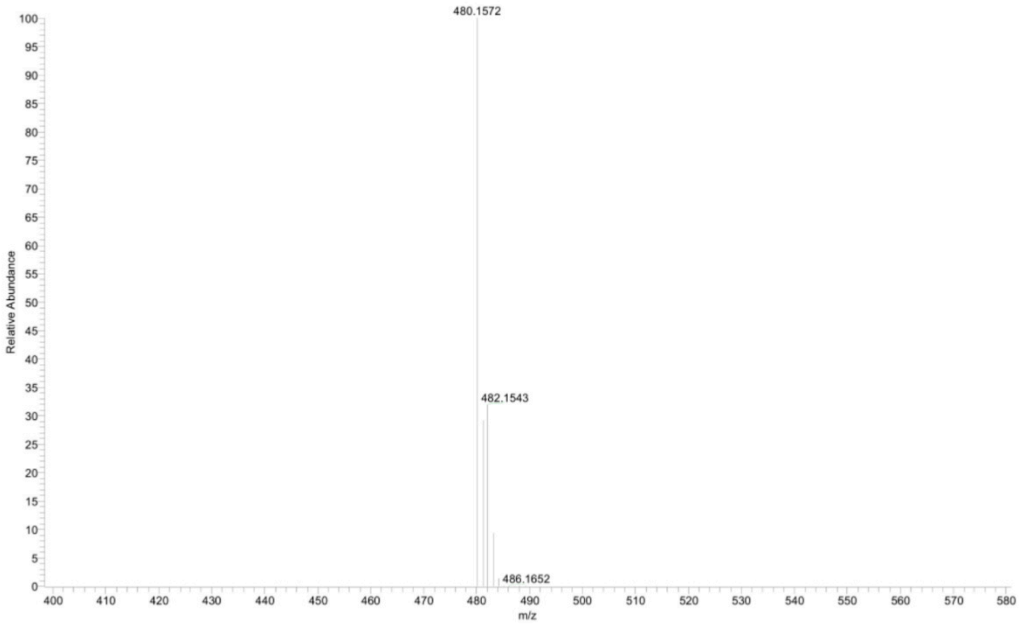

Figure S8. HR-ESI-MS spectrum of **S1**.

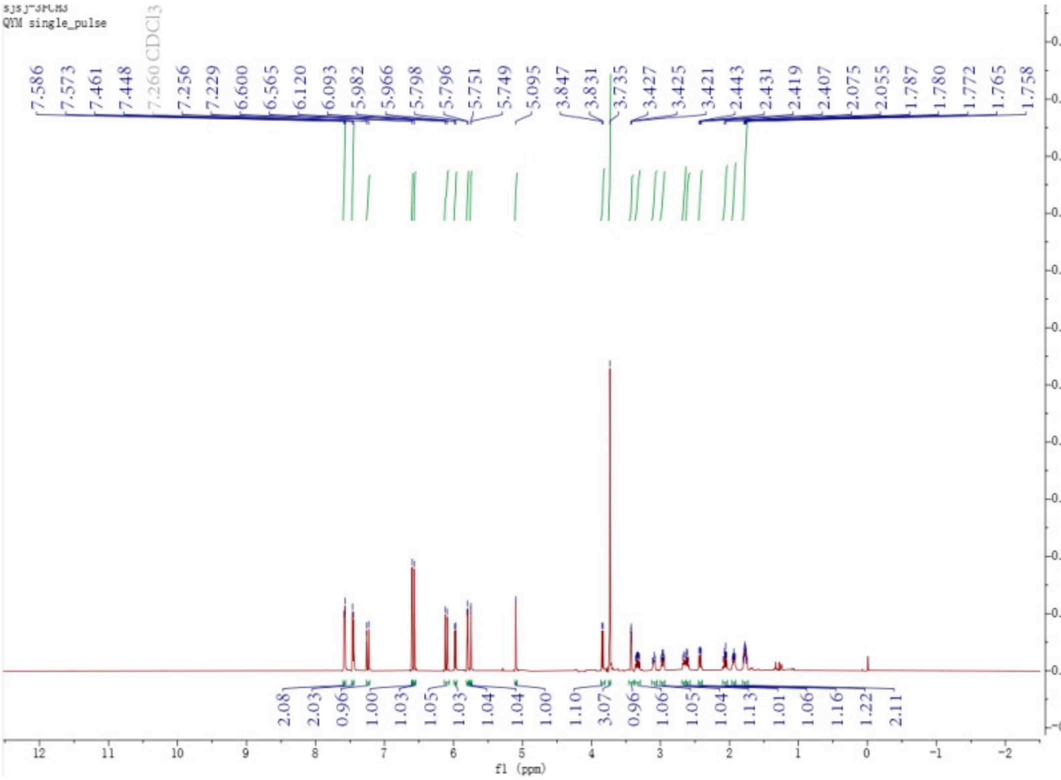

Figure S9. ^{1}H-NMR spectrum of **S2**.

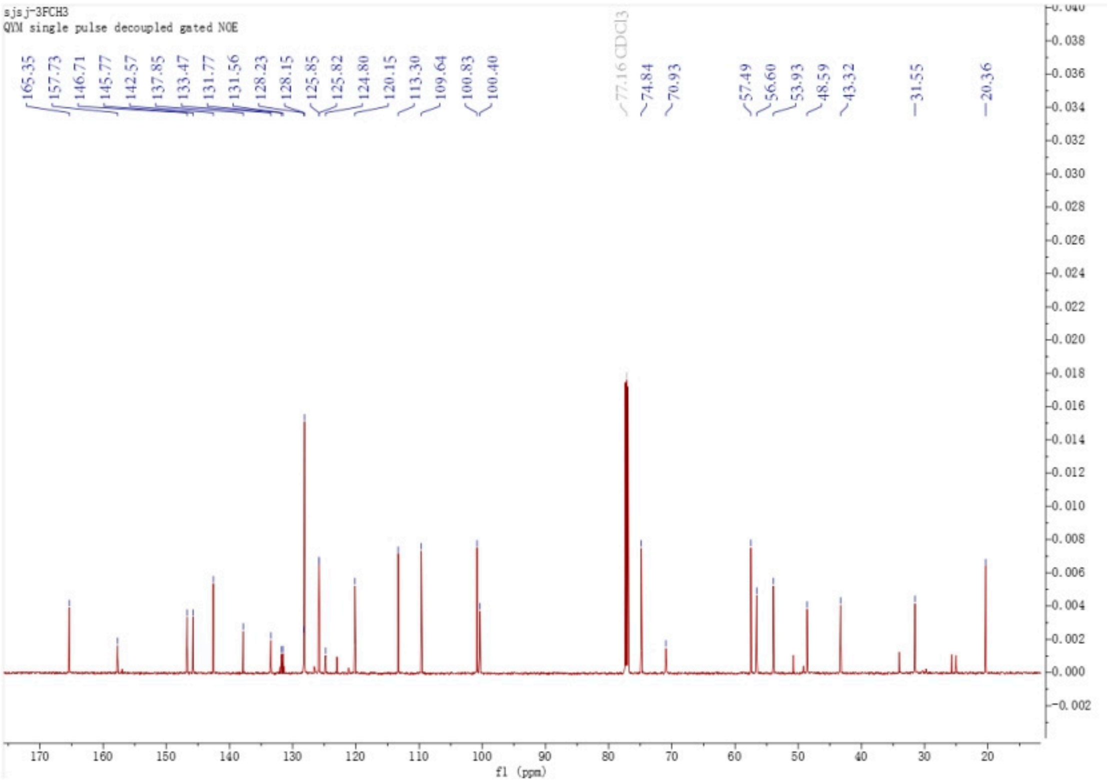

Figure S10. ^{13}C-NMR spectrum of **S2**.

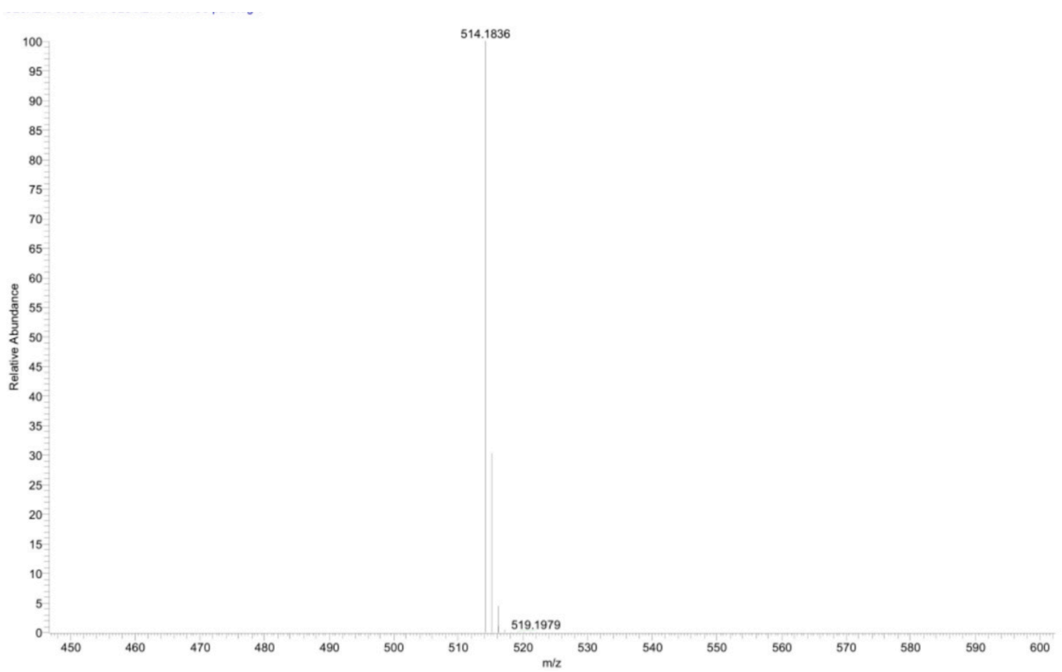

Figure S11. HR-ESI-MS spectrum of **S2**.

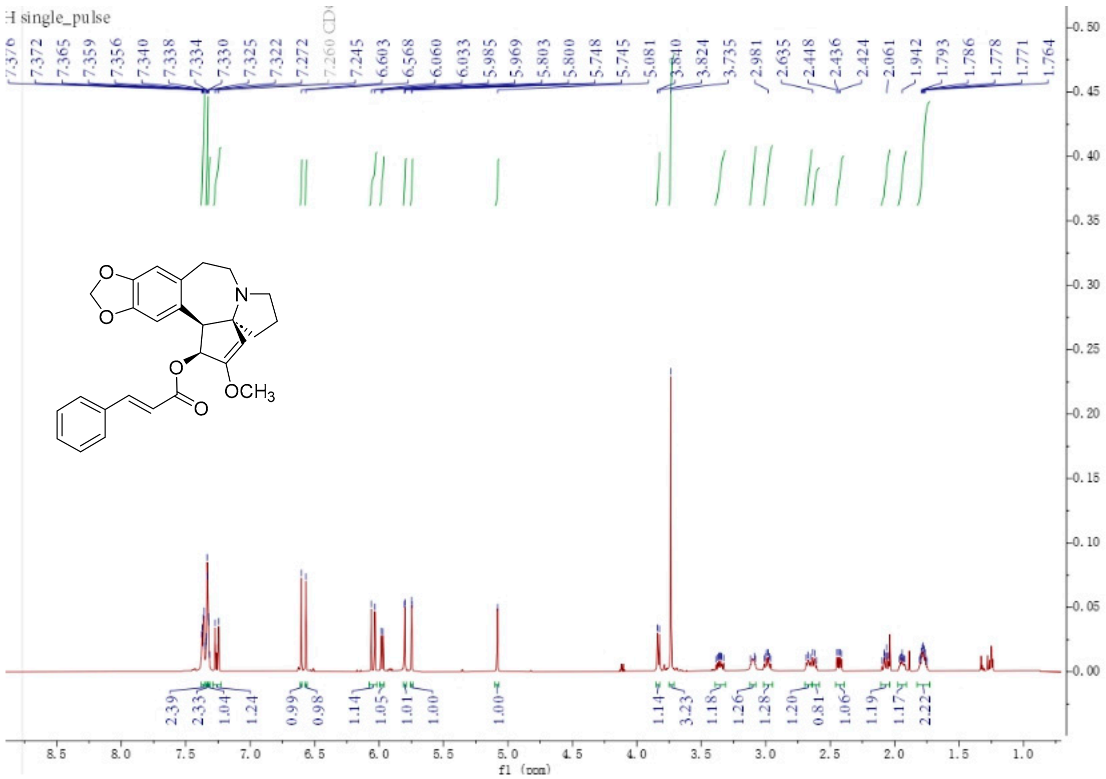

Figure S12. ^{1}H-NMR spectrum of **S3**.

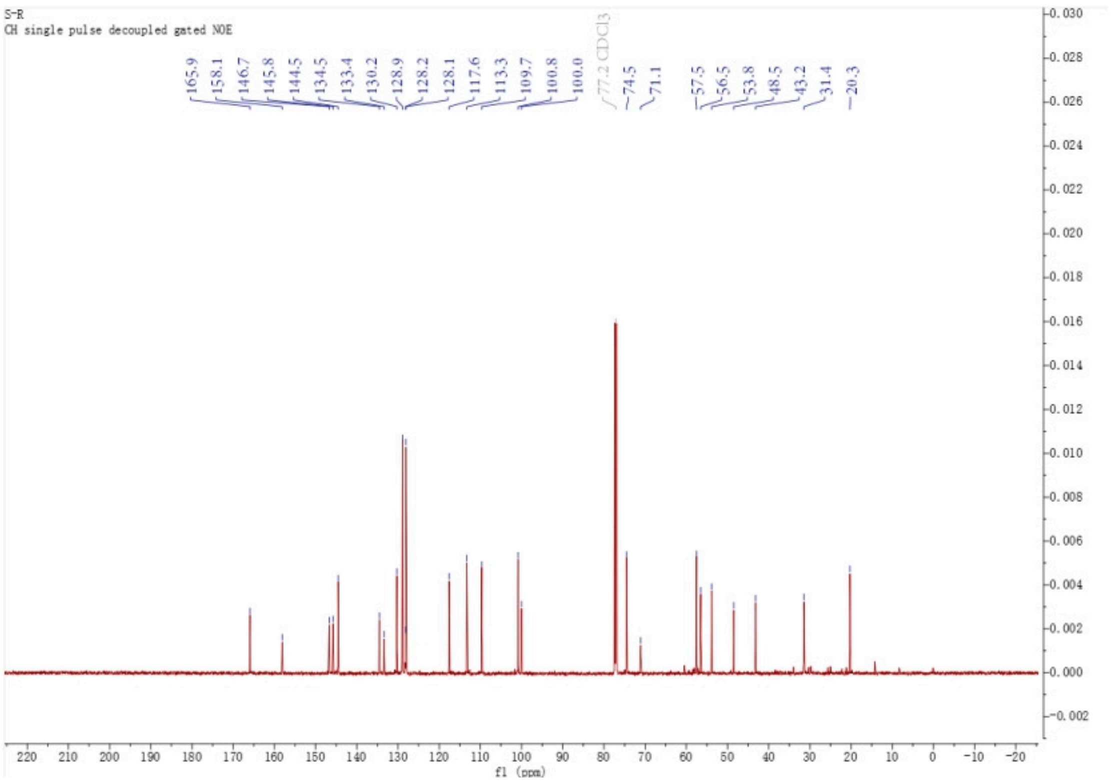

Figure S13. ^{13}C-NMR spectrum of **S3**.

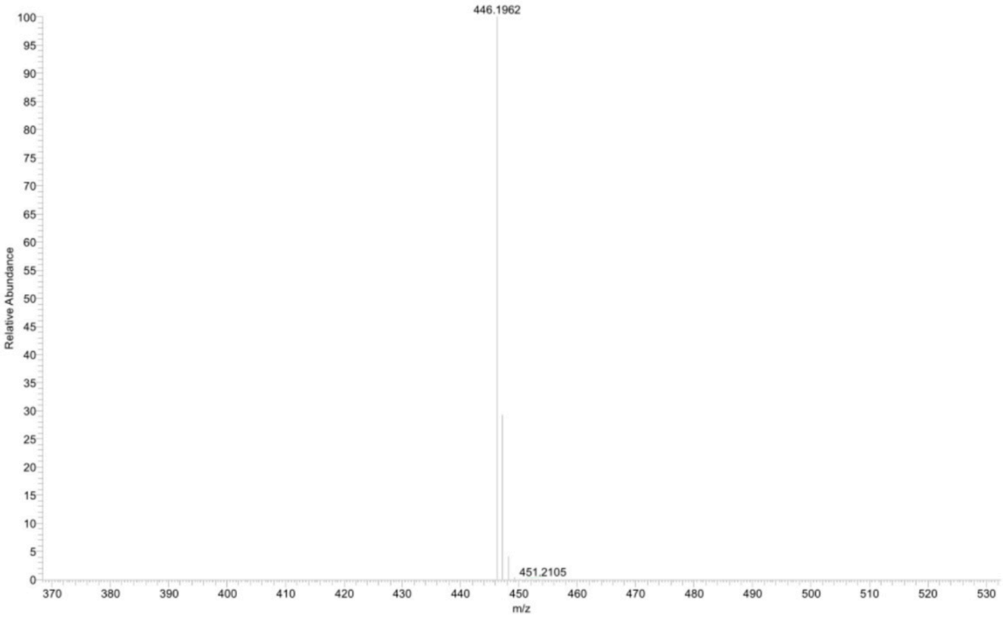

Figure S14. HR-ESI-MS spectrum of **S3**.

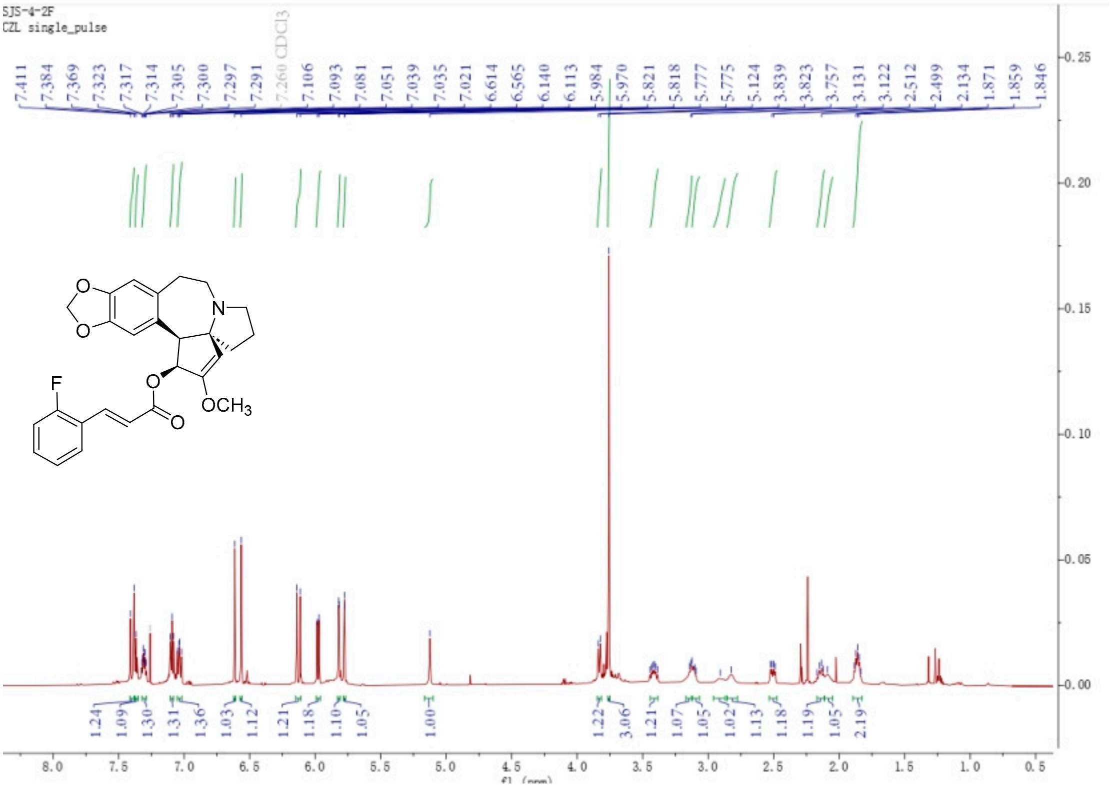

Figure S15. ^{1}H-NMR spectrum of **S4**.

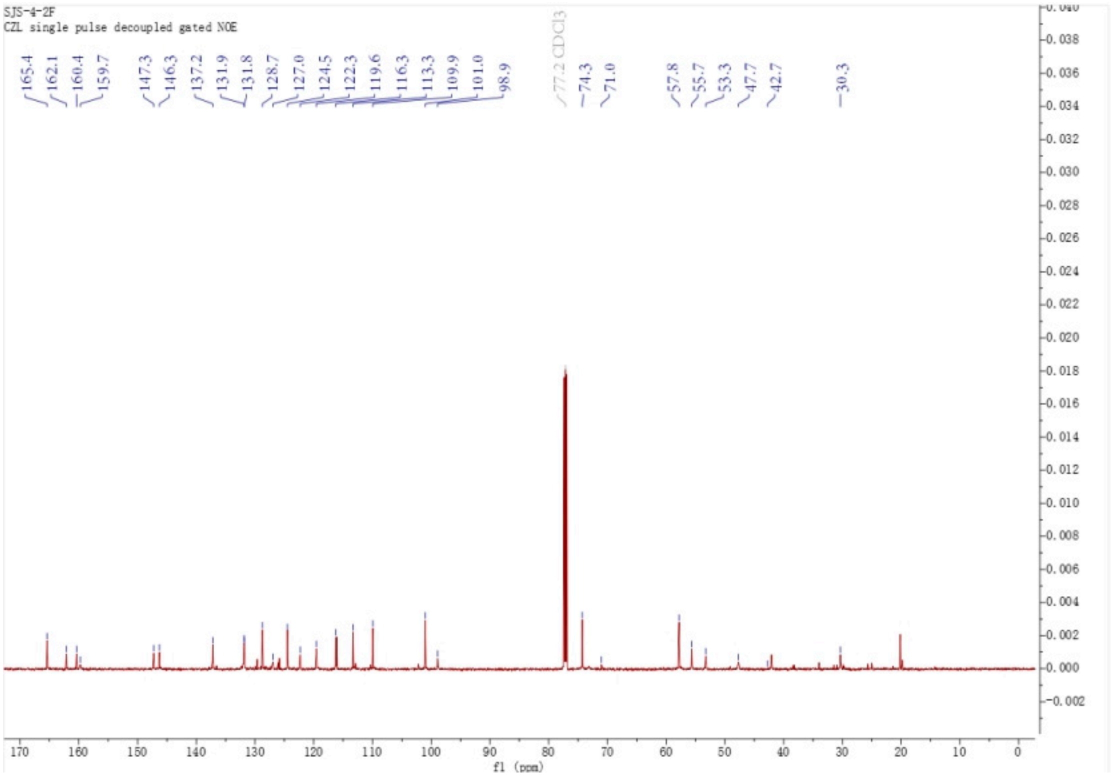

Figure S16. ^{13}C-NMR spectrum of **S4**.

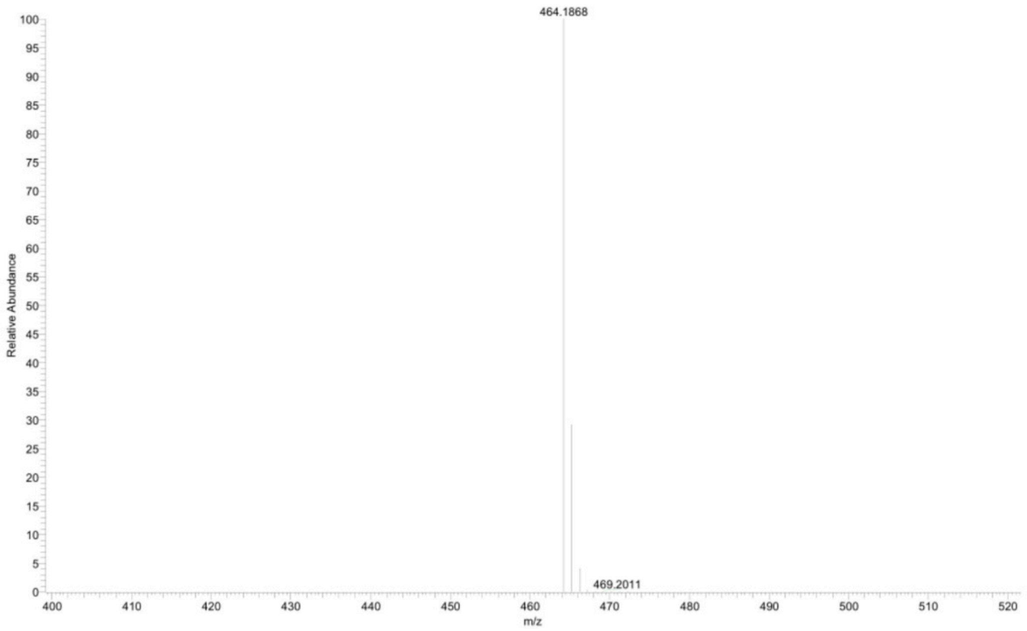

Figure S17. HR-ESI-MS spectrum of **S4**.

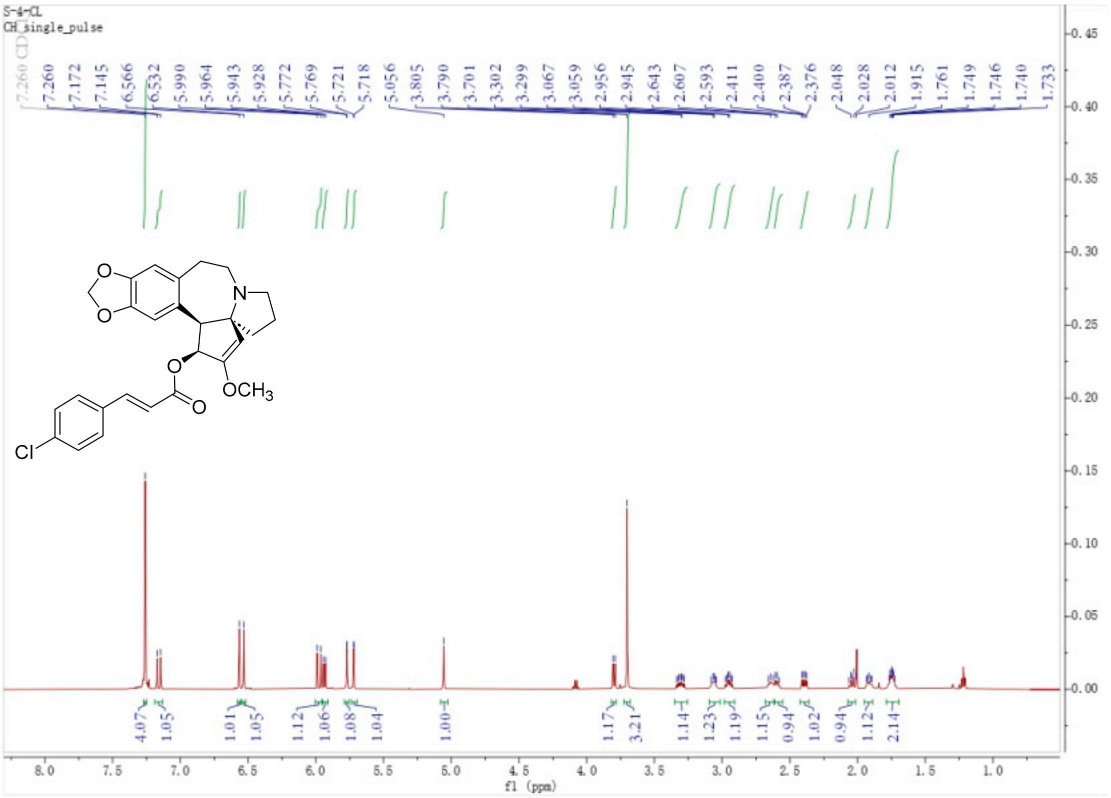

Figure S18. ^{1}H-NMR spectrum of **S5**.

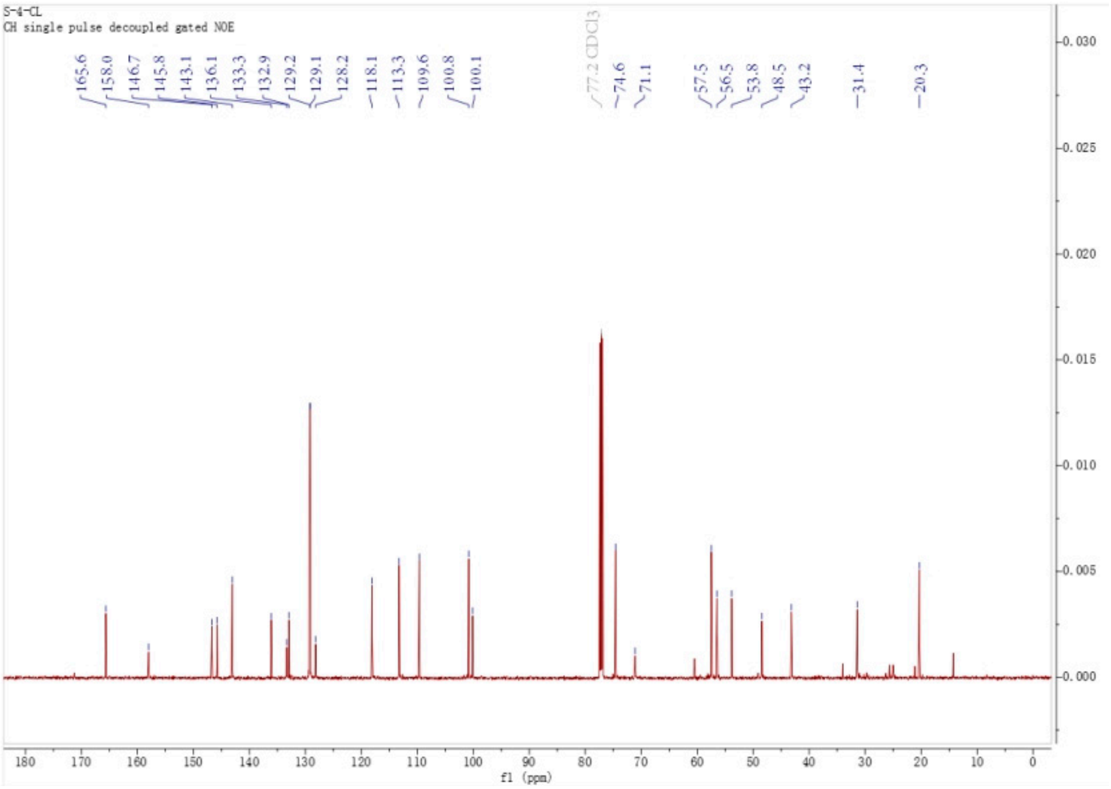

Figure S19. ^{13}C-NMR spectrum of **S5**.

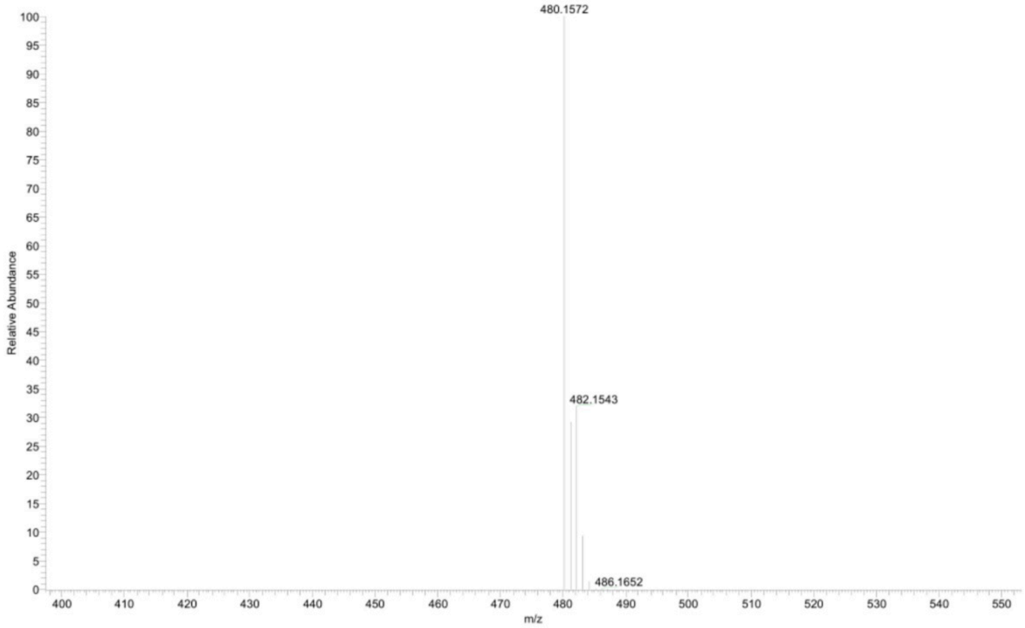

Figure S20. HR-ESI-MS spectrum of **S5**.

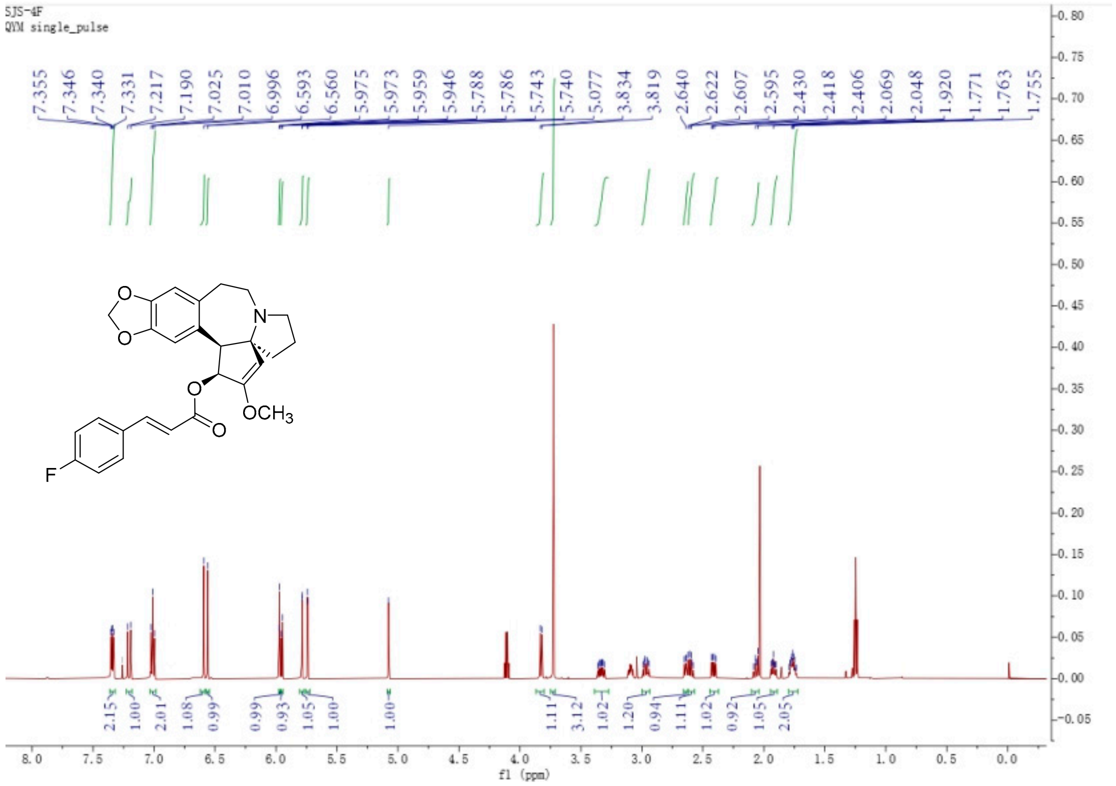

Figure S21. ^{1}H-NMR spectrum of **S6**.

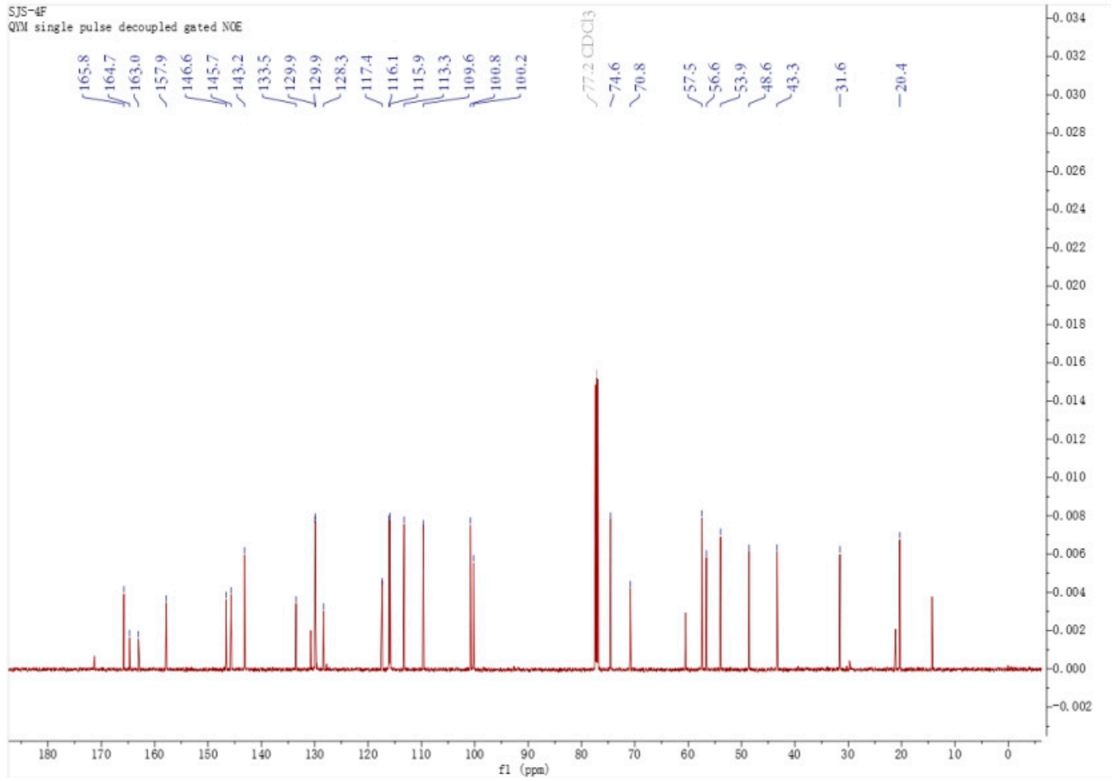

Figure S22. ^{13}C-NMR spectrum of **S6**.

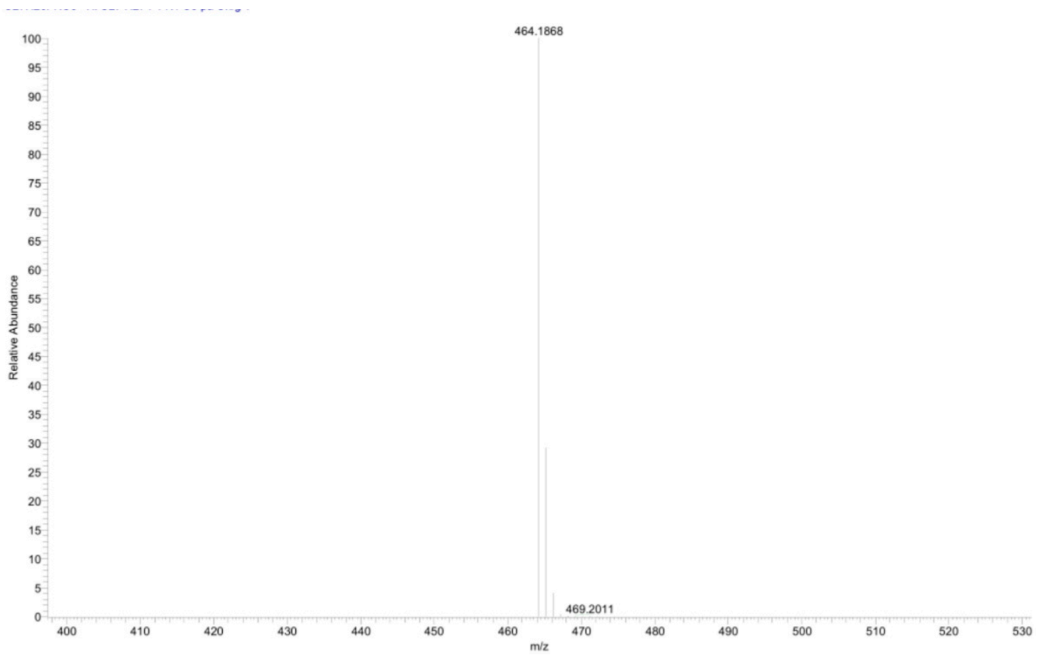

Figure S23. HR-ESI-MS spectrum of **S6**.

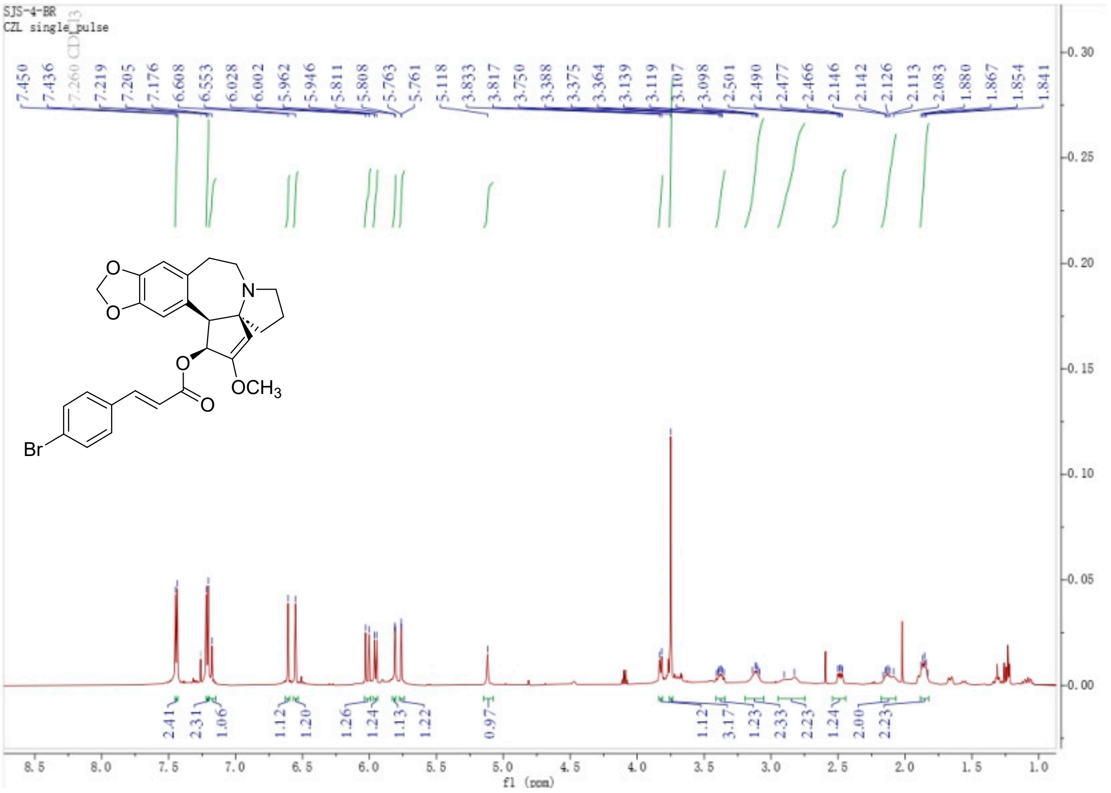

Figure S24. ^{1}H-NMR spectrum of **S7**.

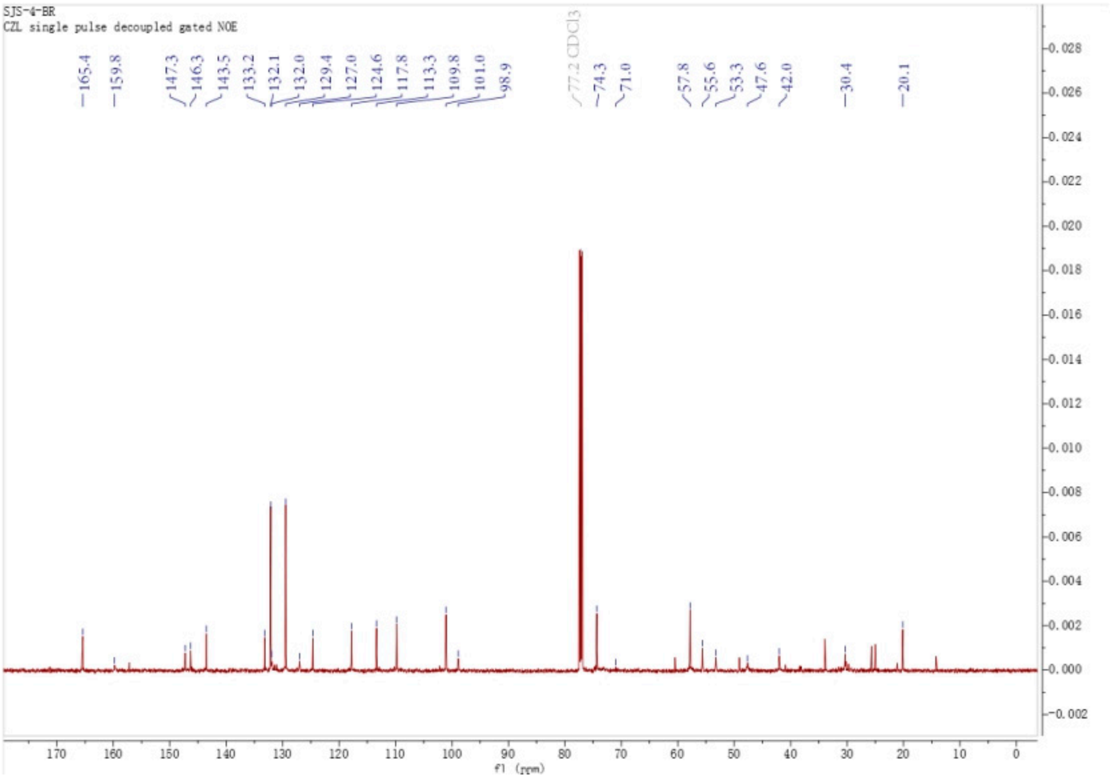

Figure S25. ^{13}C-NMR spectrum of **S7**.

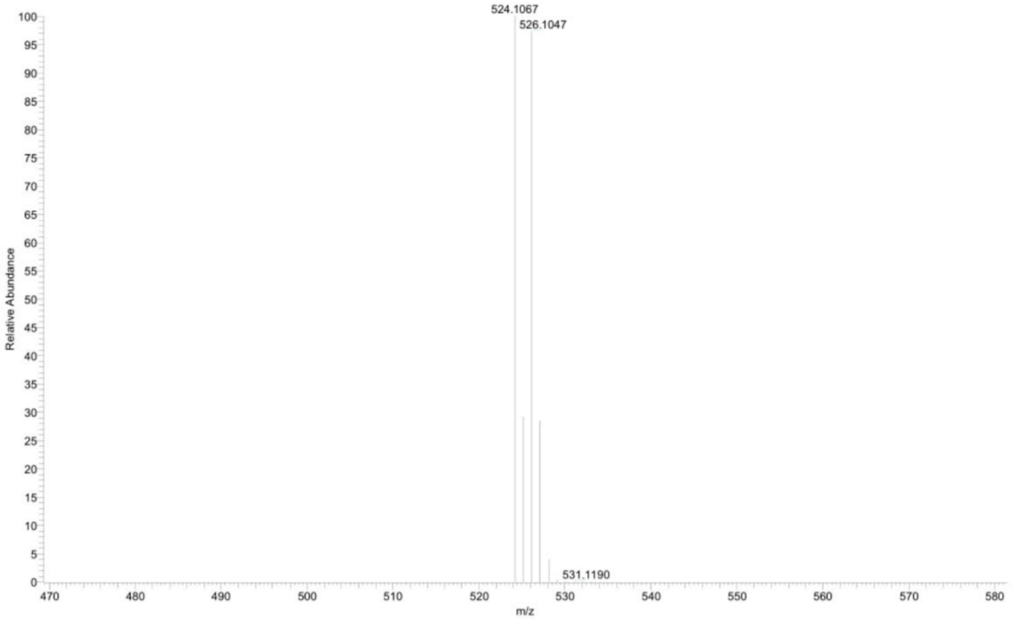

Figure S26. HR-ESI-MS spectrum of **S7**.

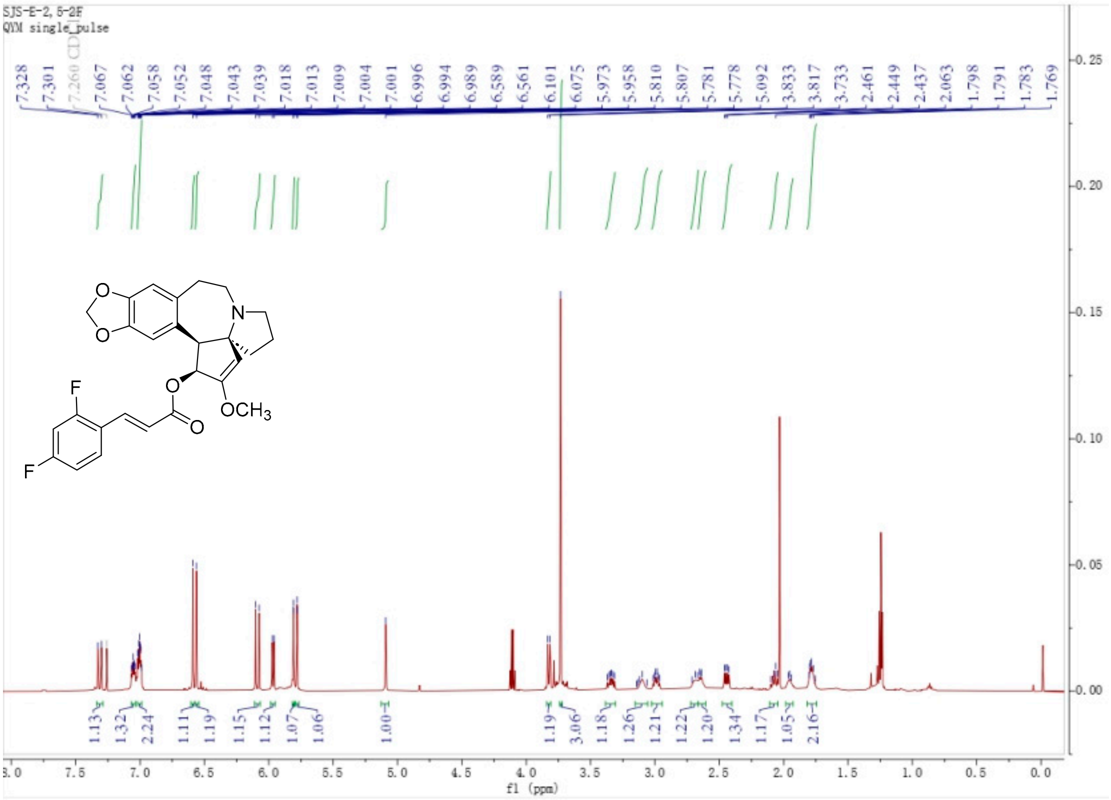

Figure S27. ^{1}H-NMR spectrum of **S8**.

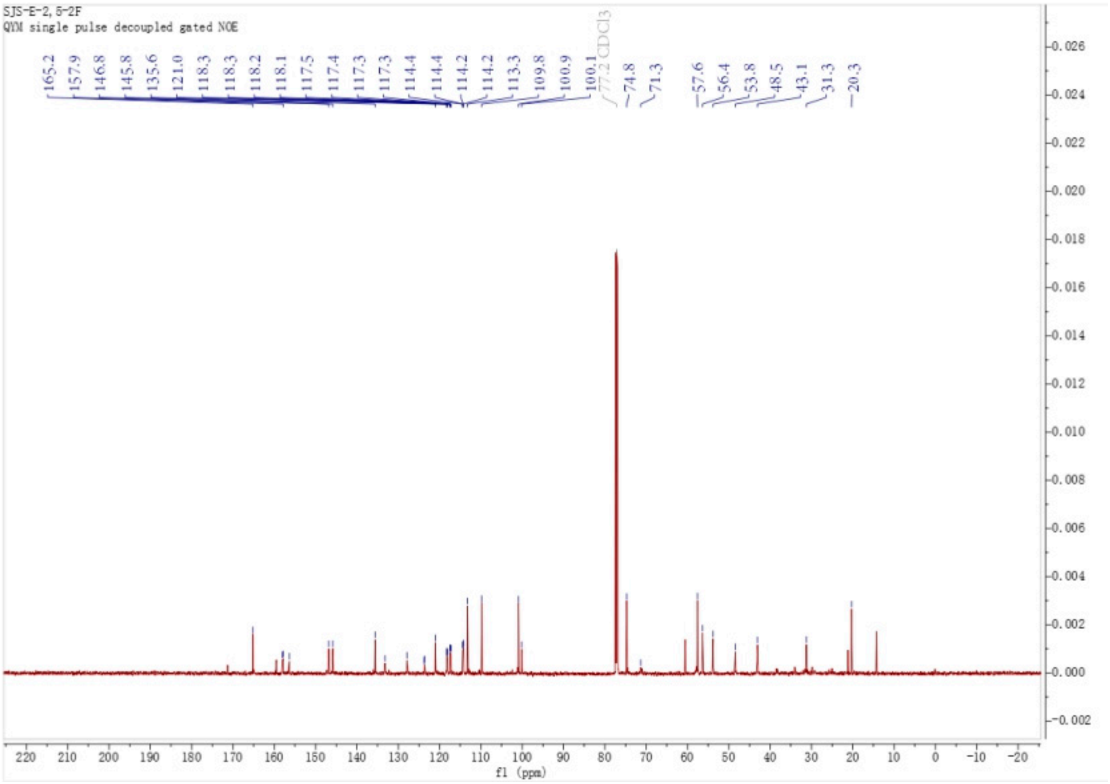

Figure S28. ^{13}C-NMR spectrum of **S8**.

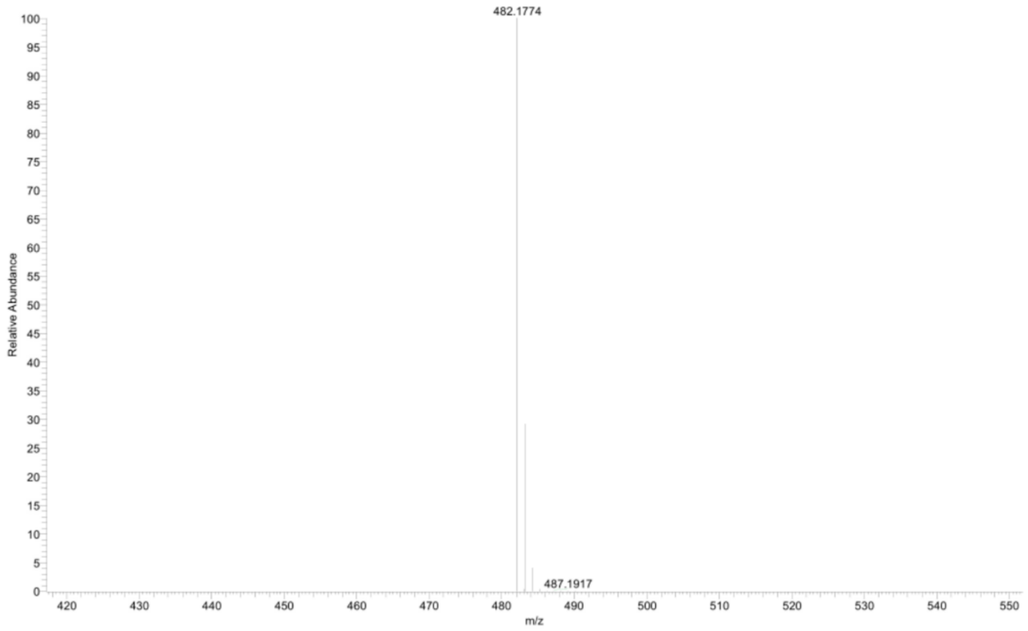

Figure S29. HR-ESI-MS spectrum of **S8**.

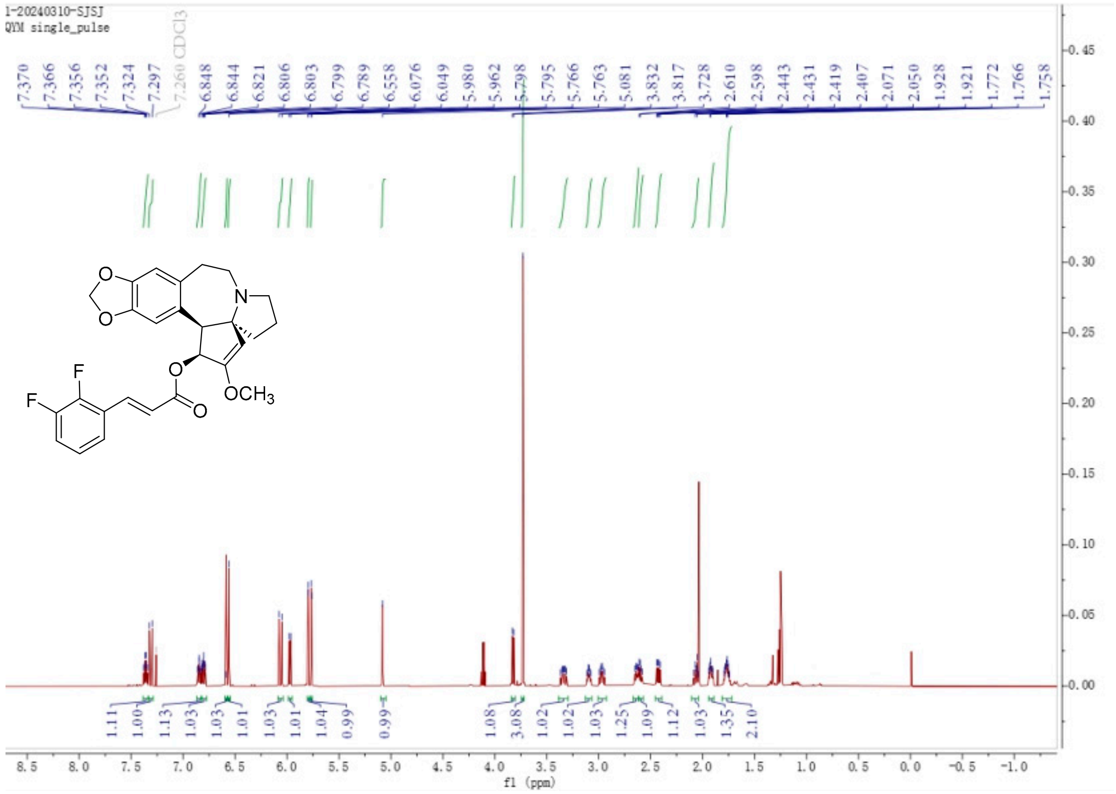

Figure S30. ^{1}H-NMR spectrum of **S9**.

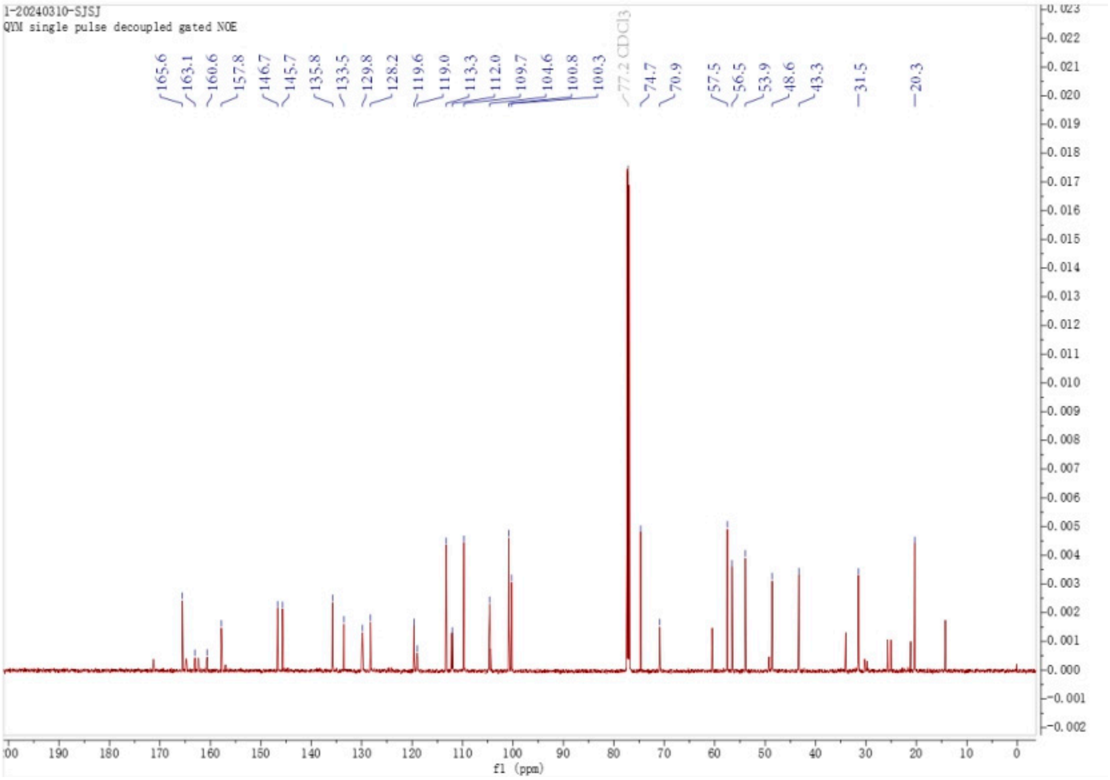

Figure S31. ^{13}C-NMR spectrum of **S9**.

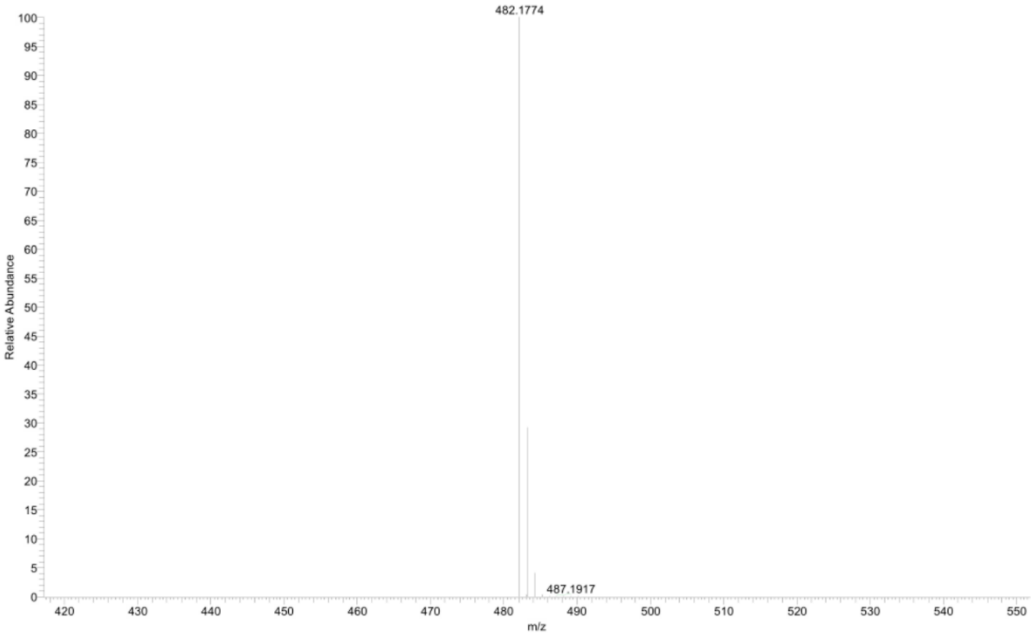

Figure S32. HR-ESI-MS spectrum of **S9**.

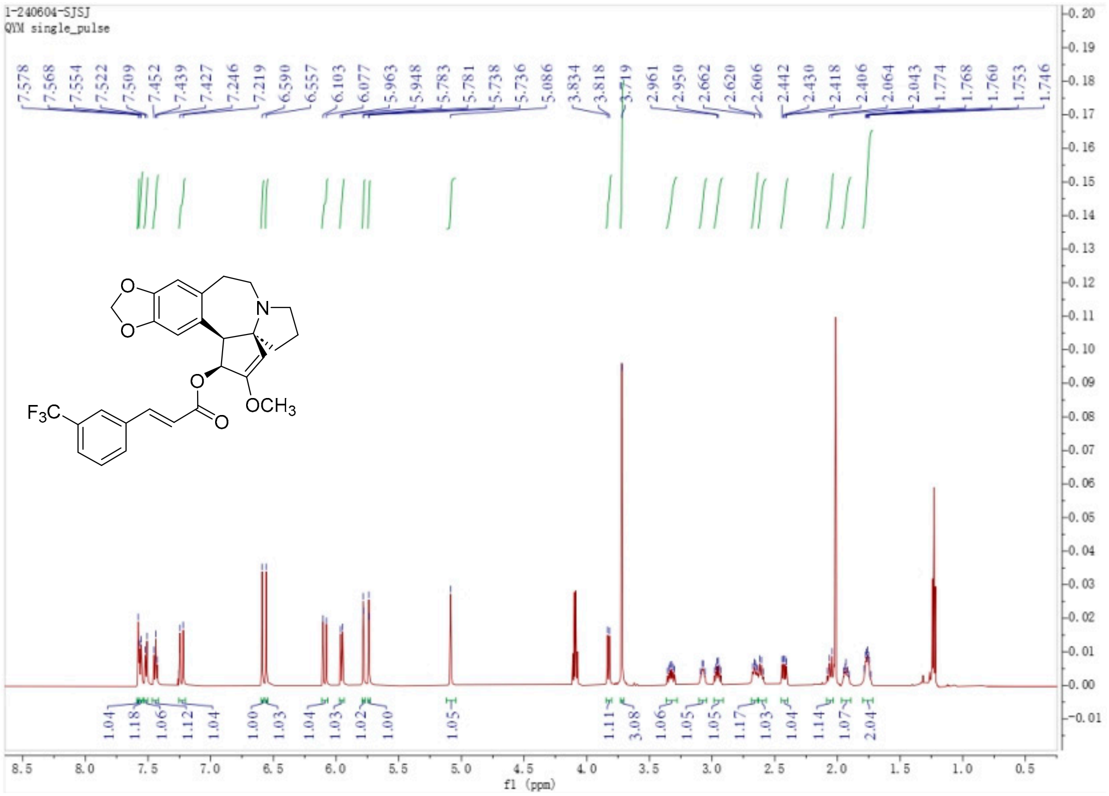

Figure S33. ^{1}H-NMR spectrum of **S10**.

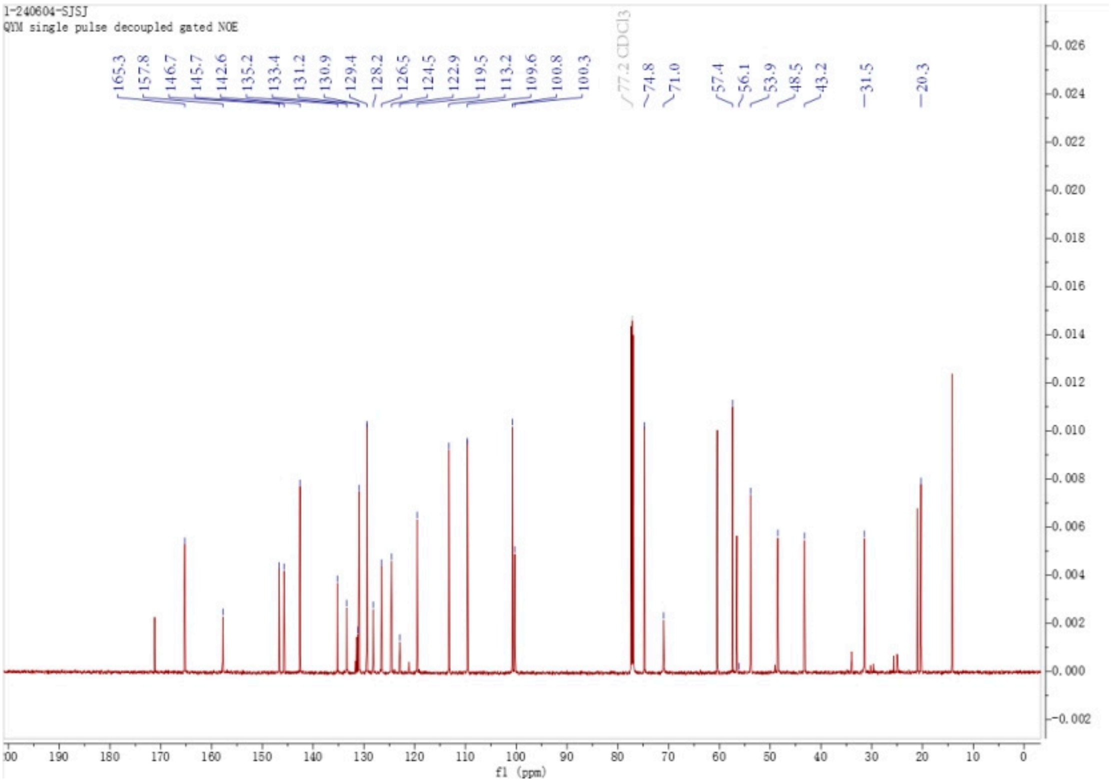

Figure S34. ^{13}C-NMR spectrum of **S10**.

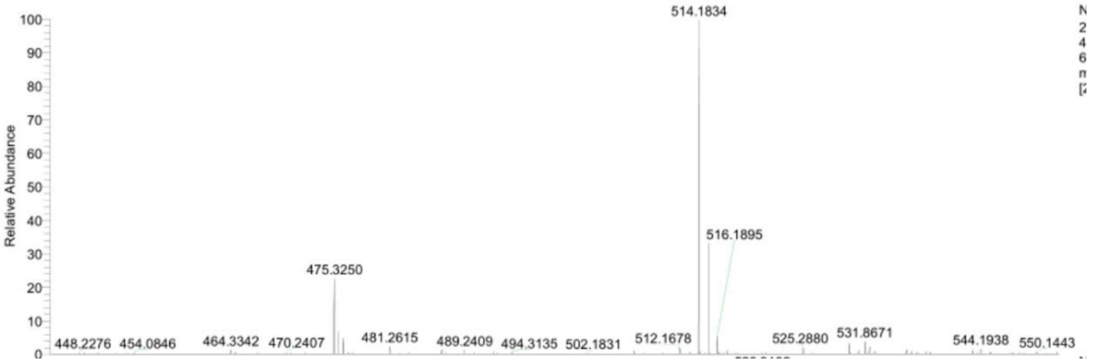

Figure S35. HR-ESI-MS spectrum of **S10**.

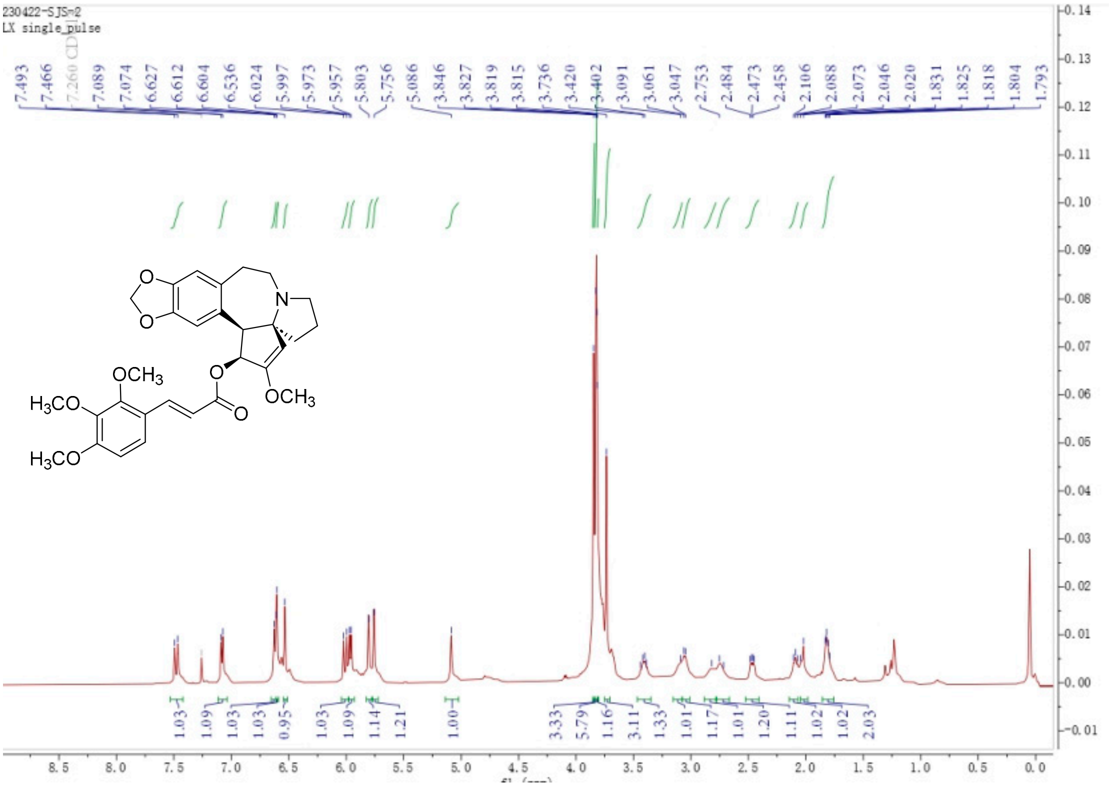

Figure S36. ^{1}H-NMR spectrum of **S11**.

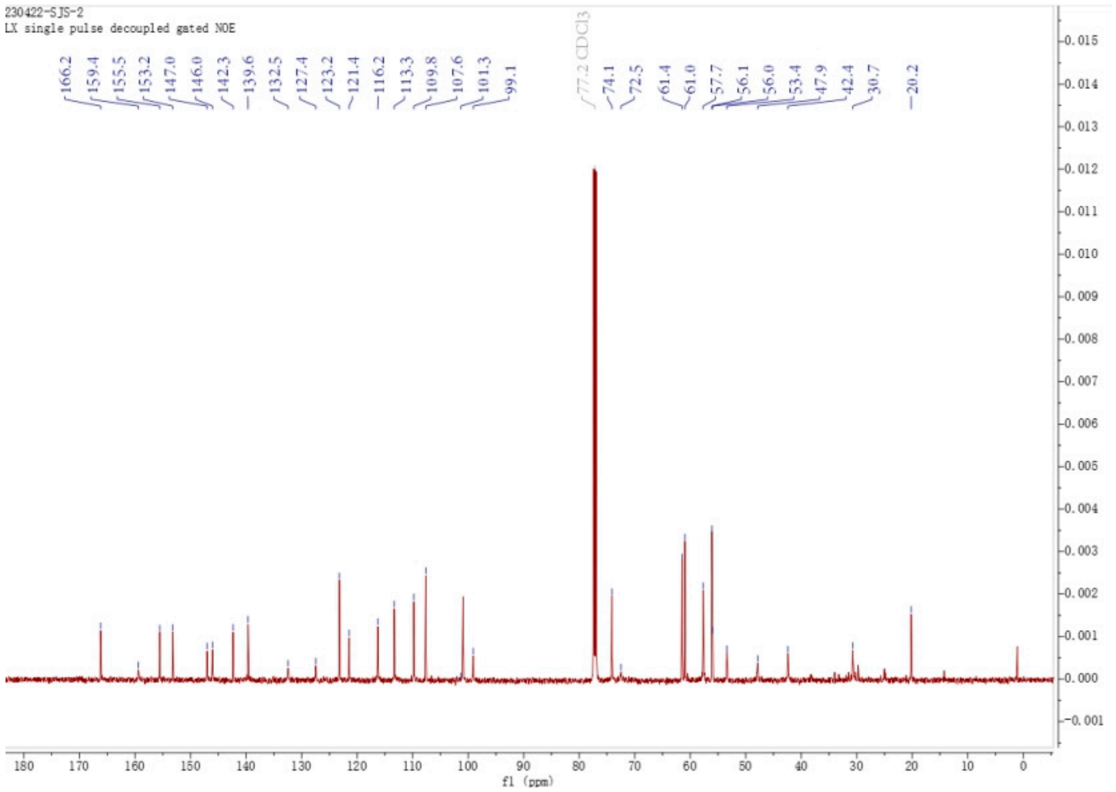

Figure S37. ^{13}C-NMR spectrum of **S11**.

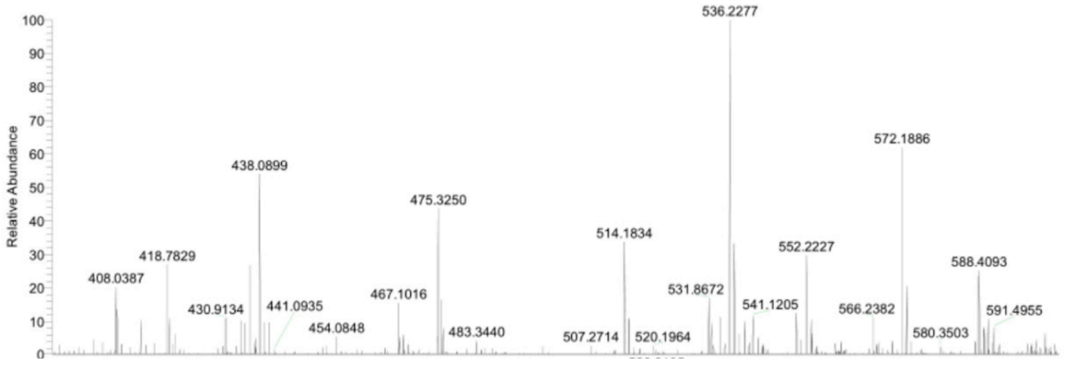

Figure S38. HR-ESI-MS spectrum of **S11**.

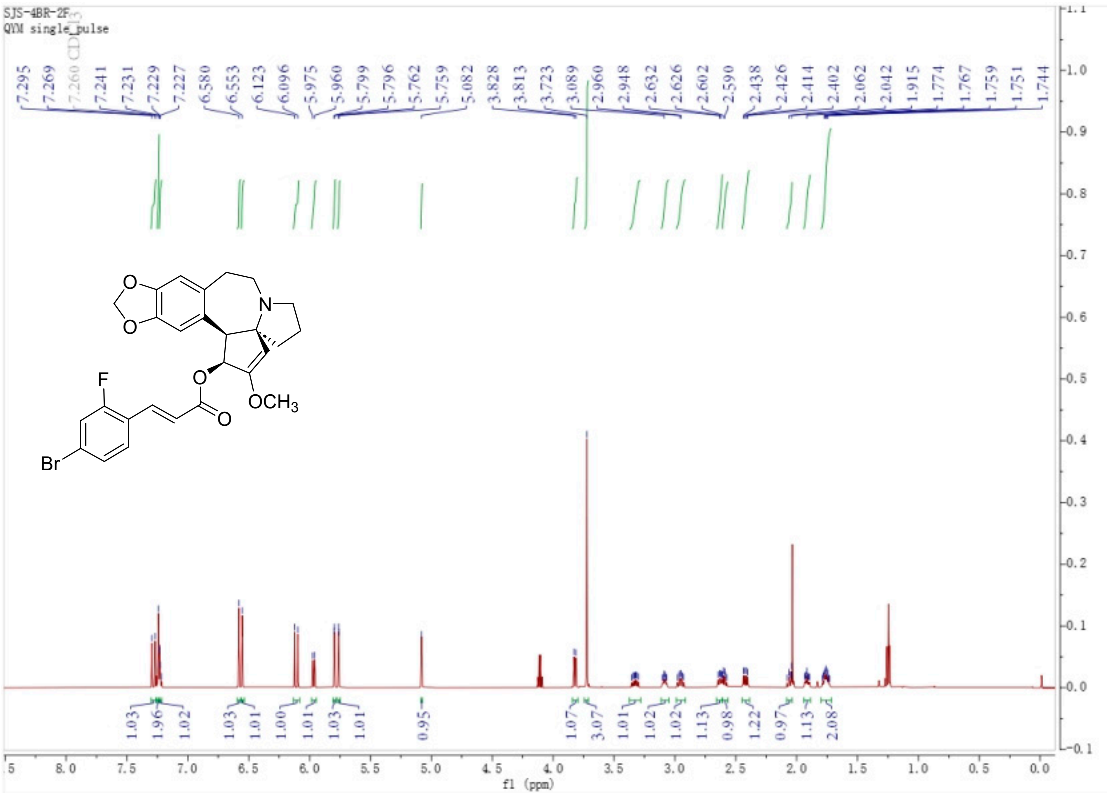

Figure S39. ^{1}H-NMR spectrum of **S12**.

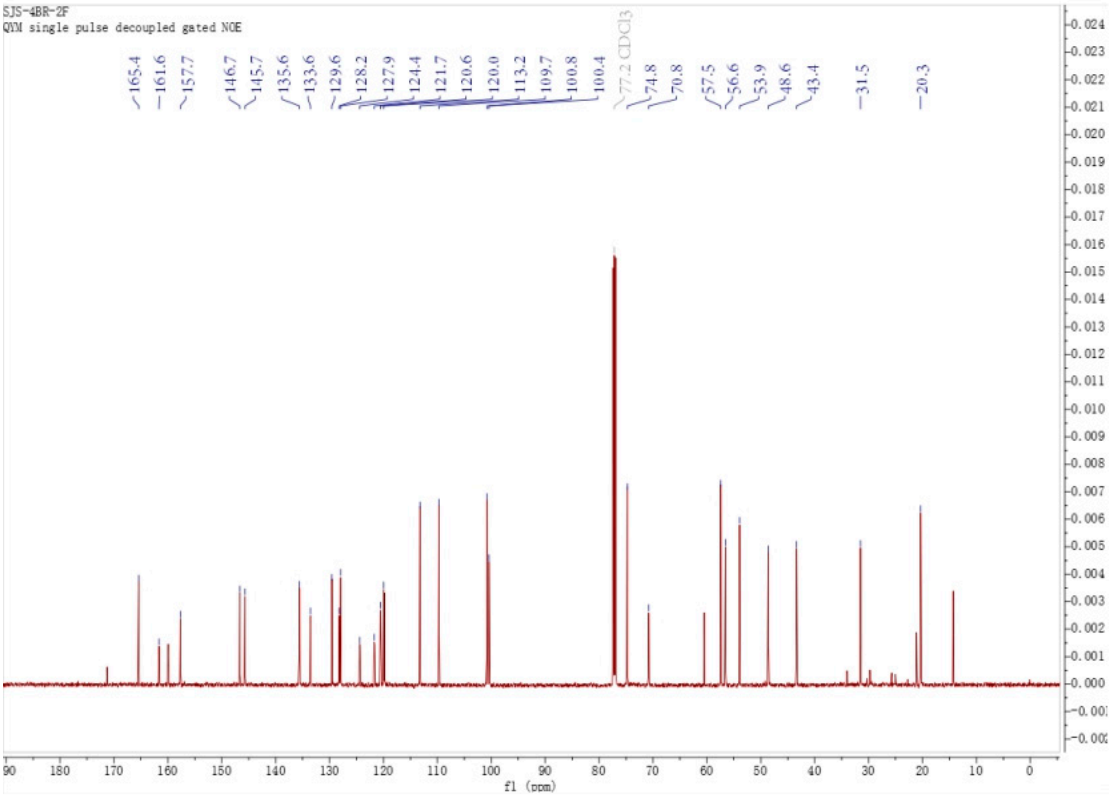

Figure S40. ^{13}C-NMR spectrum of **S12**.

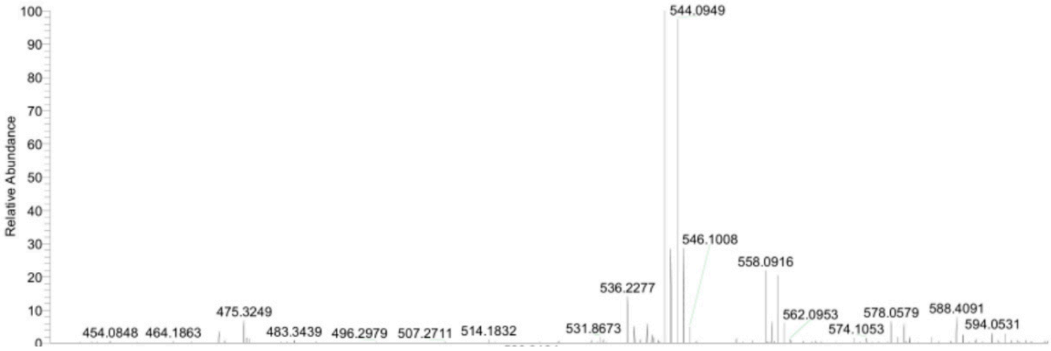

Figure S41. HR-ESI-MS spectrum of **S12**.

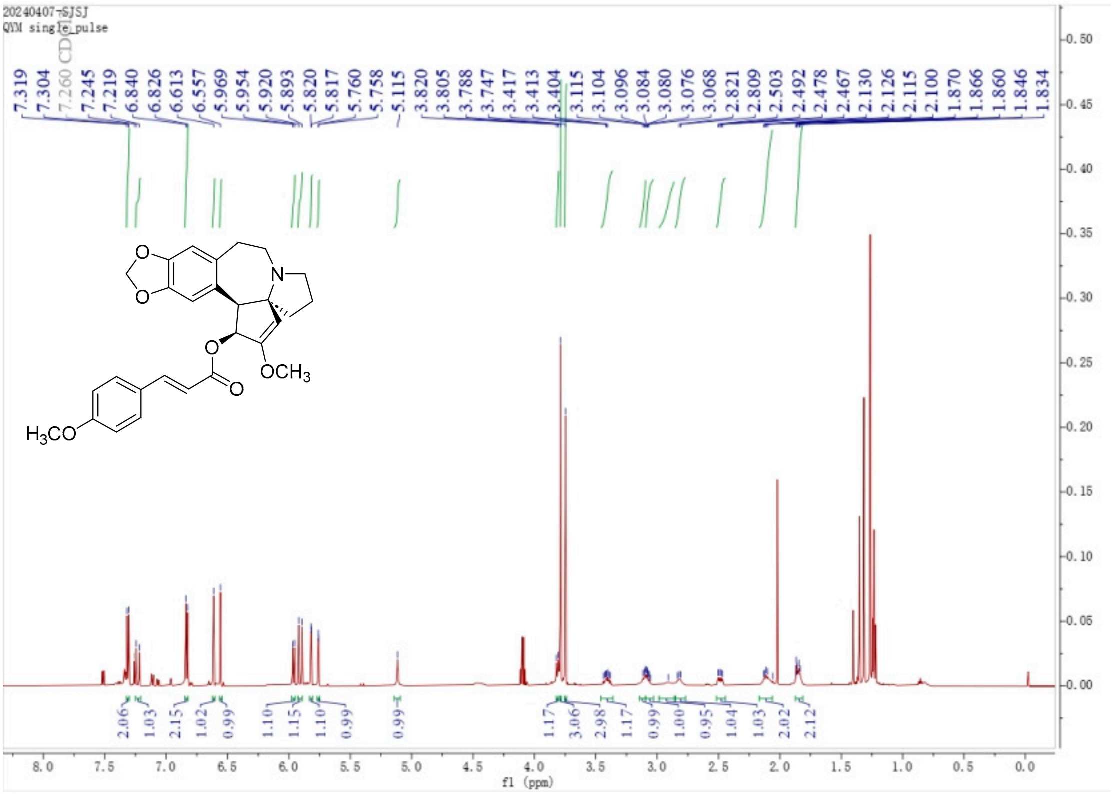

Figure S42. ^{1}H-NMR spectrum of **S13**.

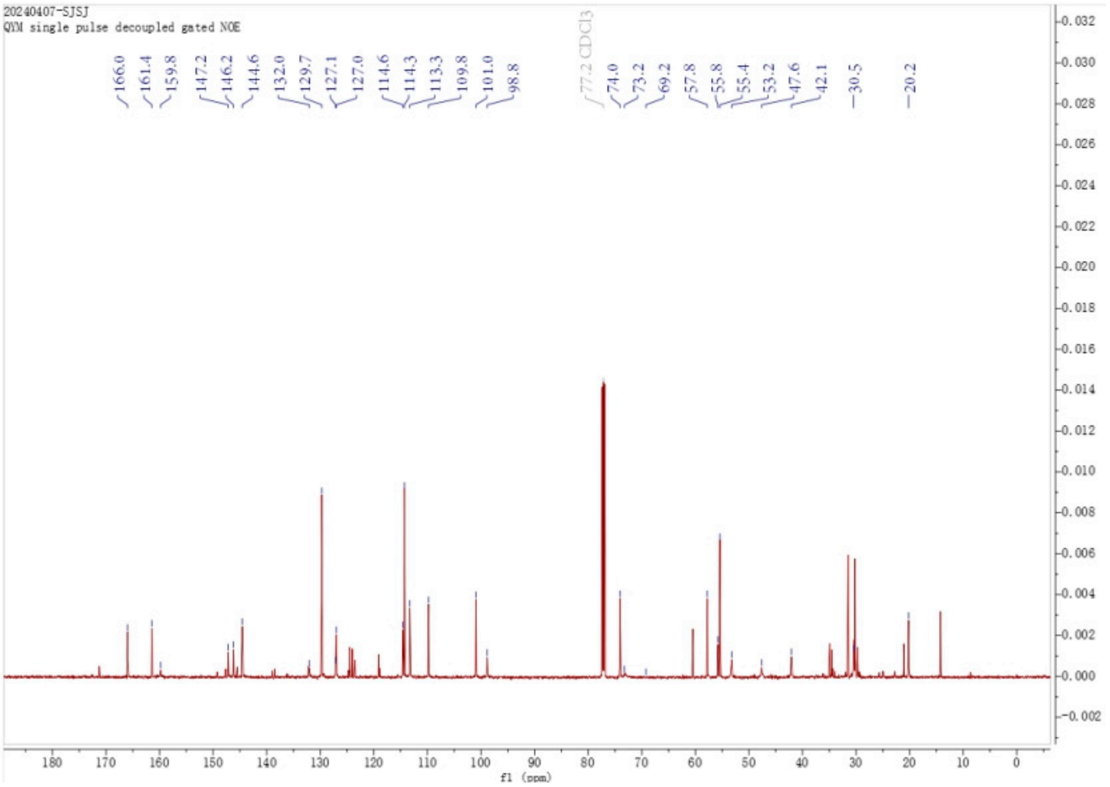

Figure S43. ^{13}C-NMR spectrum of **S13**.

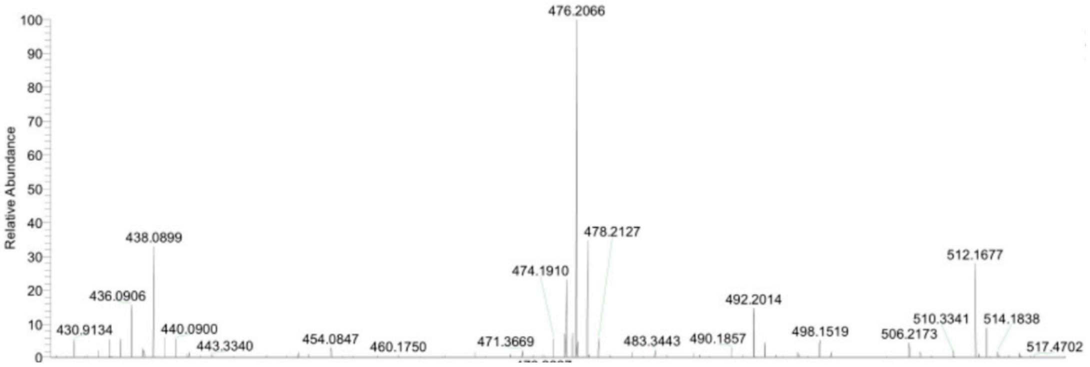

Figure S44. HR-ESI-MS spectrum of **S13**.

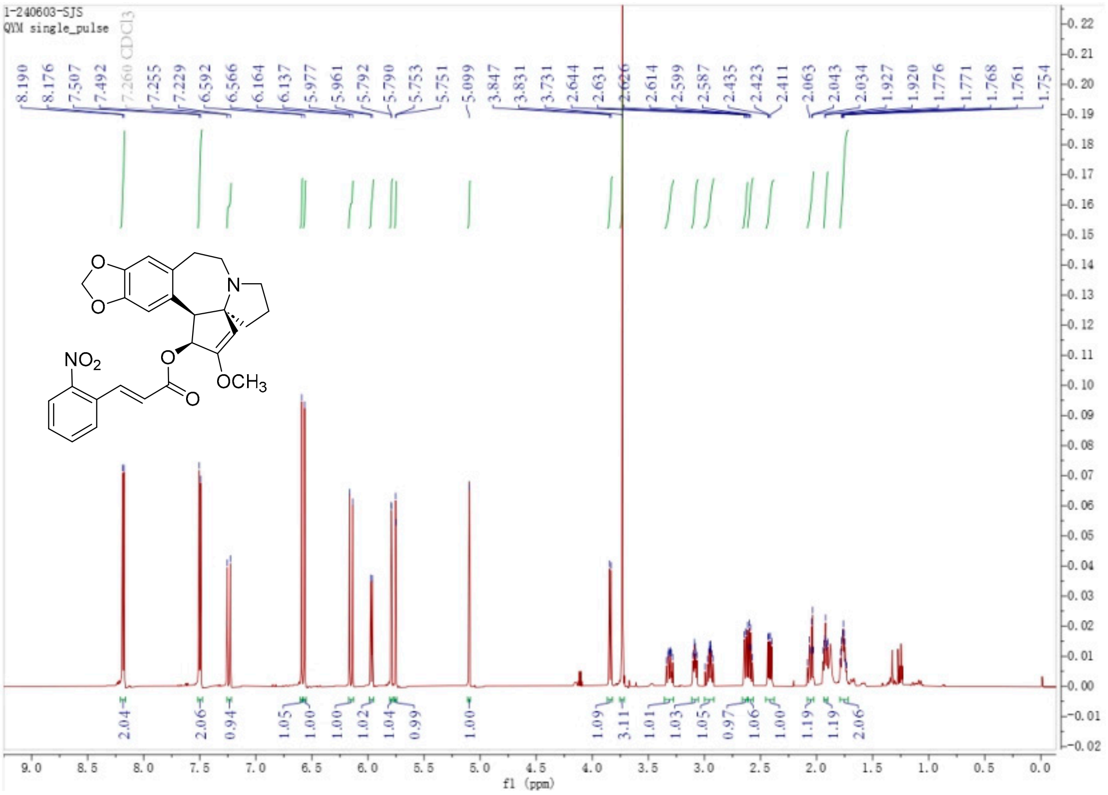

Figure S45. ^{13}C-NMR spectrum of **S14**.

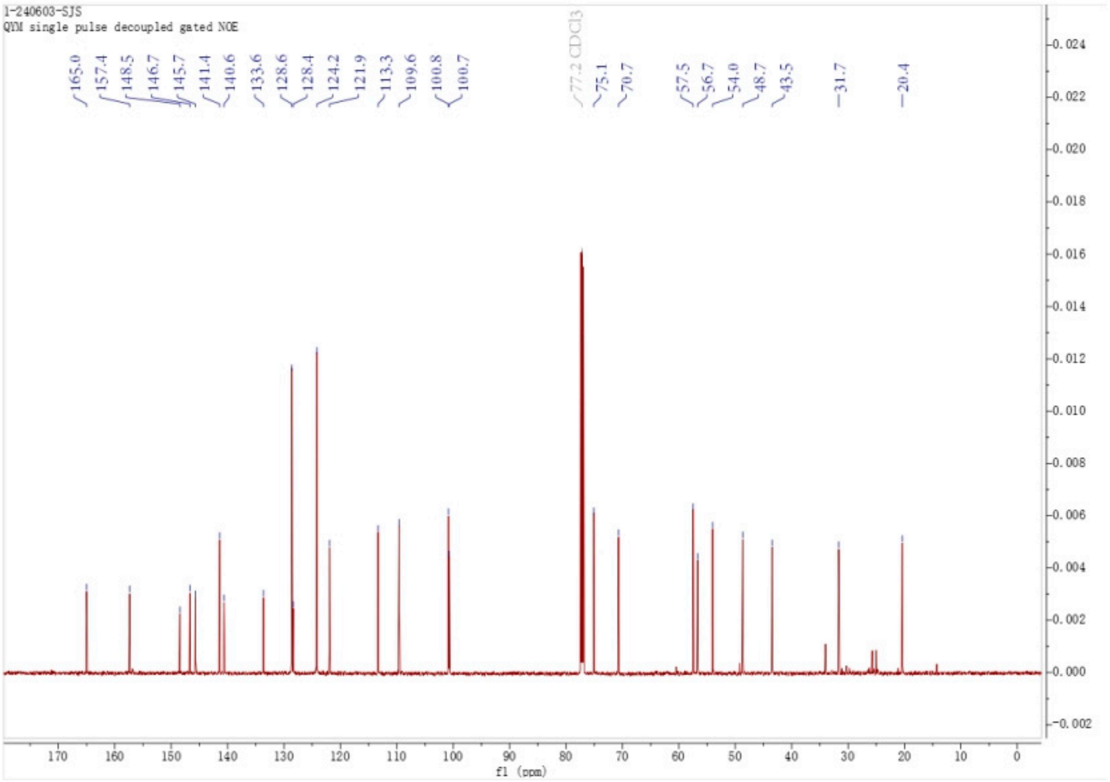

Figure S46. ^{13}C-NMR spectrum of **S14**.

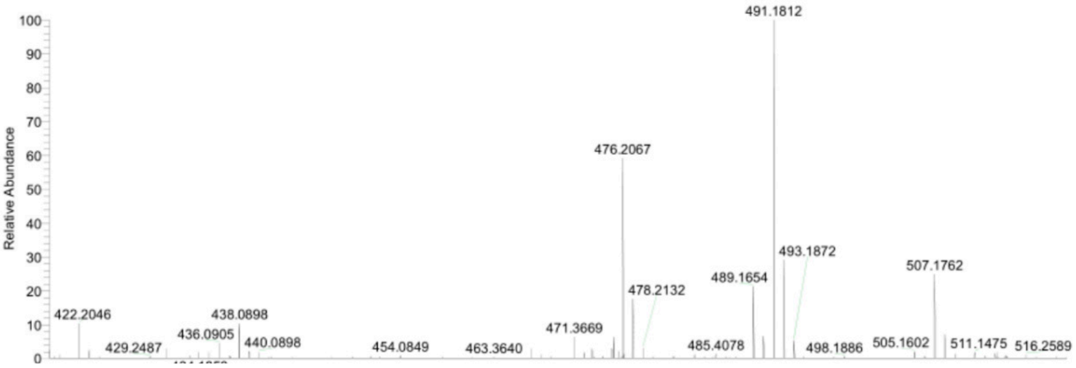

Figure S47. HR-ESI-MS spectrum of **S14**.

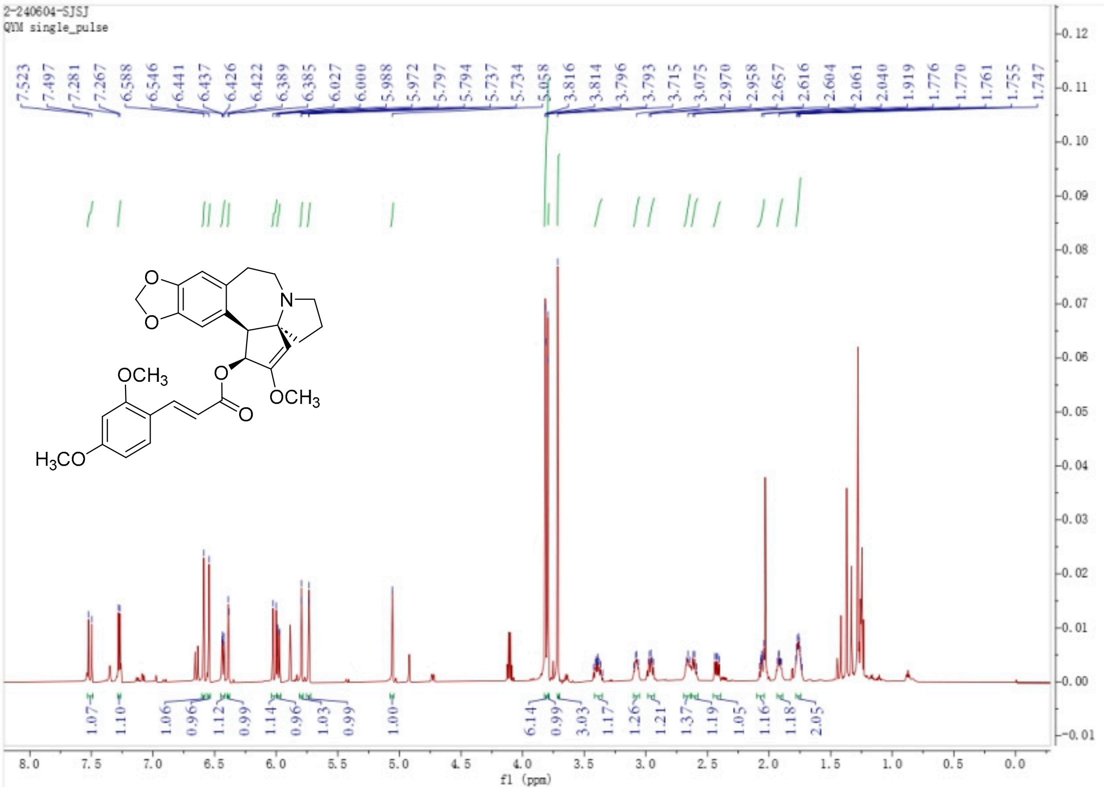

Figure S48. ^{1}H-NMR spectrum of **S15**.

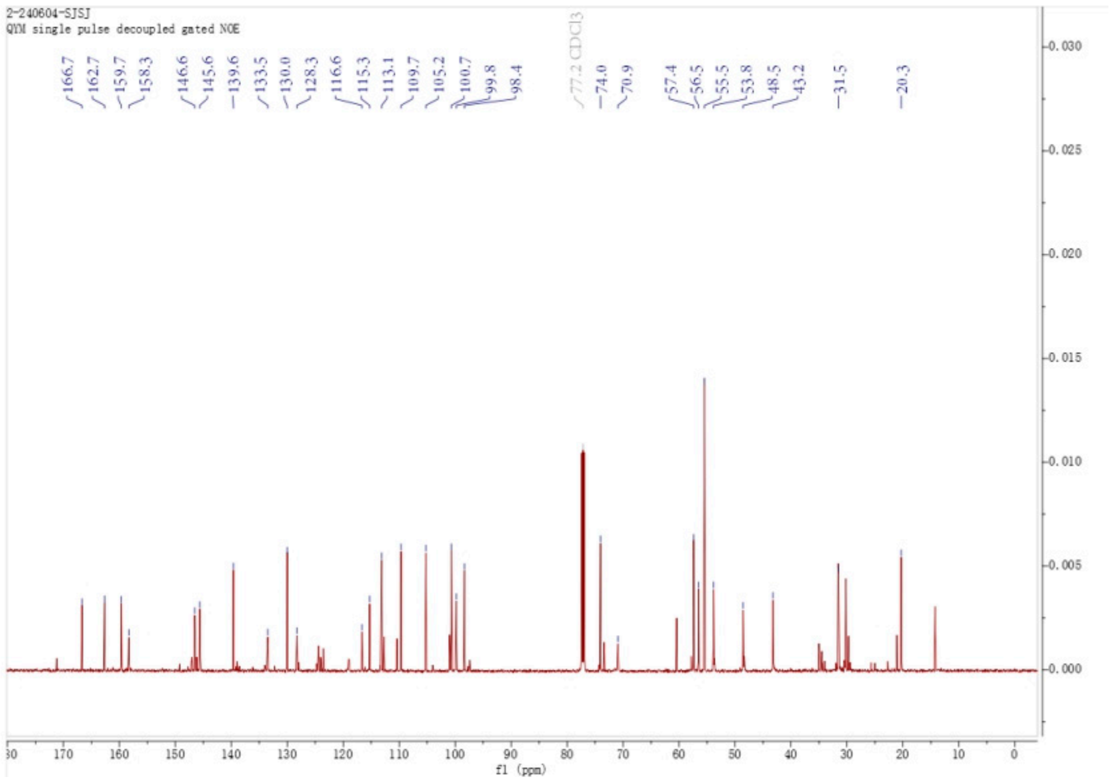

Figure S49. ^{13}C-NMR spectrum of **S15**.

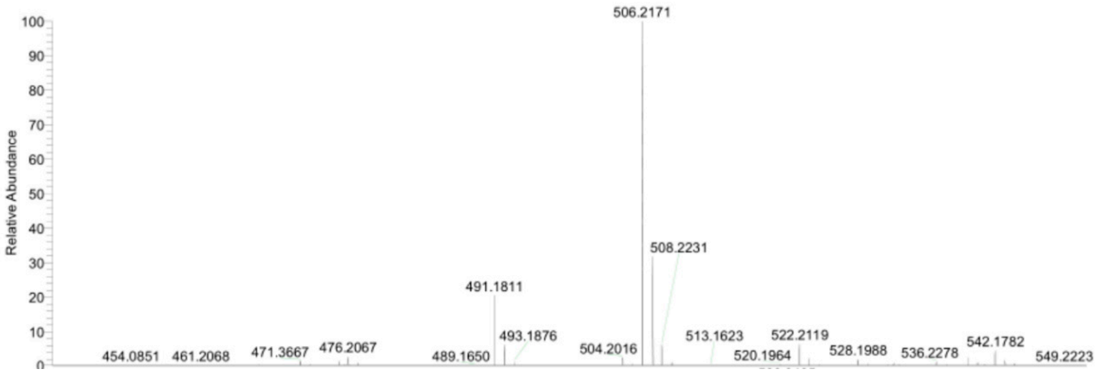

Figure S50. HR-ESI-MS spectrum of **S15**.

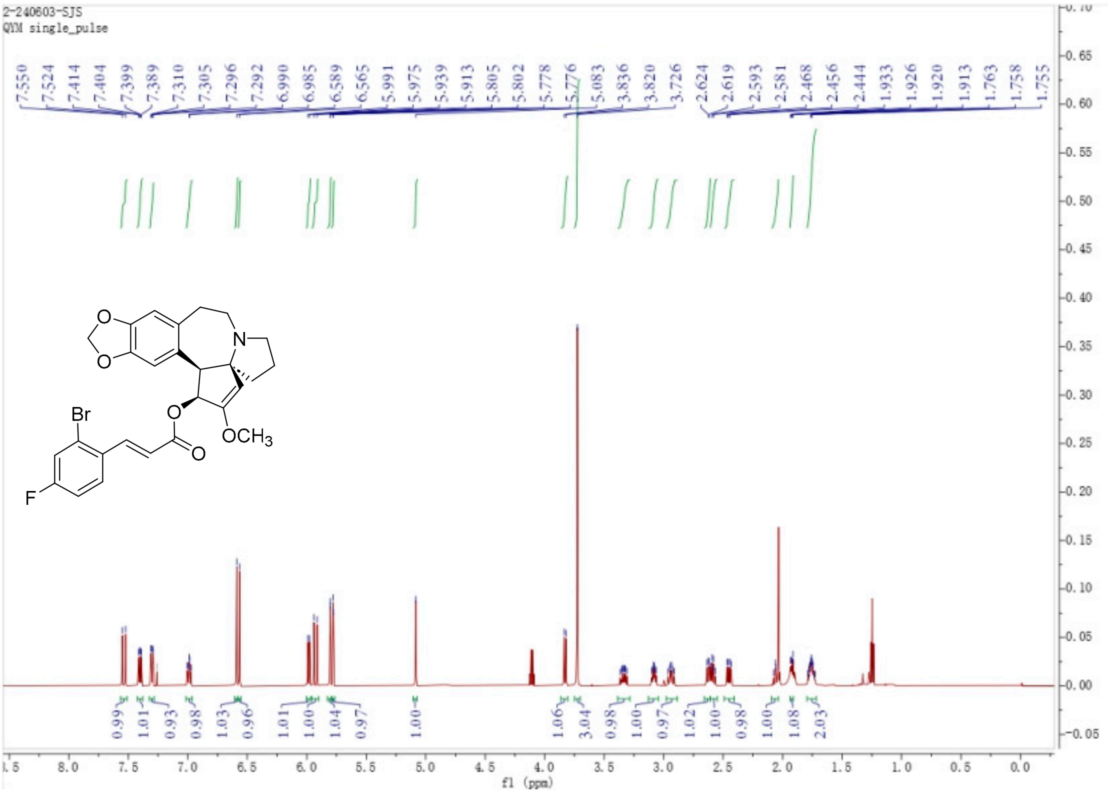

Figure S51. ^{1}H-NMR spectrum of **S16**.

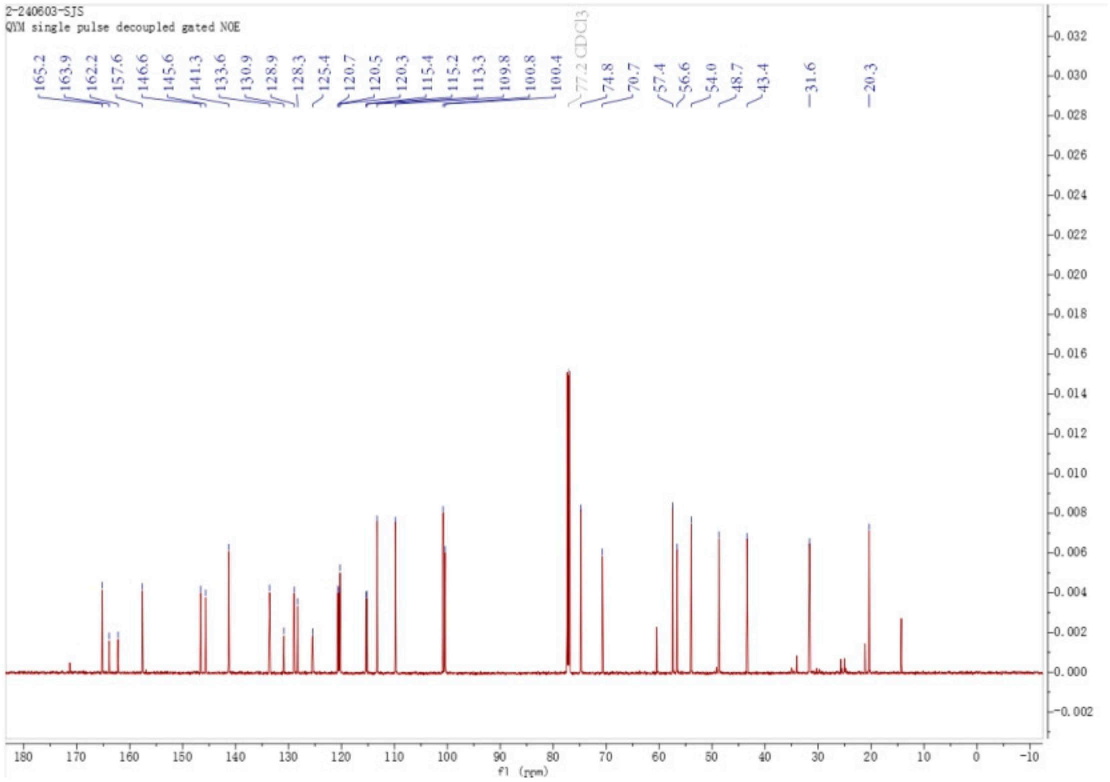

Figure S52. ^{13}C-NMR spectrum of **S16**.

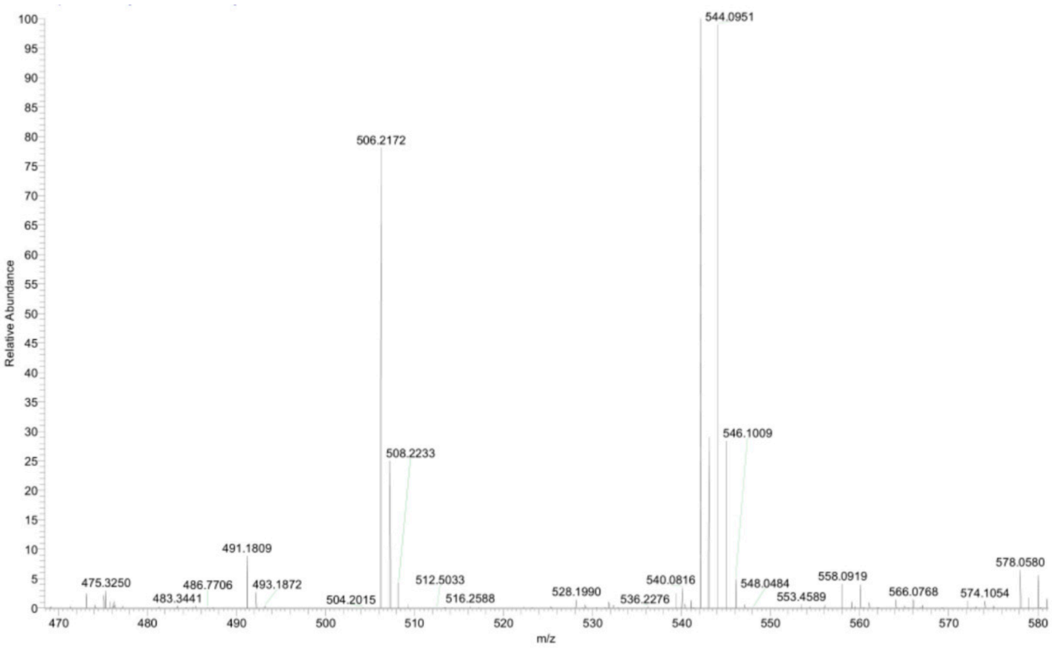

Figure S53. HR-ESI-MSspectrum of **S16**.

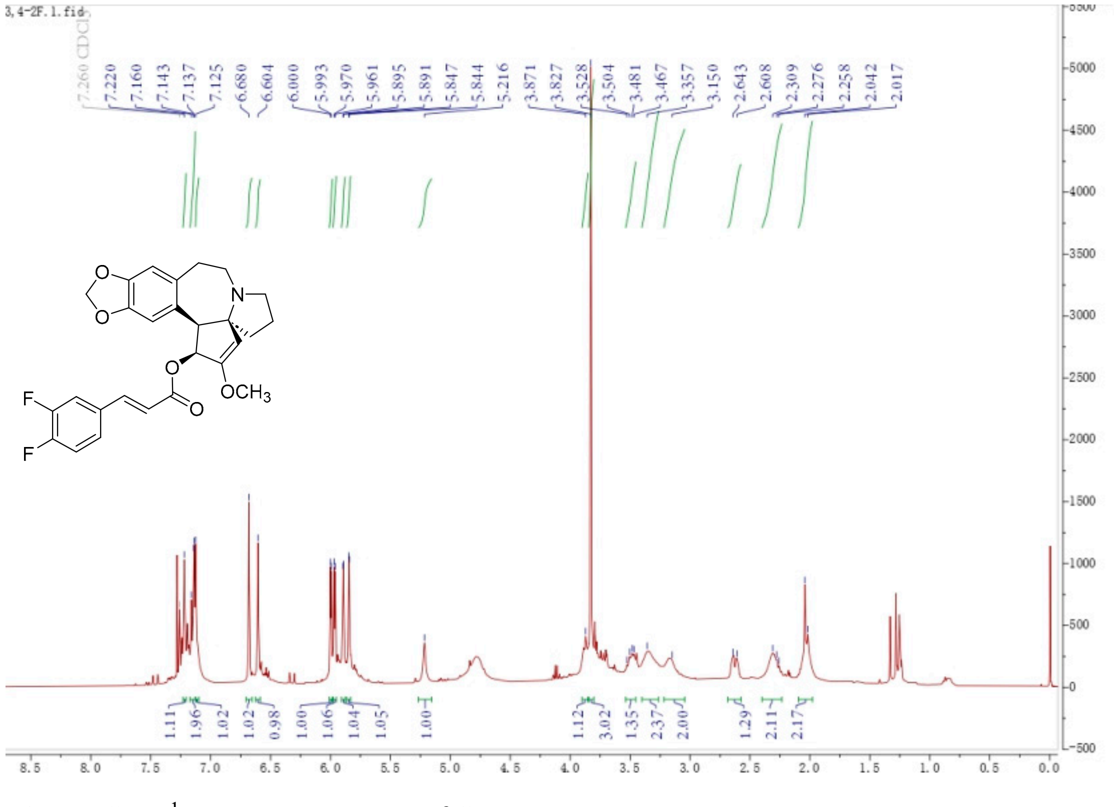

Figure S54. ^{1}H-NMR spectrum of **S17**.

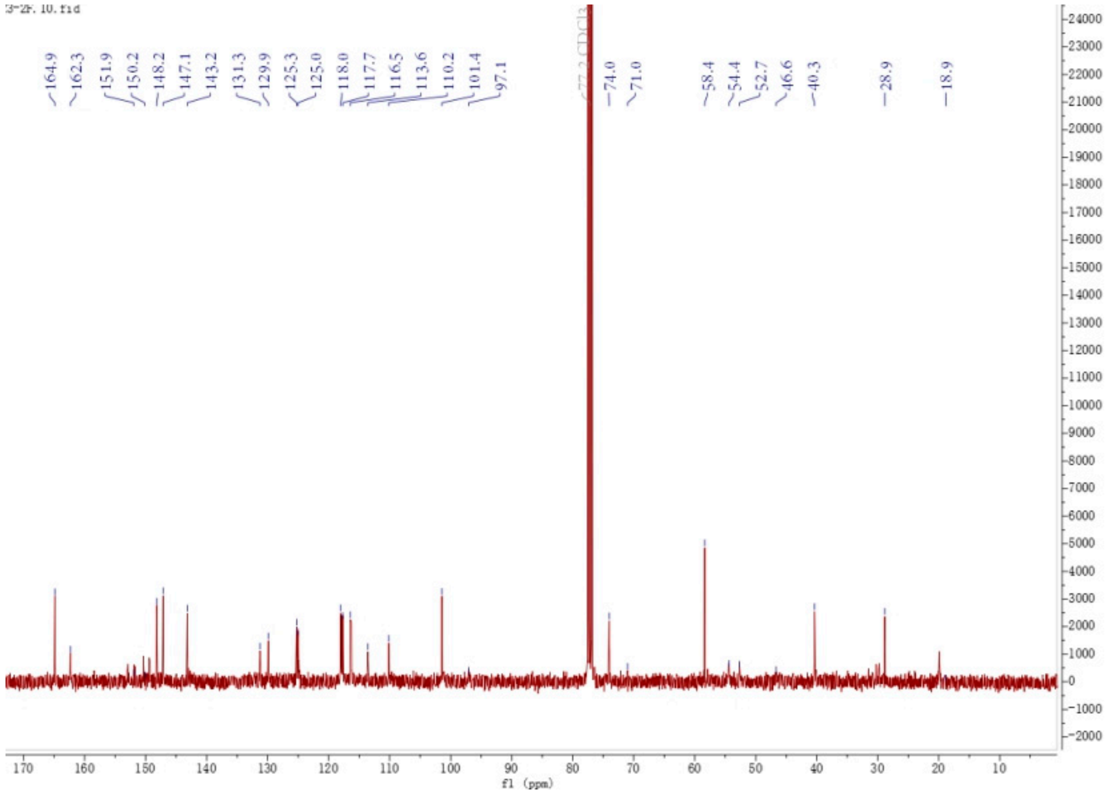

Figure S55. ^{13}C-NMR spectrum of **S17**.

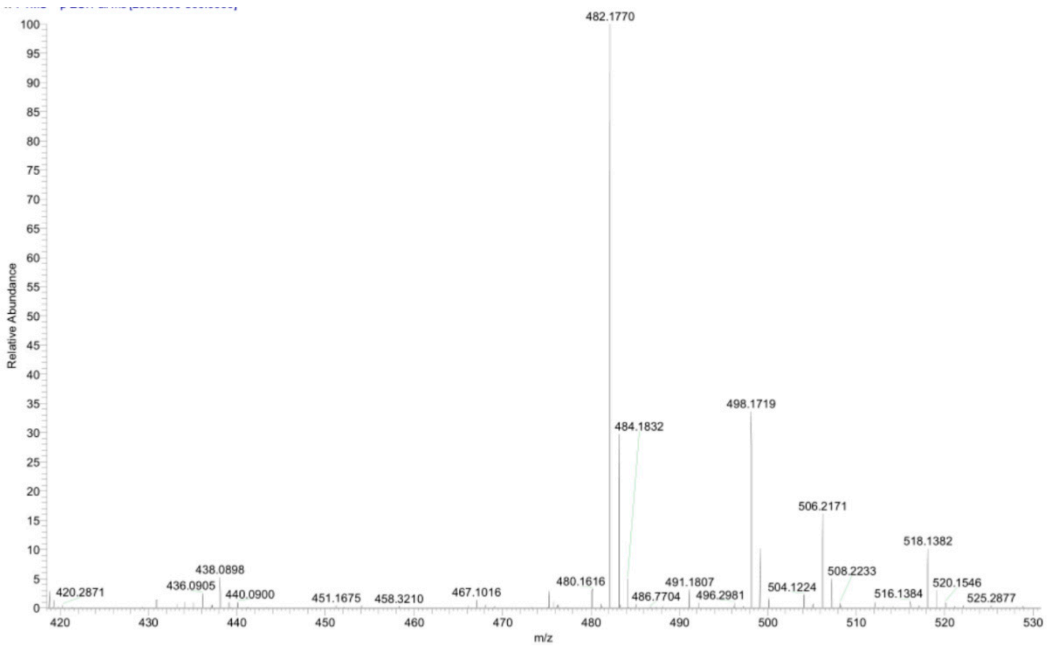

Figure S56. HR-ESI-MS spectrum of **S17**.
